# Supplementary material for: Structural insights into human brachyury DNA recognition and discovery of progressible binders for cancer therapy
Source: Nat Commun. 2025 Feb 14;16:1596. doi: 10.1038/s41467-025-56213-1 (PMC11828899; doi:10.1038/s41467-025-56213-1)
Supplement: Supplementary file 1 — Editorial Summary [file 41467_2025_56213_MOESM1_ESM.docx]

**Editorial Summary:**

This study describes structures of the transcription factor brachyury revealing the mechanism of DNA recognition. They identify fragments using X-ray fragment screening and optimize these into potent ligands with potential as cancer therapeutics.

**Peer review information:** *Nature Communications* thanks the anonymous reviewers for their contribution to the peer review of this work. A peer review file is available.
