# Supplementary material for: Structural insights into human brachyury DNA recognition and discovery of progressible binders for cancer therapy
Source: Nat Commun. 2025 Feb 14;16:1596. doi: 10.1038/s41467-025-56213-1 (PMC11828899; doi:10.1038/s41467-025-56213-1)

Supplementary Information For: Structural insights into human brachyury DNA recognition and discovery of progressible binders for cancer therapy

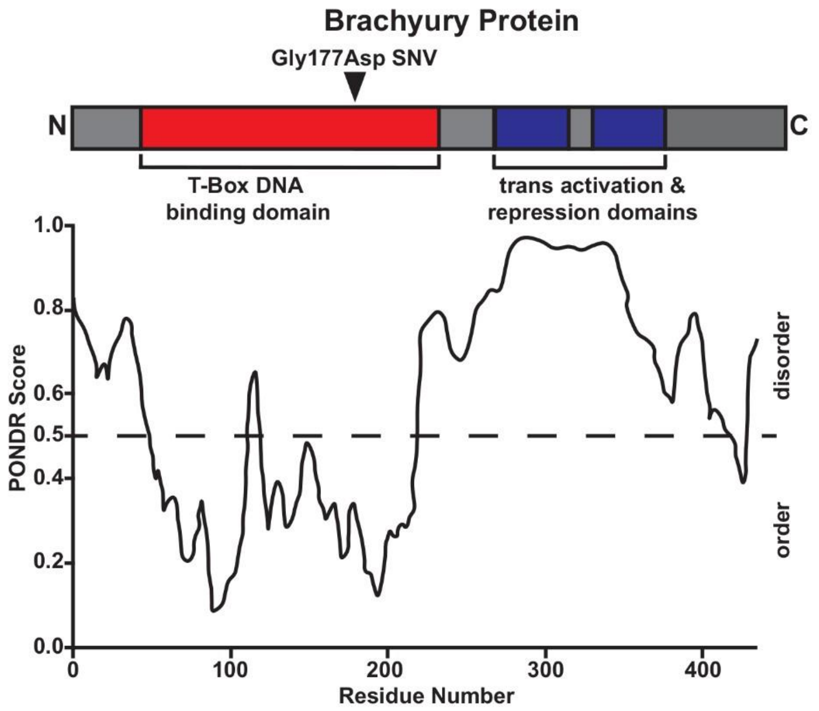

Supplementary Figure 1 – Analysis of structural disorder in the full length brachyury protein using the PONDR method. Predicted disordered regions map well with the boundaries of the know DNA binding domain and indicate that brachyury is largely disordered outside this domain.

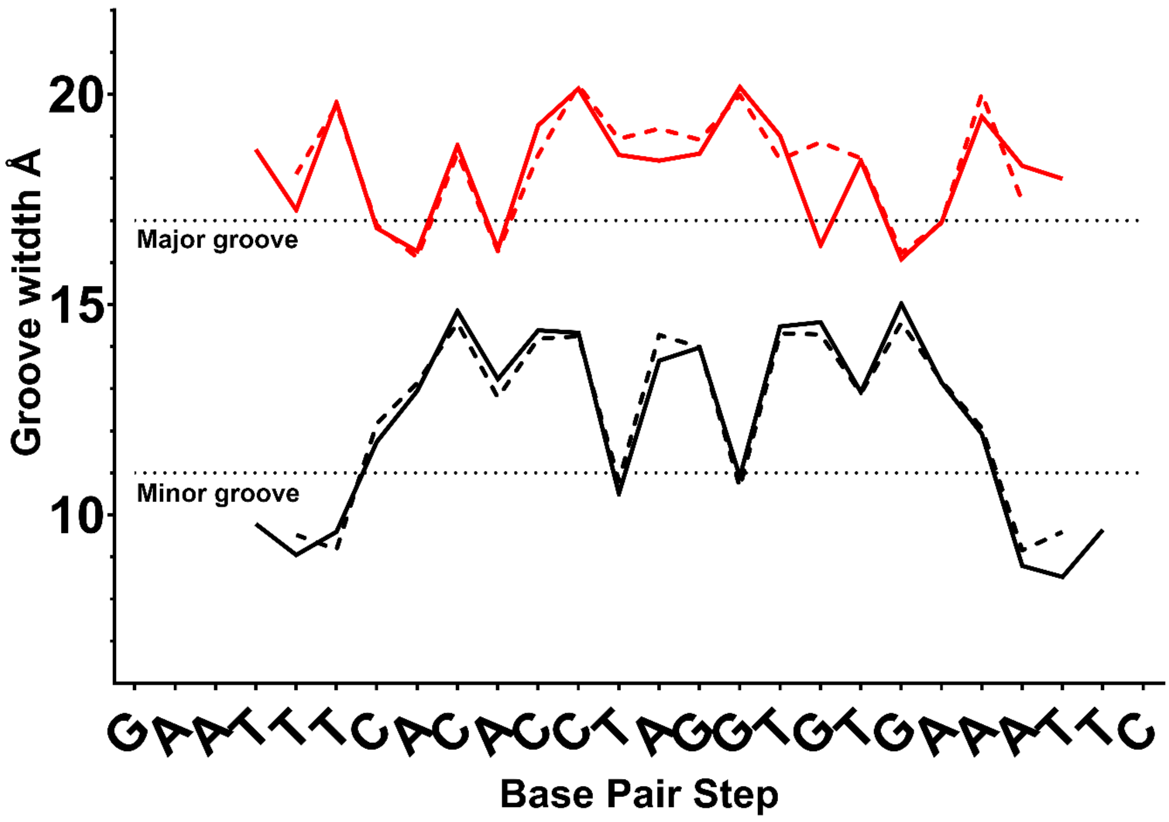

Supplementary Figure 2 - Plot of the measured major and minor groove widths of the DNA in the palindromic brachyury G177D DNA complex 6F59 (solid lines) and the WT DNA complex 6F58 (dashed lines). Major groove widths are measured phosphorus to phosphorus between each base and its partner 3 bases downstream. Minor groove widths are measured phosphorus to phosphorus between each base and its partner 4 bases upstream.

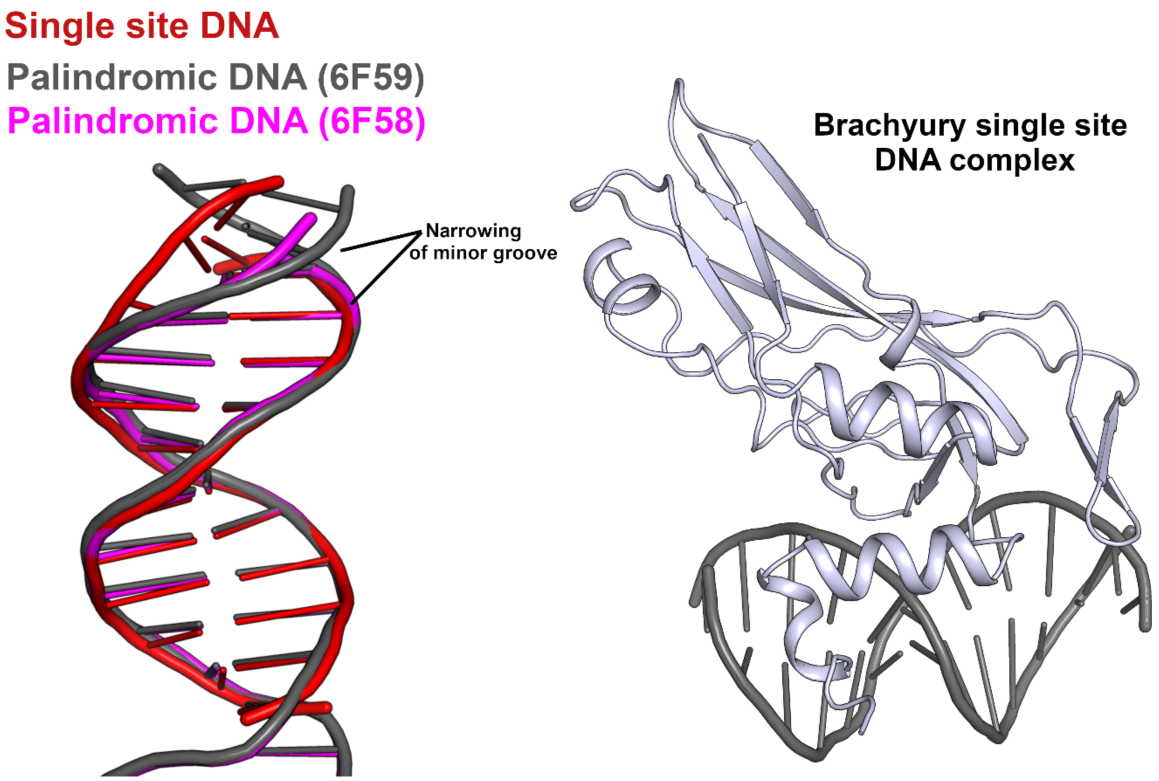

Supplementary Figure 3 - Structure of WT brachyury in complex with a single site DNA. The right-hand panel shows the overall structure in cartoon form which is very similar to a half site of the palindromic DNA complex. The left-hand panel shows a comparison of the three DNA molecules in the brachyury DNA complex structures which contain similar distortions from canonical B-form DNA with the exception of the end narrowing of the minor groove which is not present in the single site DNA complex.

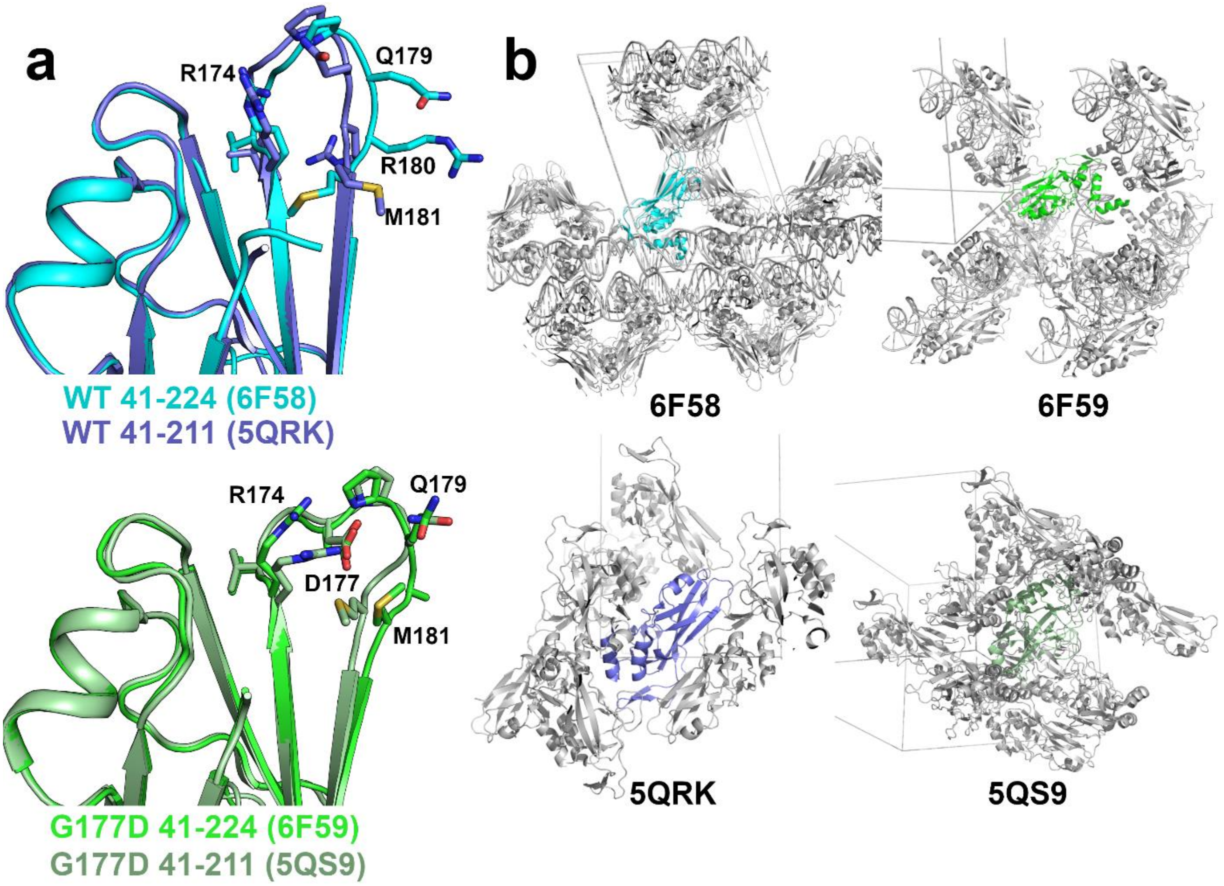

Supplementary Figure 4 – Analysis of crystal contacts in the brachyury crystals and impact on loop conformations. **a** Comparison of the DNA bound and DNA free WT (cyan and blue) and G177D (green and pale-green) brachyury crystal structures focussing on the regions in the vicinity of the G177D variant (same view as Figure 2A). **b** Overview of the crystal contacts present in the DNA bound (top row) and DNA free (bottom row) crystal forms. WT crystals are shown on the left and G177D on the right. Crystallographic neighbours are shown for molecules ± 1 unit cell and within 5 Å of the reference chain which is coloured as for panel A.

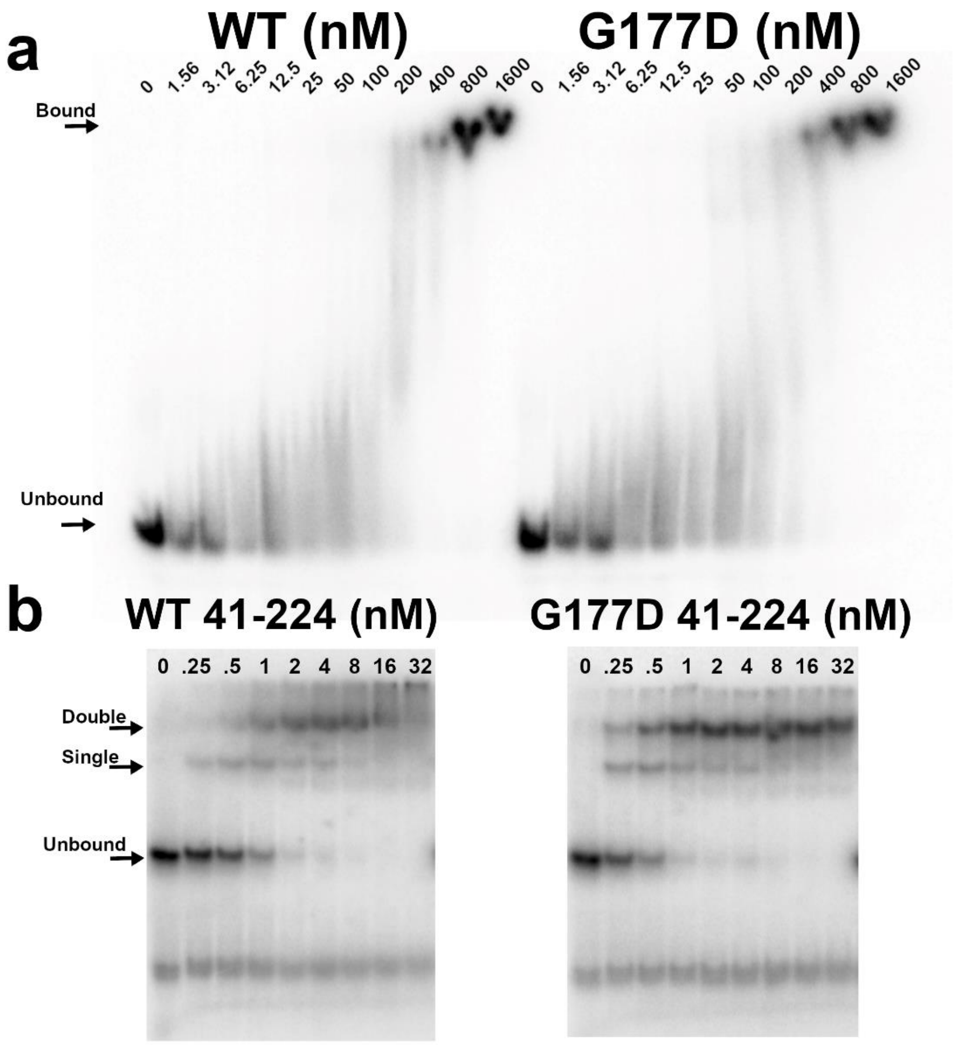

Supplementary Figure 5 – **a** Representative gel of full-length WT and G177D brachyury binding to an oligonucleotide containing a single T-box binding element. The results of the quantification of three replicates of this gel are shown in Figure 2c. **b** Representative gel of WT and G177D brachyury DNA binding domain (41-244) binding to a 50 bp DNA with palindromic repeat of the T-box recognition element, a similar banding pattern is observed to that in Figure 2B although the bands are closer together consistent with the smaller mass difference for the shorter construct.

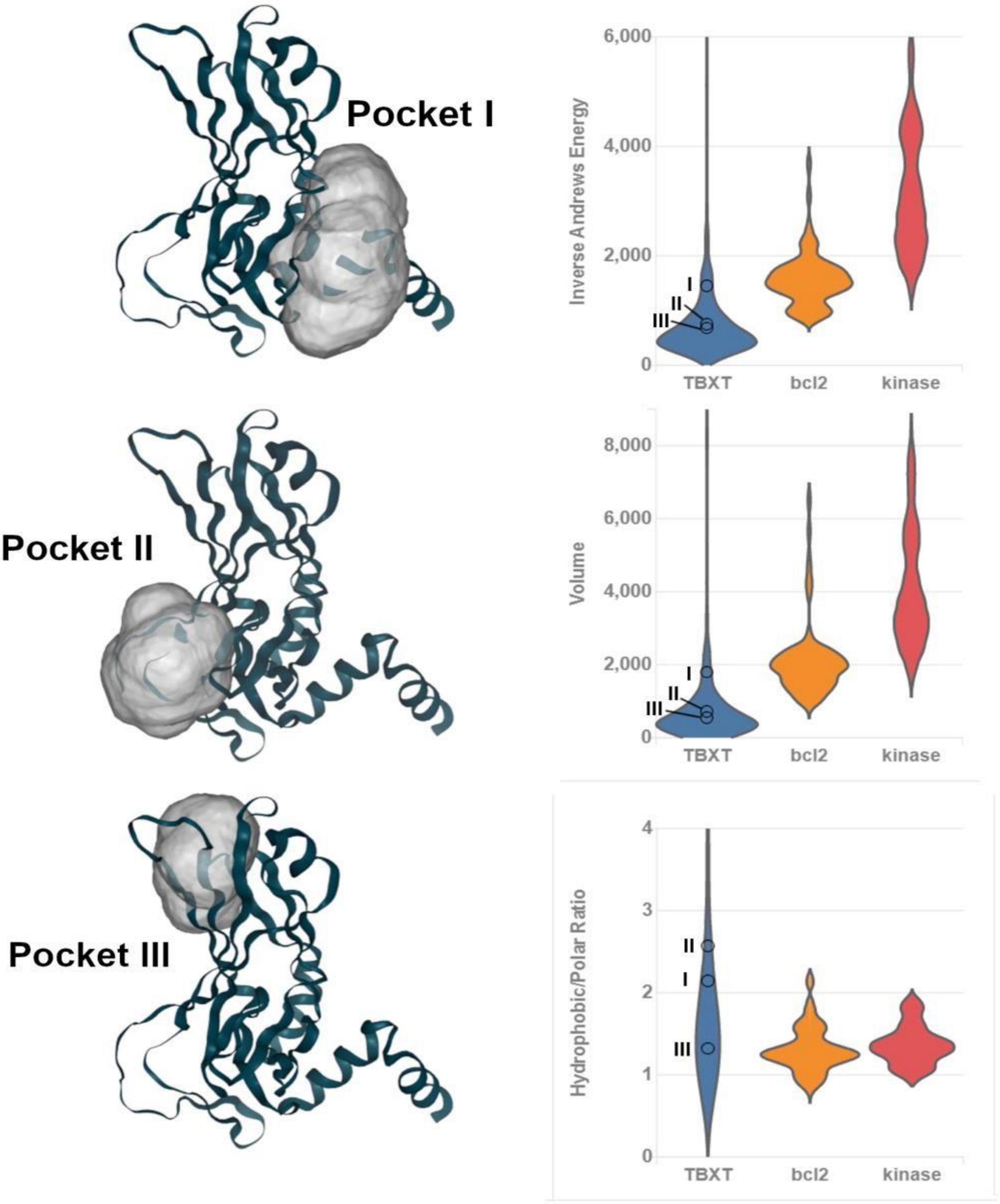

Supplementary Figure 6 – Analysis of the ligandability potential of pockets on brachyury using the tools available at the CANSAR web server. The left panel shows representations of three pockets identified in the brachyury G177D DNA complex structure (6f59:chainA). The graphs on the right show pocket properties relating to ligandability with the brachyury pockets marked. Representative distributions of challenging (bcl2 – orange) and tractable (kinase – Red) pockets are shown for reference.

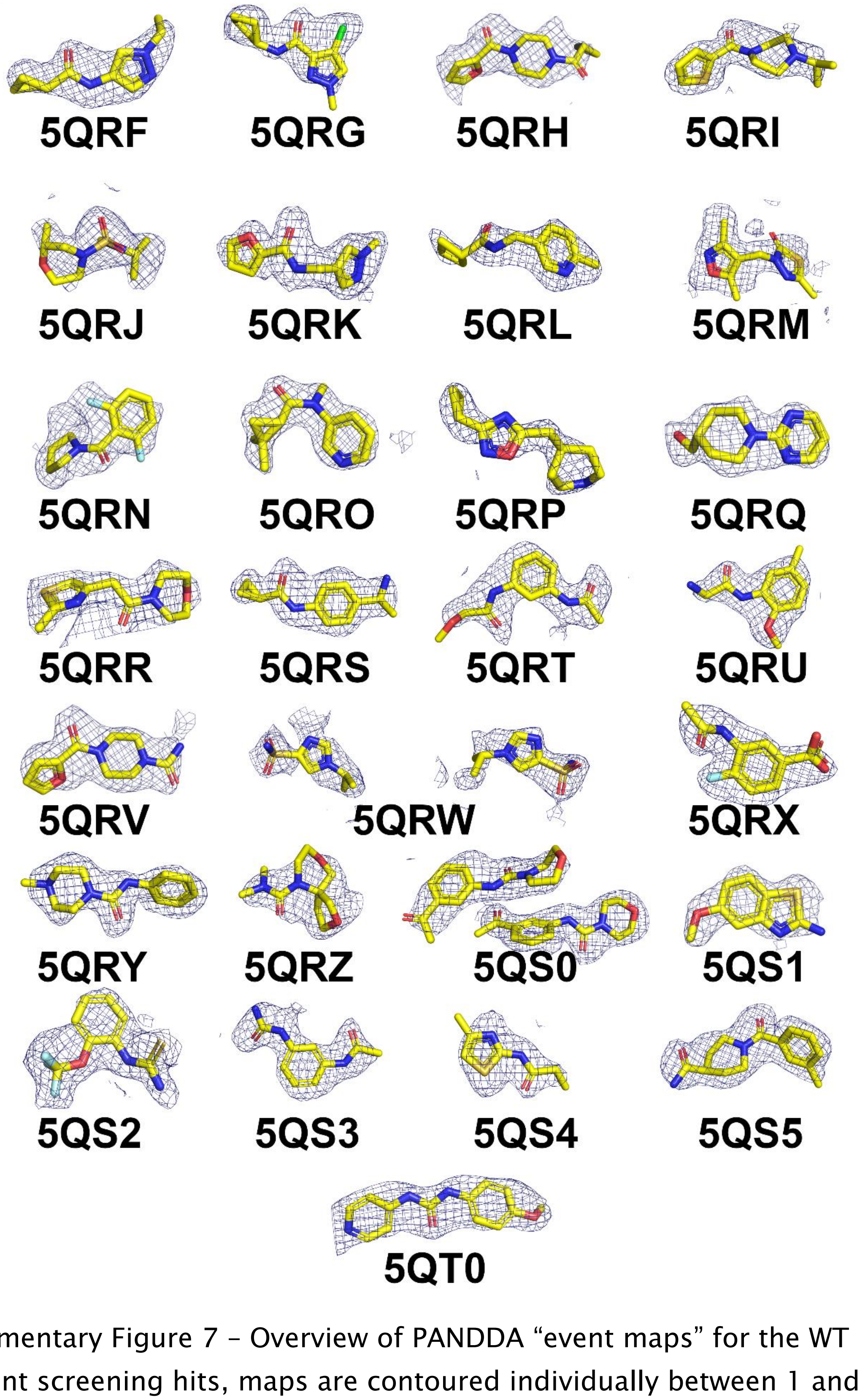

Supplementary Figure 7 – Overview of PANDDA "event maps" for the WT fragment screening hits, maps are contoured individually between 1 and 1.5 σ. PDB codes are displayed below each fragment.

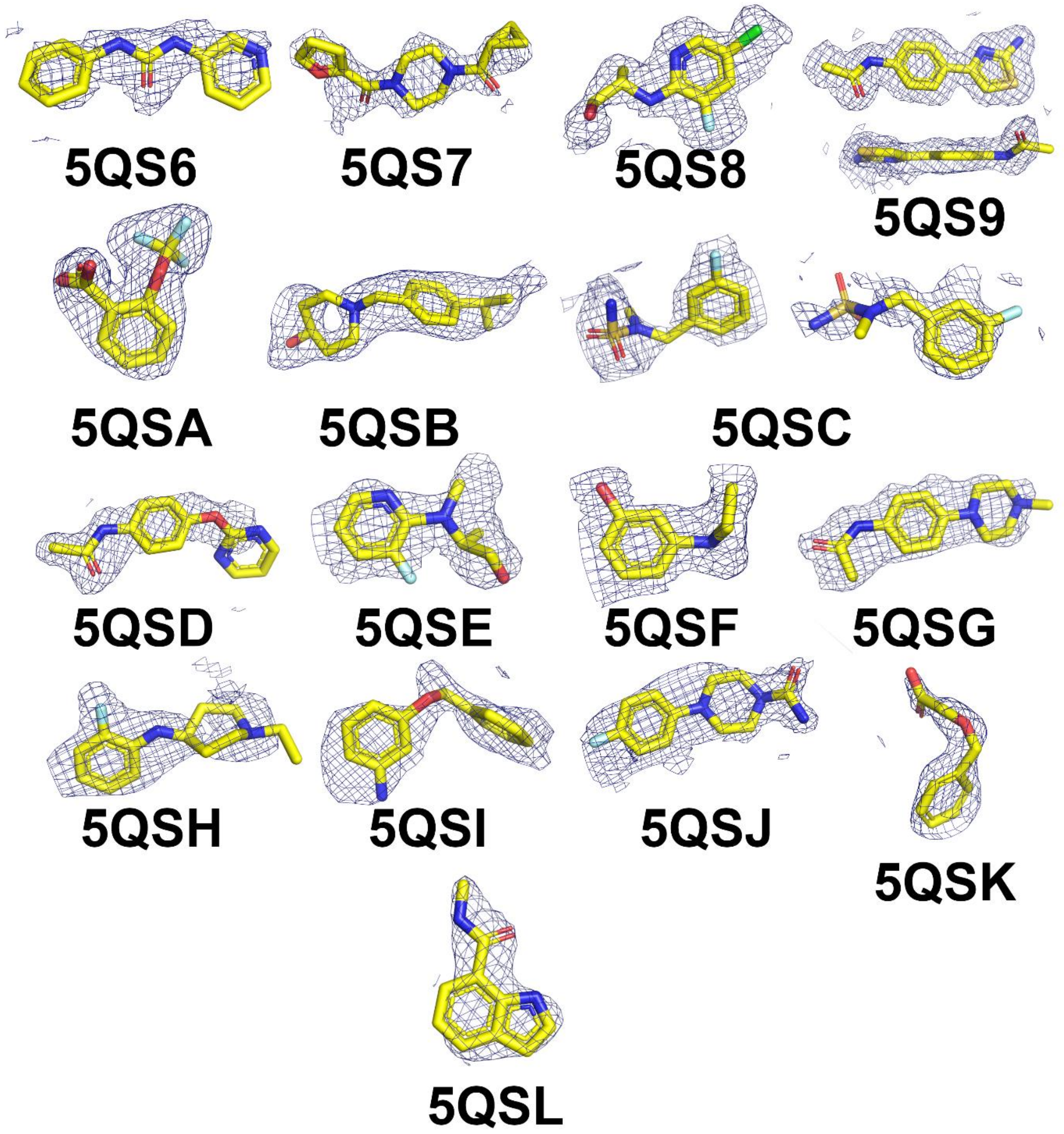

Supplementary Figure 8 – Overview of PANDDA "event maps" for the G177D fragment screening hits, maps are contoured individually between 1 and 1.5 σ. PDB codes are displayed below each fragment.

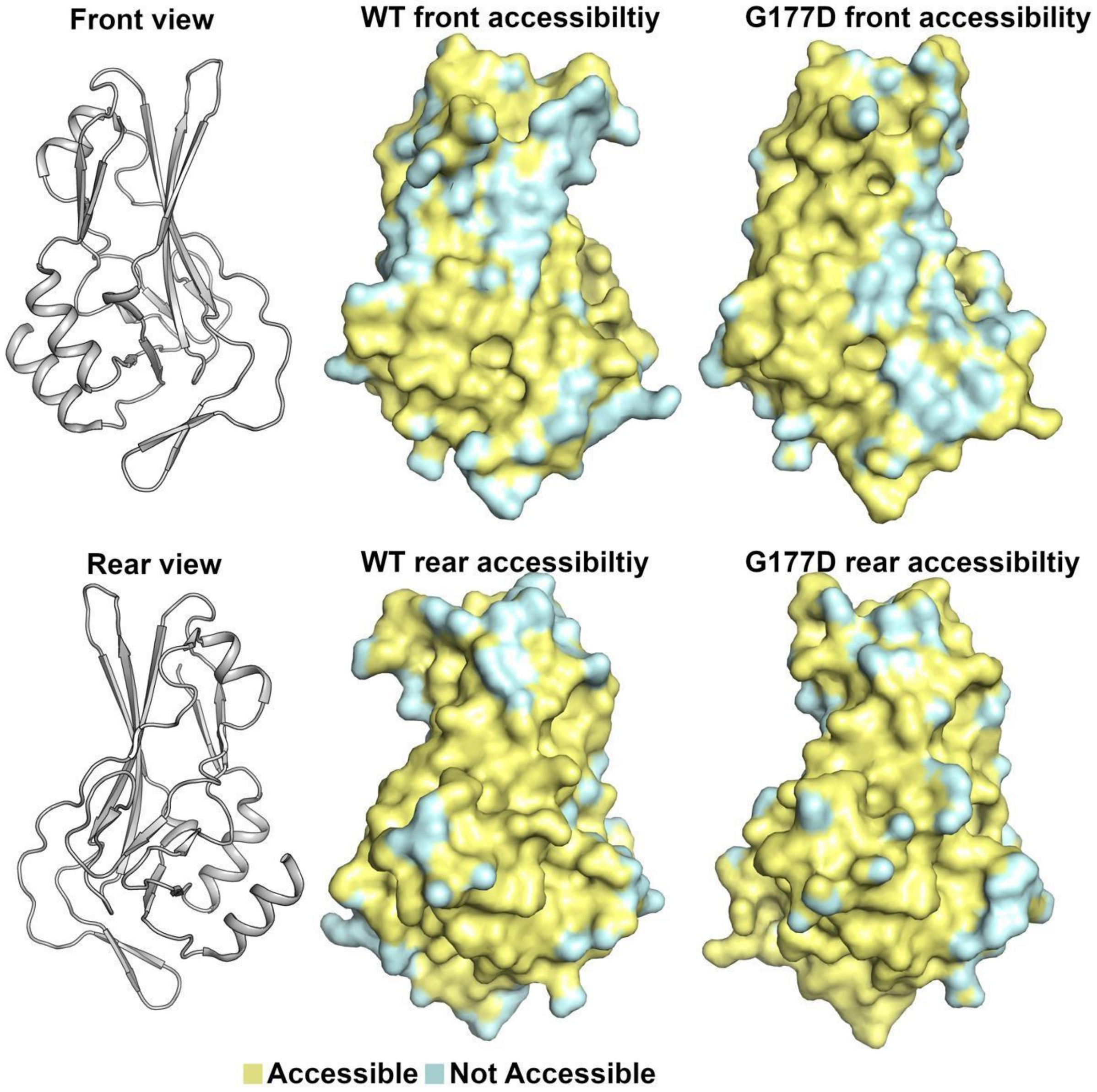

Supplementary Figure 9 – Accessibility of the various surfaces and pockets in the WT and G177D crystals. Inaccessible regions are defined as surface atoms within 4 Å of an atom in a crystal contact.

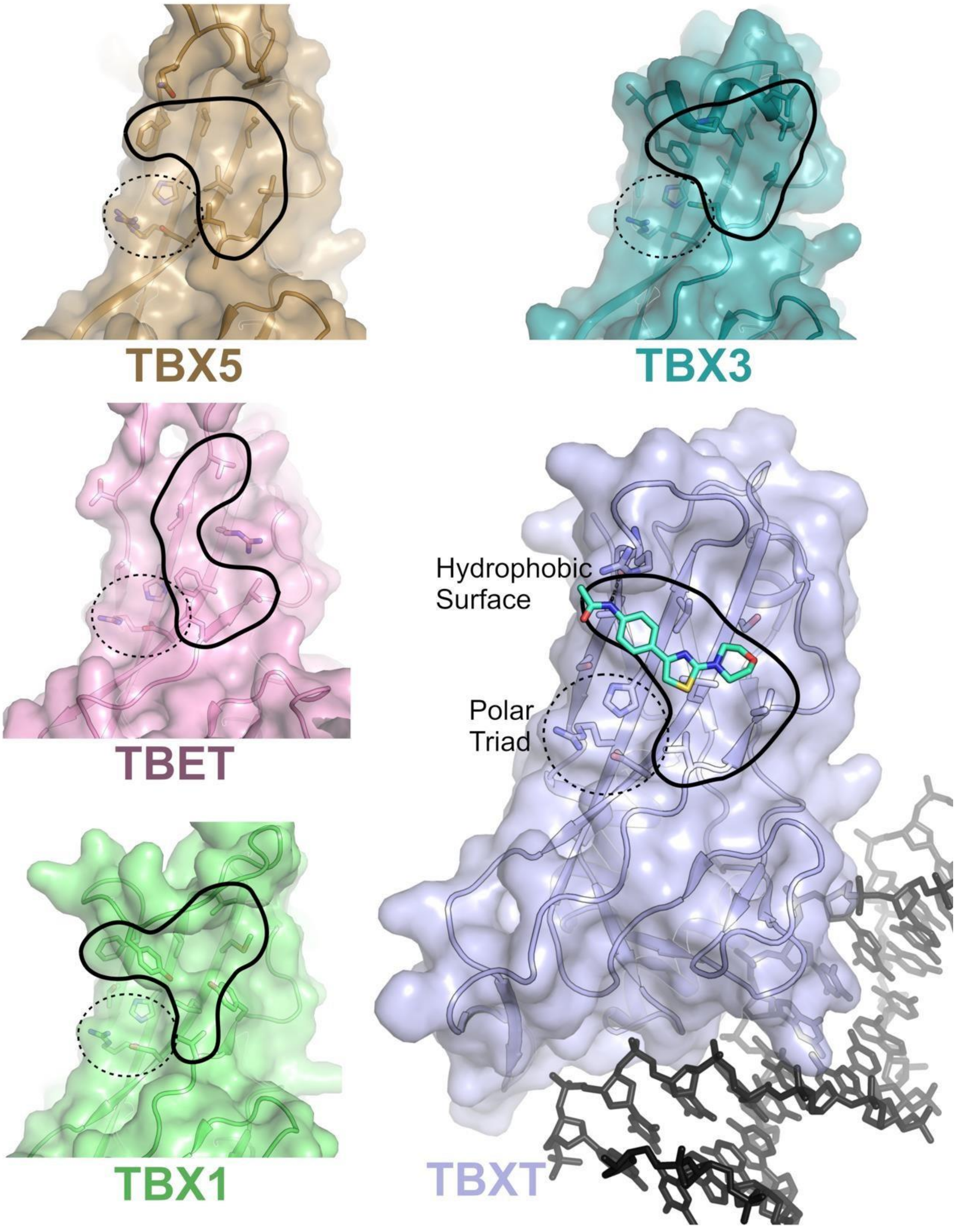

Supplementary Figure 10 – Overview of pocket A’ in brachyury and other T-Box family members. The brachyury A’ pocket (shown in grey with surface view and ligand for reference) is distant from the DNA binding interface and contains a polar triad and an unusual cluster of surface exposed hydrophobic residues. Both of these features are conserved across the T-Box family.

Ligand	Ka1 (1/Ms)	Kd1 (1/s)	Ka2 (1/RUs)	Kd2 (1/s)	Rmax (RU)	tc	Chi² (RU²)
WT brachyury	2.9E5 ± 4.9E2	3.9E-4 ± 1.3E-6	2.47 ± 0.77	19.9 ± 5.3	167 ± 0.15	1.34E12	2.57
G177D brachyury	2.2E5 ± 5.1E2	3.9E-4 ± 1.7E-6	0.61 ± 0.1	6.87 ± 1.2	262 ± 0.4	3.34E11	7.53

Supplementary Table 1A – Parameters from kinetic fitting of the SPR data in Figure 2D to a bivalent analyte model. Errors are shown as ± the standard error. Tc is the mass transfer coefficient.

Ligand	Kd (M)	Rmax (RU)	Offset	Chi² RU²
WT brachyury	1.48E-8 ± 2.4E-9	161.1 ± 5.8	5.7 ± 6.3	21.1
G177D brachyury	1.79E-8 ± 2.8E-9	250.4 ± 8.5	6.5 ± 8.7	49.9

Supplementary Table 1B – Parameters from fitting the SPR data in Figure 2D to a concentration response (steady state affinity) model. Errors are ± standard error.

PDB	Ligand	Pocket	2Fo-Fc map (1σ)	Location
5QT0	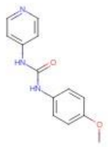 Z321318226	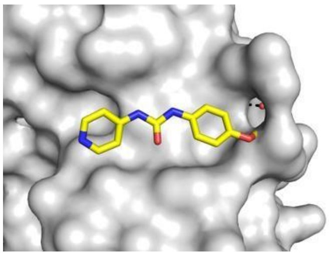	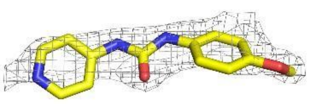 2.10 Å	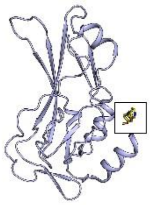 Pocket C
5QS6	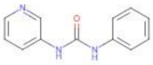 Z44592329	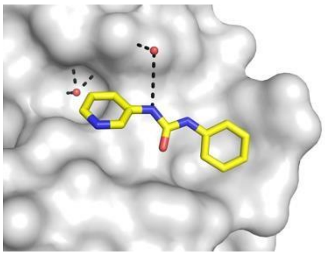	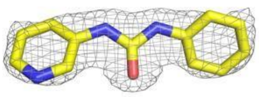 1.67 Å	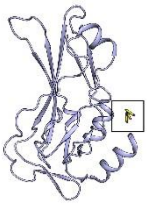 Pocket C
5QS7	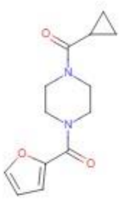 Z32327641	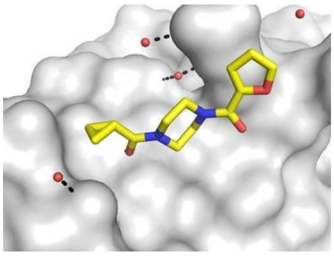	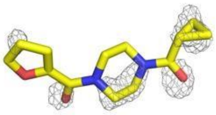 1.66 Å	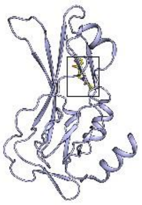 G177D other
5QS8	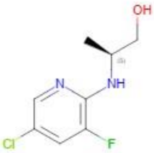 Z1432018343	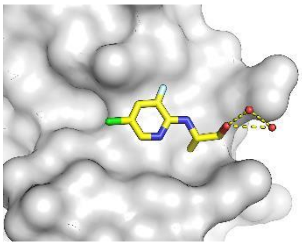	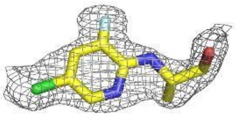 1.47 Å	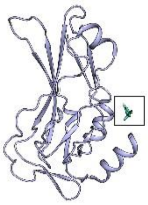 Pocket C
5QS9	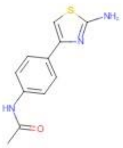 Z48847594	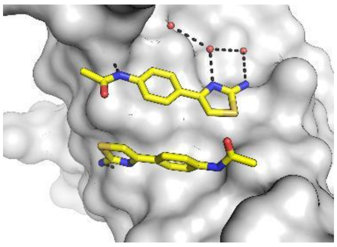	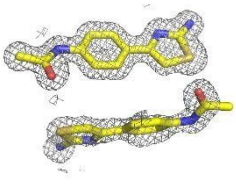 1.43 Å	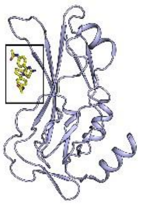 Pocket A’
5QSA	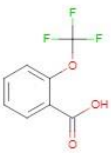 Z2856434778	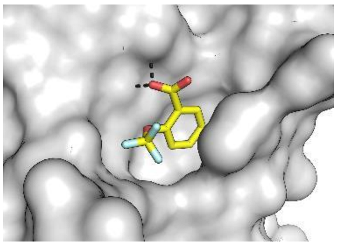	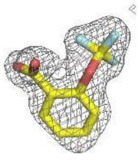 1.55 Å	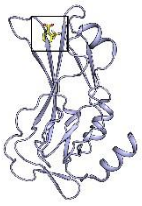 Pocket D

PDB	Ligand	Pocket	2Fo-Fc map (1σ)	Location
5QSB	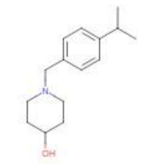 Z2856434874	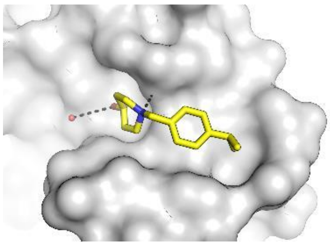	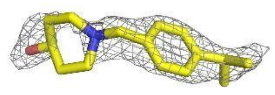 1.82 Å	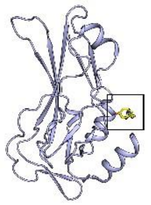 Pocket C
5QSC	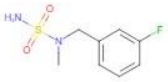 Z300245038	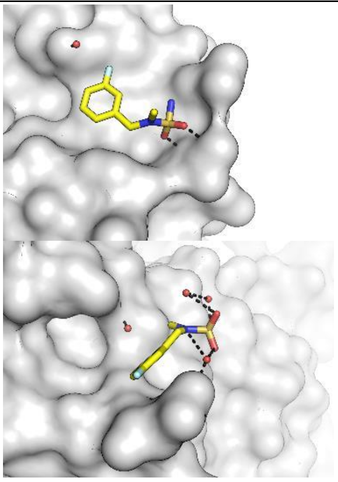	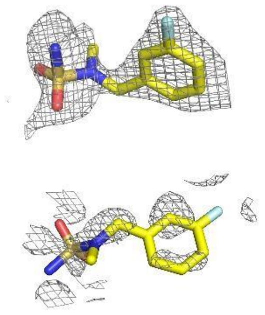 1.62 Å	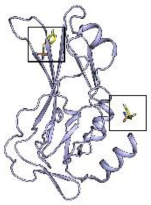 Pocket C & Pocket D
5QSD	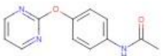 Z54571979	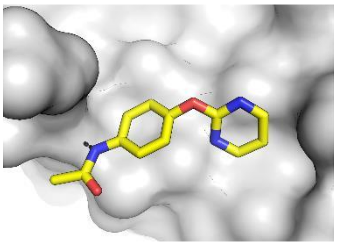	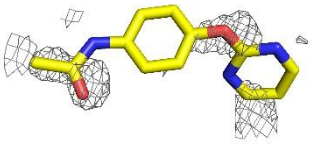 1.87 Å	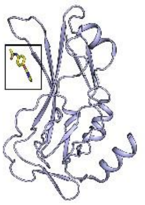 Pocket A’
5QSE	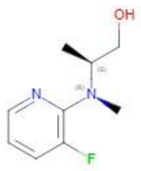 Z2017168803	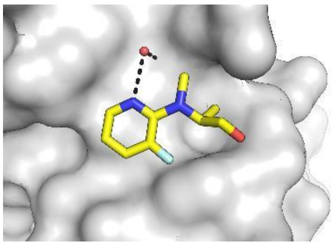	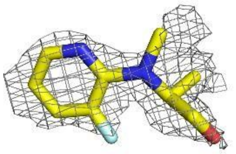 2.01 Å	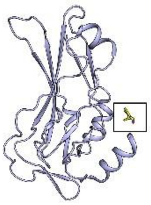 Pocket C
5QSF	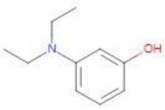 Z2856434814	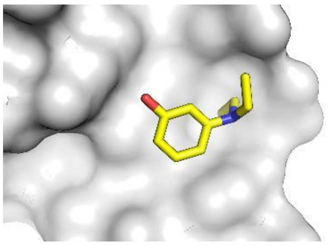	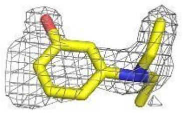 1.96 Å	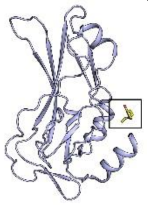 Pocket C

PDB	Ligand	Pocket	2Fo-Fc map (1σ)	Location
5QSG	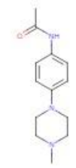 Z2856434903	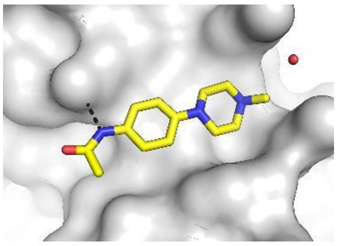	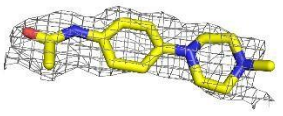 1.87 Å	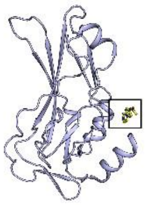 Pocket C
5QSH	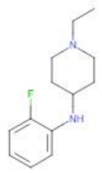 Z2856434868	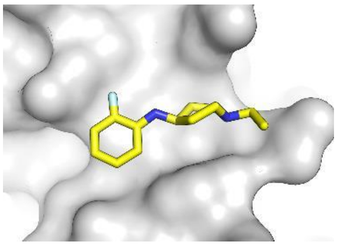	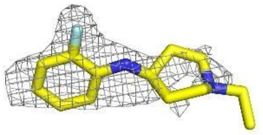 1.90 Å	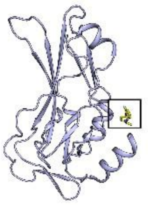 Pocket C
5QSI	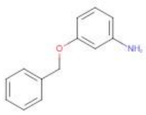 Z933326822	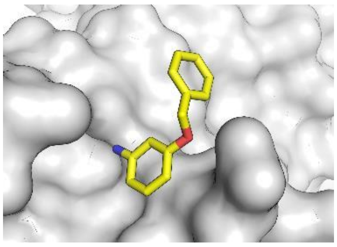	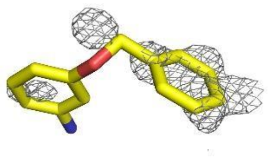 1.64 Å	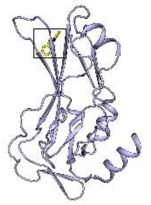 Pocket D
5QSJ	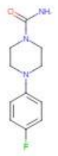 Z198194394	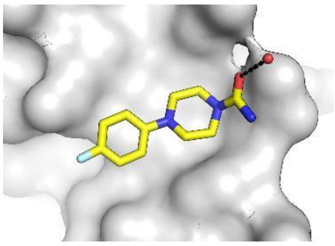	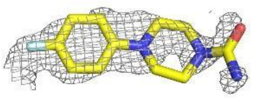 1.49 Å	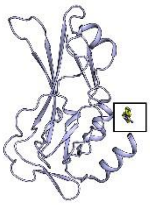 Pocket C
5QSK	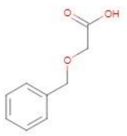 Z2856434906	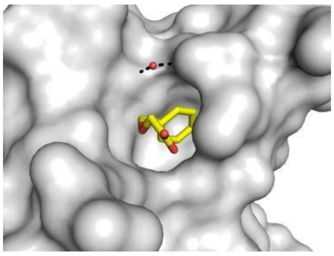	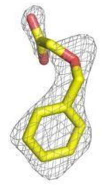 1.55 Å	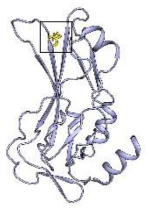 Pocket D
5QSL	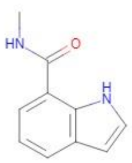 Z1273312153	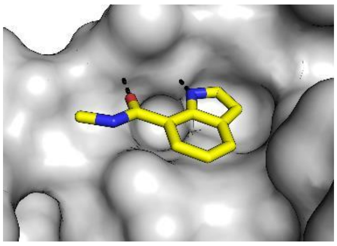	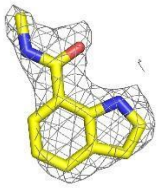 2.20 Å	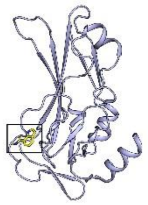 Pocket B

PDB	Ligand	Pocket	2Fo-Fc map (1σ)	Location
5QRF	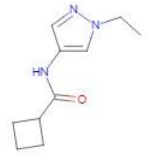 Z373768900	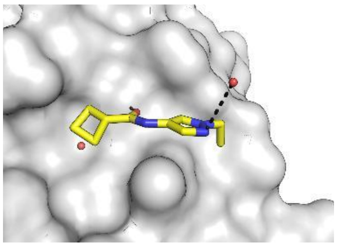	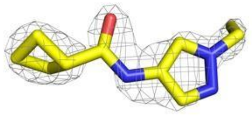 2.03 Å	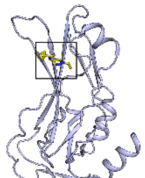 Pocket A
5QRG	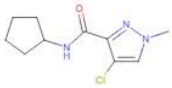 Z275151340	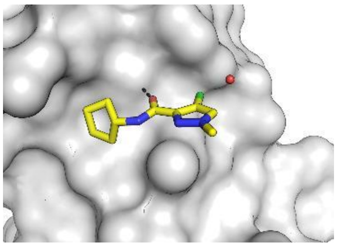	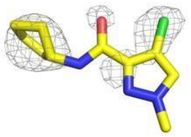 1.95 Å	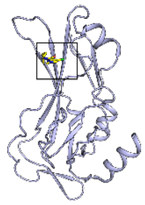 Pocket A
5QRH	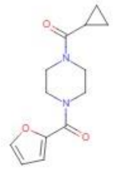 Z32327641	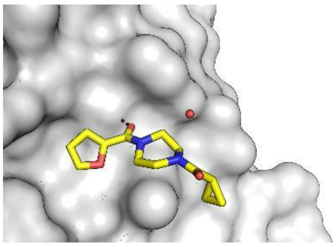	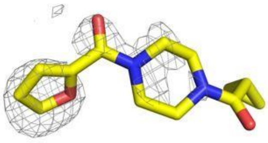 1.81 Å	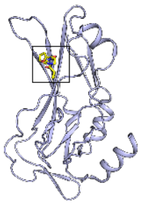 Pocket A
5QRI	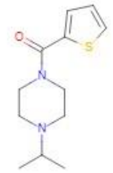 Z2856434826	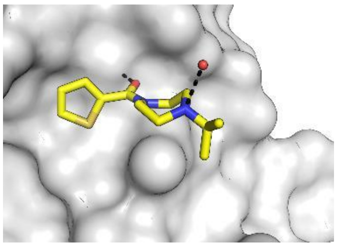	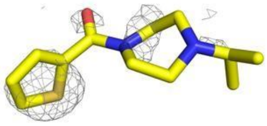 1.83 Å	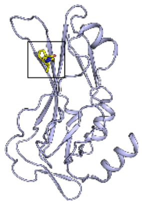 Pocket A
5QRJ	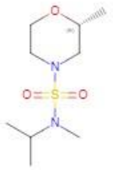 Z416341642	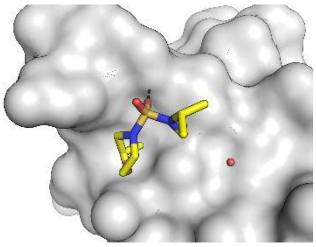	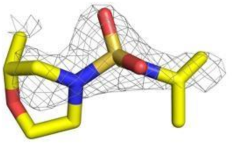 1.81 Å	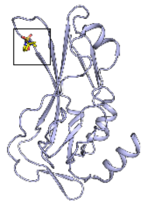 Pocket A
5QRK	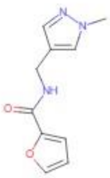 Z275179758	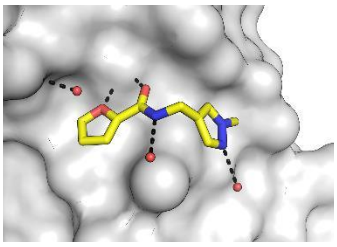	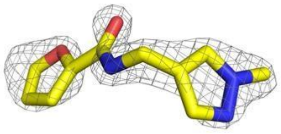 1.63 Å	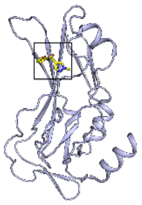 Pocket A

PDB	Ligand	Pocket	2Fo-Fc map (1σ)	Location
5QRL	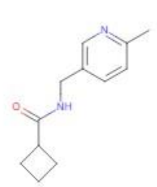 Z437516460	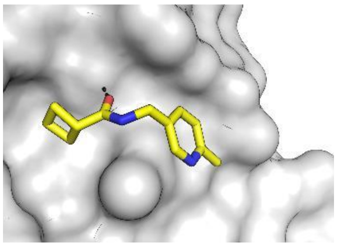	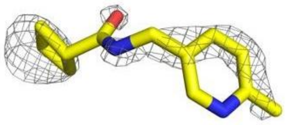 1.76 Å	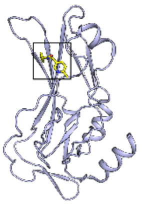 Pocket A
5QRM	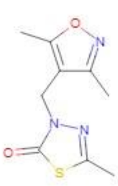 Z1899842917	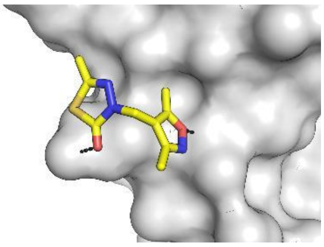	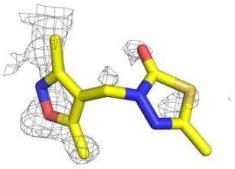 1.55 Å	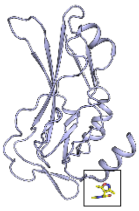 DNA Interface
5QRN	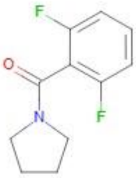 Z54226006	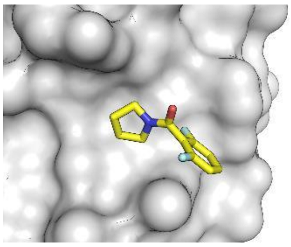	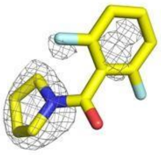 1.62 Å	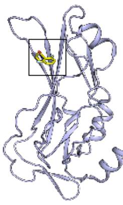 Pocket A
5QRO	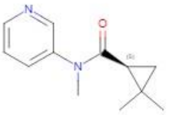 Z1506050651	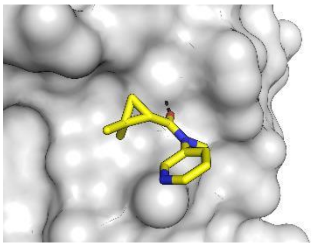	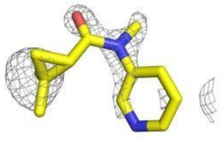 1.61 Å	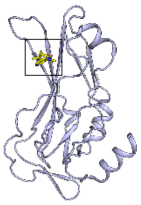 Pocket A
5QRP	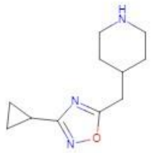 Z2442270563	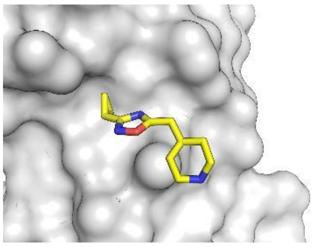	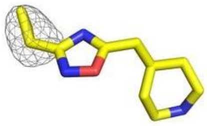 1.67 Å	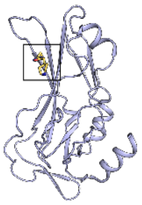 Pocket A
5QRQ	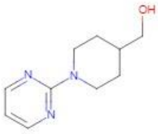 Z645232558	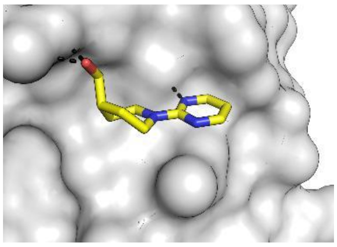	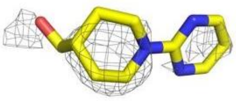 2.10 Å	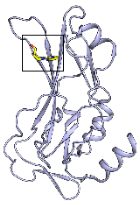 Pocket A

PDB	Ligand	Pocket	2Fo-Fc map (1σ)	Location
5QRR	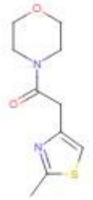 Z31720228	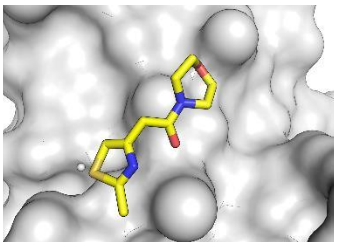	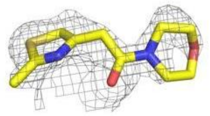 1.69 Å	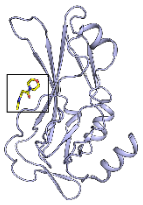 Pocket Other
5QRS	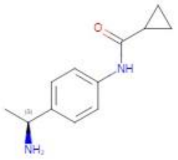 Z1891773393	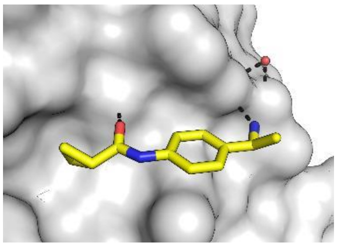	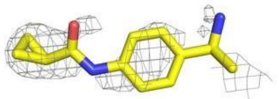 2.06 Å	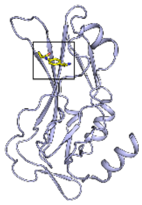 Pocket A
5QRT	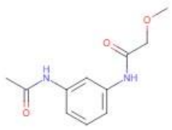 Z31735562	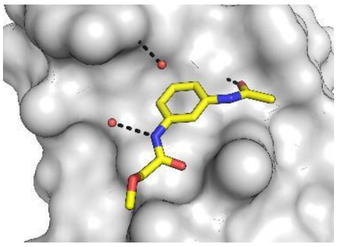	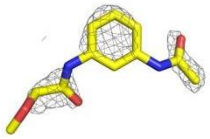 1.77 Å	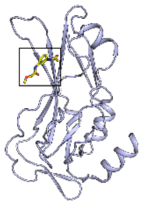 Pocket A
5QRU	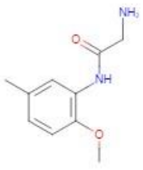 Z235341991	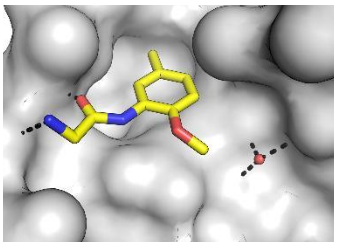	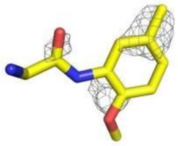 1.76 Å	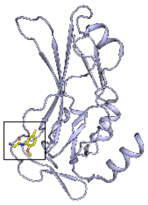 Pocket B
5QRV	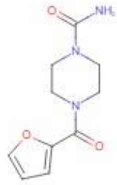 Z198194396	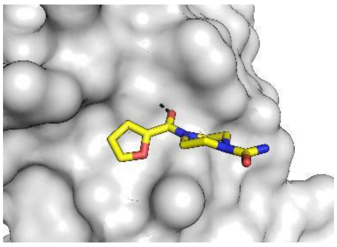	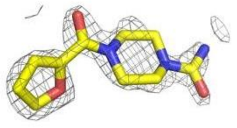 1.67 Å	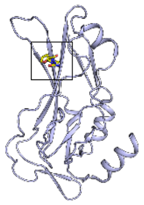 Pocket A
5QRW	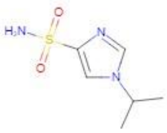 Z1509882419	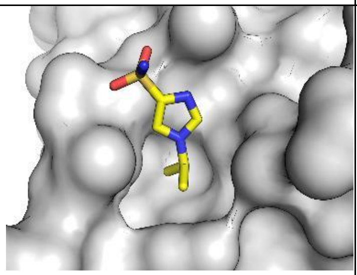	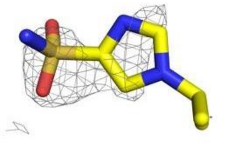	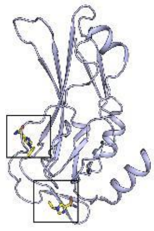

PDB	Ligand	Pocket	2Fo-Fc map (1σ)	Location
		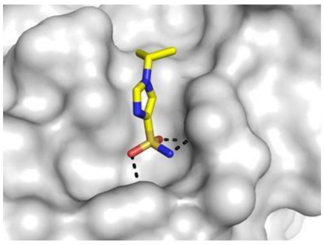	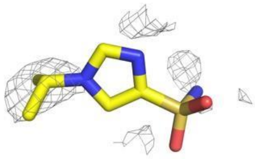 1.74 Å	Pocket B & DNA interface
5QRX	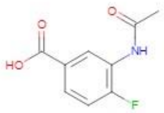 Z364328788	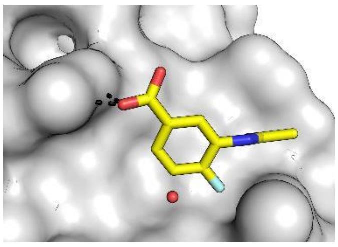	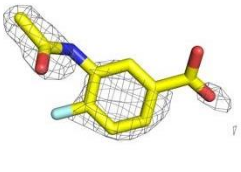 1.87 Å	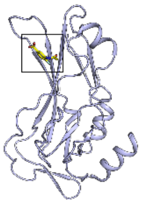 Pocket A
5QRY	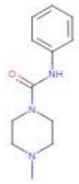 Z2856434890	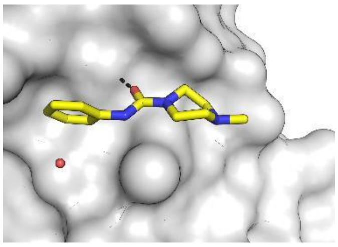	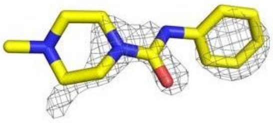 1.58 Å	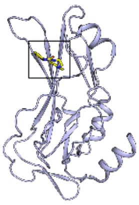 Pocket A
5QRZ	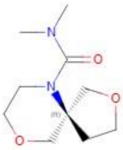 Z1998104358	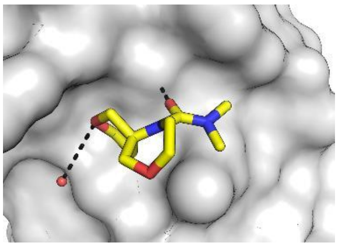	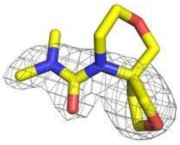 1.96 Å	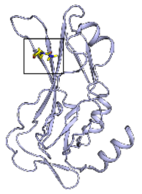 Pocket A
5QS0	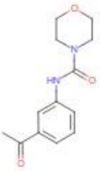 Z274555794	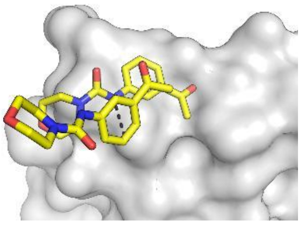	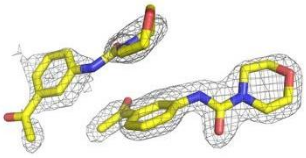 1.60 Å	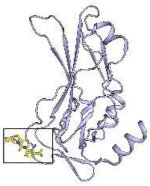 Other & Other
5QS1	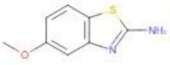 Z1954800564	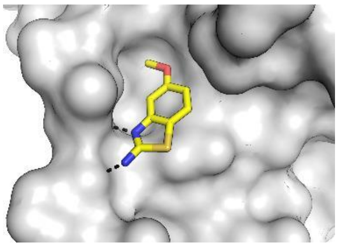	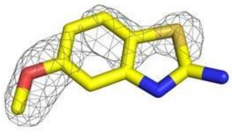 1.66 Å	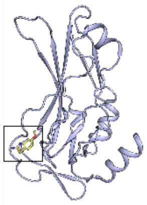 Pocket B

PDB	Ligand	Pocket	2Fo-Fc map (1σ)	Location
5QS2	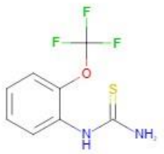 Z291279160	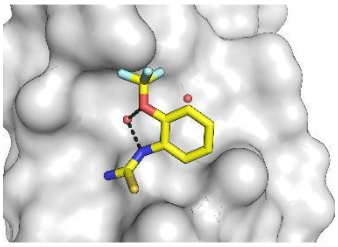	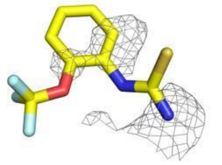 1.68 Å	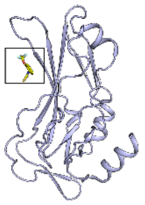 Pocket A
5QS3	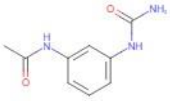 Z198195770	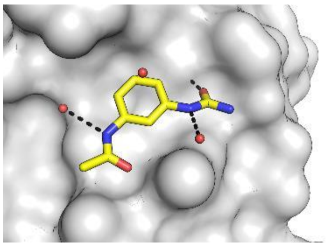	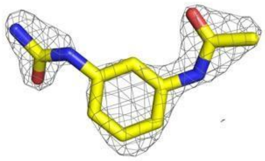 1.71 Å	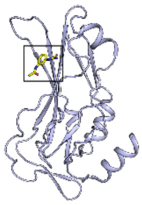 Pocket A
5QS4	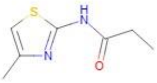 Z30820160	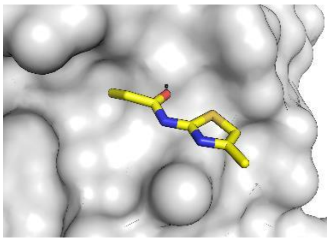	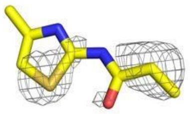 1.65 Å	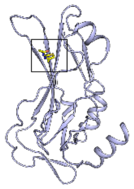 Pocket A
5QS5	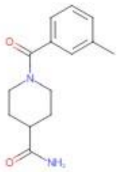 Z32400357	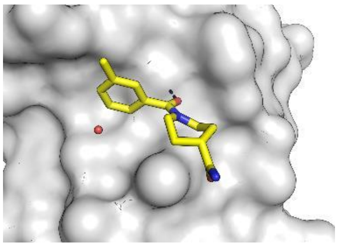	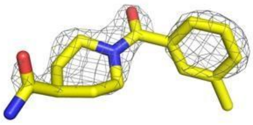 1.81 Å	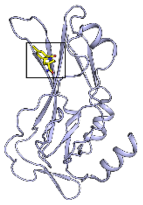 Pocket A

Supplementary Table 2 – Overview of the brachyury fragment screening hits. Each PDB entry is listed together with the chemical structure of the fragment, the fragment binding pocket, location, and electron density map. Maps shown are 2Fo-1Fc type calculated with the final refined coordinates and are shown to allow comparison of the relative strengths of the electron density for each fragment hit rather than the experimental evidence for fragment binding which is based on the PANDDA event maps.

Entry	Structure	KD µM	Curve fit	Sensogram
1a*	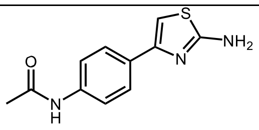<br>CSC000256427	–	–	–
1b*	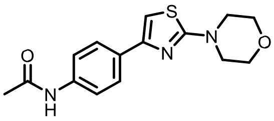<br>CSC02789850	316 ±<br>90	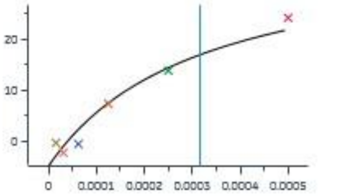	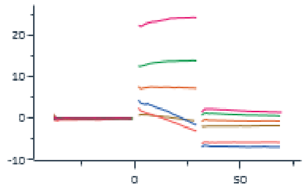
2 2–<br>071	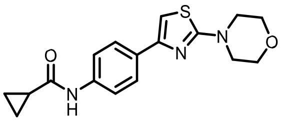	19 ±<br>14	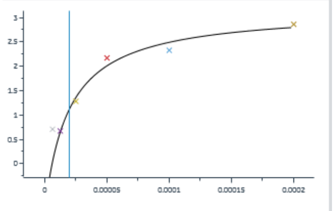	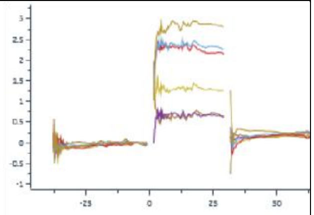
3<br>166–3	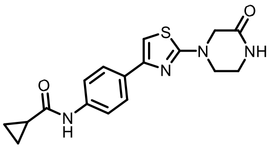	14 ±<br>9	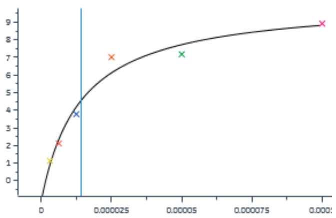	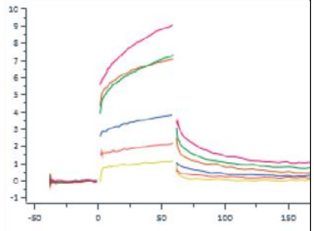
4<br>194–<br>12	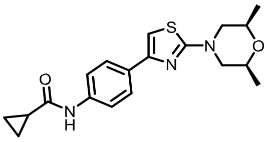	21±<br>10	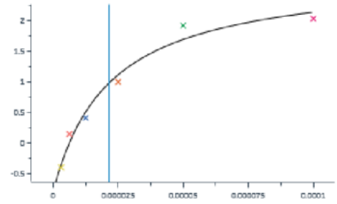	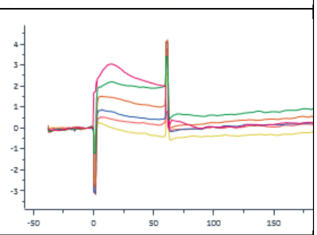
5<br>0290A	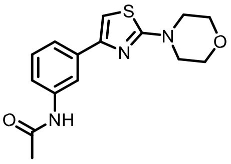	84 ±<br>9	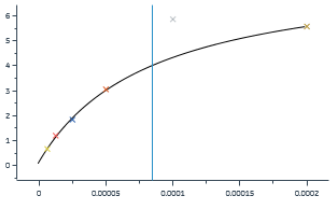	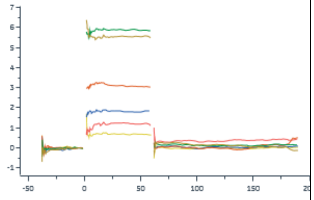
6<br>3007H	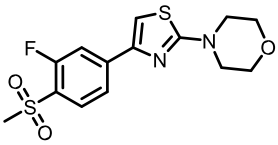	25 ± 7	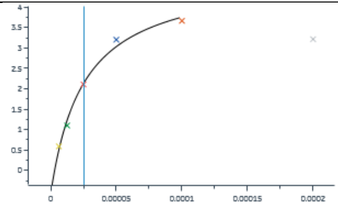	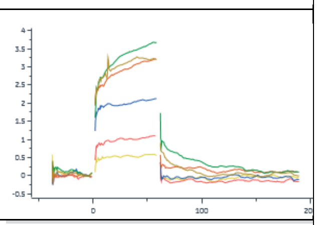
7	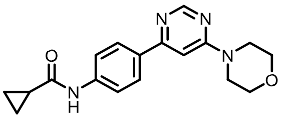	14 ± 9	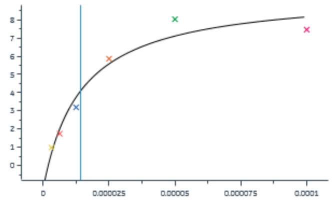	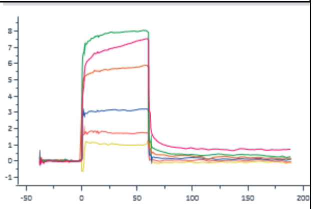

8	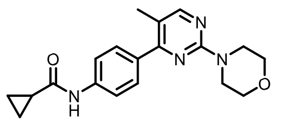	16 ± 1	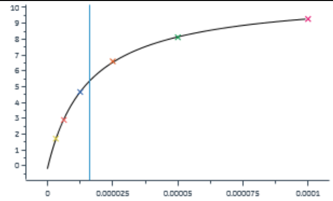	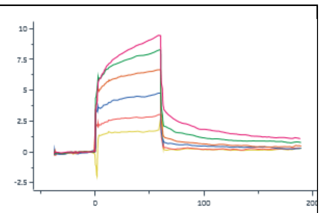
9	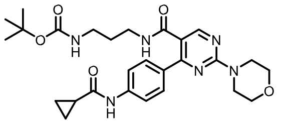	30 ± 9	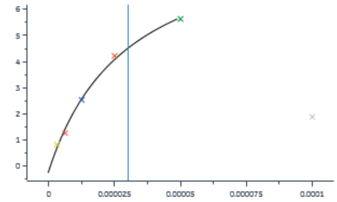	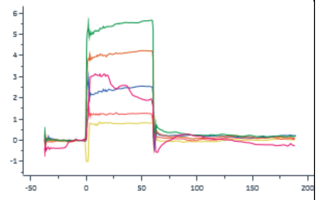
10	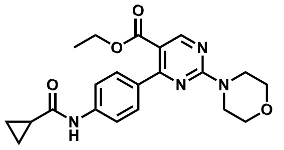	8 ± 3	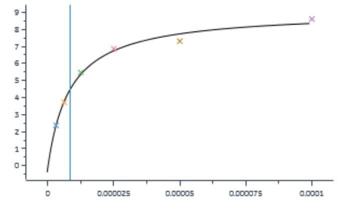	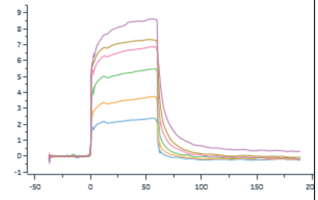
11	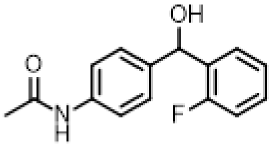	340 ± 8	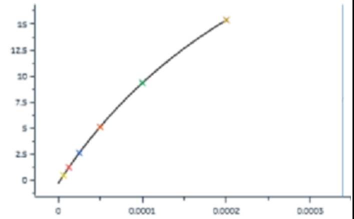	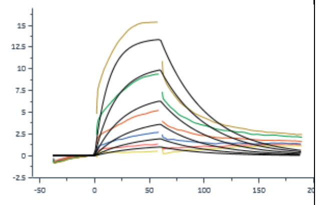
12	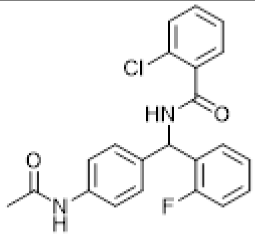	49 ± 6	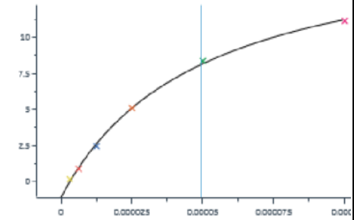	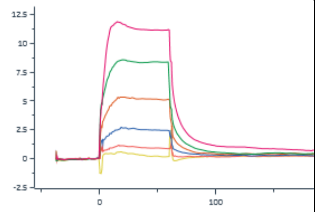
13	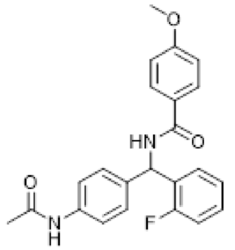	17 ± 9	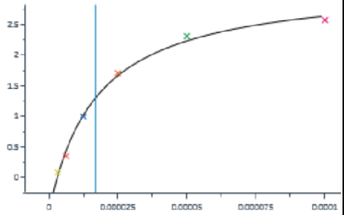	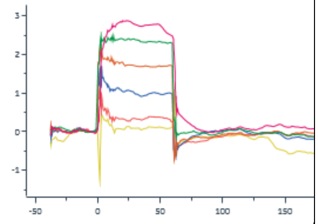

Supplementary Table 3 – Summary of compounds binding to brachyury based on elaboration of fragment hits targeting pocket A'. SPR dose response fits are shown in the 4th column with response in RU as the Y axis and molar compound concentration as the X axis. The vertical line depicts the apparent dissociation constant using a dose response fit. Sensograms are shown in the 5th column

plotted as response in RU over time in seconds. Errors are shown as ± the standard error based on the affinity fit to the concentration response curves.

Supplementary Methods

General Chemistry Information

All reagents and solvents, unless specifically stated, were used as obtained from their commercial sources without further purification. Air and moisture sensitive reactions were performed under an inert atmosphere using nitrogen or argon in a previously oven-dried or flame-dried reaction flask, and addition of reagents were done using a syringe. All microwave (MW) reactions were carried out in a Biotage Initiator EXP US 400W microwave synthesizer. Thin layer chromatography (TLC) analyses were performed using 200 μm precoated sorbtech fluorescent TLC plates and spots were visualized using UV light. High resolution mass spectrometry samples were analyzed with a ThermoFisher Q Exactive HF-X (ThermoFisher, Bremen, Germany) mass spectrometer coupled with a H2Os Acquity H-class liquid chromatograph system. Column chromatography was undertaken with a Biotage Isolera One instrument. HPLC: Phenomenex Luna Phenyl-Hexyl (5 μm particle size, 100 Å pore size, 75 x 30 mm) column on an Agilent 1100 Series instrument equipped with an Agilent G1315B diode array detector measured at 220/254 nm. Nuclear magnetic resonance (NMR) spectrometry was run on a varian Inova 400 MHz or Bruker Avance III 700 MHz spectrometer equipped with a TCI HC/N-D 5 mm cryoprobe and data was processed using the MestReNova processor. Chemical shifts are reported in ppm with residual solvent peaks referenced as internal standard.

Synthesis of *N*-(4-(2-morpholinothiazol-4-yl)phenyl)cyclopropanecarboxamide (2)

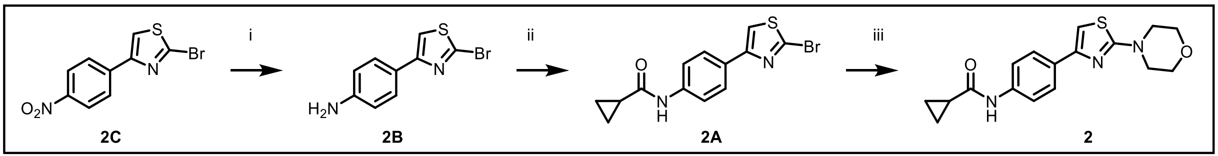

Supplementary Figure 11 – Synthesis of compound **2** : i. hydrazine hydrate, EtOH, Raney N (excess), 50 °C, 2 hr, 86%; ii. cyclopropyl acyl chloride, pyridine, 0 °C, 24 hr, 54%; iii. Morpholine, DMA, 140 °C, 16 h, 65%.

Synthesis of 4-(2-bromothiazol-4-yl)aniline (2B): To a suspension of 2-bromo-4-(4-nitrophenyl)thiazole (400 mg, 1.00 Eq, 1.40 mmol) in EtOH (30 mL) was added hydrazinehydrate (1.05 g, 1.02 mL, 15.00 Eq, 21.0 mmol) and the reaction mixture was heated to 50 °C for 15 min until the starting material was completely dissolved. Excess Raney Nickel (823 mg, 112 µL, 10.00 Eq, 14.0 mmol) was added to the reaction mixture and the reaction was maintained at 50 °C. When gas evolution ceased the reaction mixture was filtered through celite and concentrated to dryness to yield the desired product **2B** (310 mg, 86%) which was taken to the next step with no further purifications.

^{1}H NMR (850 MHz, DMSO) δ 7.71 (s, 1H), 7.59 – 7.55 (m, 2H), 6.62 – 6.58 (m, 2H), 5.38 (s, 2H).

^{13}C NMR (214 MHz, DMSO) δ 158.8, 152.48, 138.3, 130.3, 124.1, 116.9, 116.6.

Synthesis of N-(4-(2-bromothiazol-4-yl)phenyl)cyclopropanecarboxamide (2A): To a 100 mL RBF was added 4-(2-bromothiazol-4-yl)aniline (350 mg, 1.0 eq, 1.37 mmol) and 20 mL of DCM. The reaction mixture was cooled to 0 °C using an ice bath and pyridine (217 mg, 2.74 mmol, 2.00 eq.) was

added to the reaction mixture followed by cyclopropyl acyl chloride (172 mg, 1.65 mmol, 1.20 eq) dropwise over 30 min. The reaction mixture was stirred for 3.0-24 h at room temperature. The reaction mixture was poured into a separatory funnel and washed 3 x 10.0 mL of saturated $NaHCO_3$(aq) and 15.0 mL brine. The organic layer was dried over anhydrous Na_2SO_4 filtered and evaporated. The crude material waspurified by column chromatography on silica gel (30-50% Ethyl acetate in hexanes) to afford the product **2A** (240 mg, yield 54%, 97% pure).

^{1}H NMR (700 MHz, DMSO) δ 10.26 (s, 1H), 7.95 (s, 1H), 7.79 – 7.75 (m, 2H), 7.61 (d, J = 8.7 Hz, 2H), 1.73 (tt, J = 7.4, 4.9 Hz, 1H), 0.75 (ddd, J = 9.1, 6.1, 2.3 Hz, 4H).

^{13}C NMR (176 MHz, DMSO) δ 172.2, 154.8, 140.1, 136.3, 128.1, 126.9, 119.5, 117.6, 60.2, 15.1, 14.6, 7.8.

Synthesis of N-(4-(2-morpholinothiazol-4-yl)phenyl)cyclopropanecarboxamide (2): To a small 4 mL dram vial was added N-(4-(2-bromothiazol-4-yl)phenyl)cyclopropanecarboxamide **2** (30 mg, 1 Eq, 93 µmol), to this was added morpholine (16 mg, 2 eq. 190 µmol) and DMA (0.5 mL). The reaction mixture was then heated to 140 °C overnight. After heating for 16 h the reaction mixture was taken to dryness and purified by HPLC 0.05% TFA in H_2O/MeOH. Product fractions were collected and dried to yield the product **2** (20 mg, yield 65%, 98% pure), m/z: 330.12722 [M+H]+.

H NMR (700 MHz, DMSO) δ 10.25 (s, 1H), 7.80 – 7.76 (m, 2H), 7.61 (d, J = 8.3 Hz, 2H), 7.19 (d, J = 1.3 Hz, 1H), 3.74 (t, J = 4.9 Hz, 4H), 3.44 (t, J = 4.8 Hz, 4H), 1.79 (s, 1H), 0.83 – 0.77 (m, 4H).

^{13}C NMR (101 MHz, cdcl$_3$) δ 129.1, 127.2, 126.7, 102.6, 77.3, 77.0, 76.7, 66.2, 16.2, 14.8, 11.1, 11.0, 8.0, 7.4.

Synthesis of N-(4-(2-(3-oxopiperazin-1-yl)thiazol-4-yl)phenyl)cyclopropanecarboxamide (3): To a small 4 mL dram vial was added N-(4-(2-bromothiazol-4yl)phenyl)cyclopropanecarboxamide, **2A** (30 mg, 1 eq., 93 μmol), to this was added piperazin-2-one (28 mg, 3 eq., 280 μmol) and DMA (0.5 mL). The reaction mixture was then heated to 140 °C overnight. After heating for 16 h the reaction mixture was taken to dryness and purified by HPLC 0.05% TFA in H_2O/MeOH, product fractions were collected and dried to yield the product **3** (9.0 mg, yield 28%, 97% pure), m/z: 343.112 $[M+H]^+$.

^1H NMR (850 MHz, DMSO) δ 10.25 (s, 1H), 8.19 (t, J = 2.8 Hz, 1H), 7.81 – 7.77 (m, 2H), 7.64 – 7.59 (m, 2H), 7.19 (s, 1H), 4.01 (s, 2H), 3.70 – 3.66 (m, 2H), 3.37 (ddd, J = 7.0, 5.2, 2.9 Hz, 2H), 1.79 (tt, J = 7.8, 4.8 Hz, 1H), 0.80 (ddt, J = 12.8, 10.1, 4.0 Hz, 4H).

^{13}C NMR (214 MHz, DMSO) δ 174.7, 171.8, 169.1, 153.6, 142.0, 132.5, 129.3, 122.0, 104.6, 53.9, 40.6, 37.6, 24.5, 17.7, 10.30.

Synthesis of N-(4-(2-((2R,6S)-2,6-dimethylmorpholino)thiazol-4-yl)phenyl)cyclopropanecarboxamide (4): To a small 4 mL dram vial was added N-(4-(2-bromothiazol-4-yl)phenyl)cyclopropanecarboxamide (30 mg, 1 Eq, 93 μmol), to this was added (2S,6R)-2,6-dimethylmorpholine (32 mg, 3 Eq. 280 μmol) and DMA (0.5 mL). The reaction mixture was then heated to 140 °C overnight. After heating for 16 h the reaction mixture was taken to dryness and purified by HPLC 0.05% TFA in H_2O/MeOH, product fractions were collected and dried to yield the product **4** (10.3 mg, yield 31%, 96% pure), m/z: 358.1584 [M+H]+.

^1H NMR (850 MHz, DMSO) δ 10.25 (s, 1H), 7.78 – 7.76 (m, 2H), 7.64 – 7.59 (m, 2H), 7.14 (s, 1H), 4.08 (pd, J = 6.4, 3.5 Hz, 2H), 3.55 (dd, J = 12.3, 3.5 Hz, 2H), 3.16 (dd, J = 12.3, 6.1 Hz, 2H), 1.20 (d, J = 6.5 Hz, 7H), 0.84 – 0.76 (m, 4H), 1.79 (tt, J = 7.7, 4.7 Hz, 1H).

^{13}C NMR (214 MHz, DMSO) δ 174.7, 174.1, 153.5, 141.9, 132.6, 129.3, 122.0, 104.1, 68.1, 55.8, 20.69, 17.68, 10.29.

Synthesis of N-(3-(2-morpholinothiazol-4-yl)phenyl)acetamide (5):

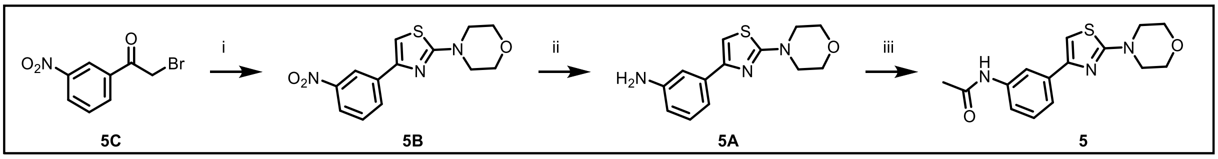

Supplementary Figure 12 - Synthesis of compound **5** : i. thiourea, EtOH, reflux, 3 hr; ii. hydrazine hydrate, EtOH, Raney N (excess), 50 °C, 2 hr, 89%; iii. acetyl chloride, pyridine, THF, 0 °C to rt 2 hr then rt overnight, 82%.

Synthesis of 4-(4-(3-nitrophenyl)thiazol-2-yl)morpholine (5B): To a 100 mL RBF was added thiourea (1.52 g, 20.0 mmol) and 2-bromo 4′-bromoacetophenone (5.56 g, 20.0 mmol) in EtOH (40 mL). The suspension was refluxed for 3 h. After filtration, the precipitate was washed with EtOH to get [4-(4-bromophenyl)-1,3-thiazol-2-yl]amino hydrobromide (1:1) as a solid, **5B** (6.05 g, yield 90%) which was used for the next step without purification. ^{1}H NMR (850 MHz, DMSO) δ 8.63 (q, J = 2.3 Hz, 1H), 8.29 (dt, J = 7.7, 1.3 Hz, 1H), 8.13 (ddd, J = 8.1, 2.4, 1.0 Hz, 1H), 7.68 (t, J = 8.0 Hz, 1H), 7.62 (s, 1H), 3.77 – 3.73 (m, 4H), 3.48 – 3.45 (m, 4H).

^{13}C NMR (214 MHz, DMSO) δ 174.16, 151.09, 139.28, 135.04, 133.68, 125.28, 122.92, 108.55, 68.45, 50.98, 43.14.

Synthesis of 3-(2-morpholinothiazol-4-yl)aniline (5A): In a 100 mL RBF was added 4-(4-(3-nitrophenyl)thiazol-2-yl)morpholine (2.5 g, 8.6 mmol, 1 eq.) in EtOH (50 mL) was added hydrazinehydrate (6.4 g, 6.3 mL, 15.00 Eq, 130 mmol) and the reaction mixture was heated to 50 °C for 15 min until the starting material dissolved completely. Excess of Raney Nickel (10 g, 20.00 Eq, 170 mmol) was added to the reaction mixture and the reaction was maintained at 50 °C. When gas evolution ceased, the reaction mixture was was filtered over celite and concentrated to give 3-(2morpholinothiazol-4-yl)aniline, **5A** (2.0 g, yield 89%, 96% purity) The product was taken to the next step with no further purifications.

Synthesis of N-(3-(2-morpholinothiazol-4-yl)phenyl)acetamide (5): To a 100 mL RBF was added 3-(2-morpholinothiazol-4-yl)aniline (65.3 mg, 0.250 mmol, 1 eq.) in 5 mL of THF. The reaction mixture was cooled to 0 °C using an ice bath. To this was added pyridine (39.6 mg, 40.5 µL, 0.5 mmol, 2 eq.) followed by dropwise addition of acetyl chloride (23.5 mg, 0.30 mmol, 1.20 eq.) over 10 min. The reaction mixture was stirred at room temperature until complete by TLC. The reaction mixture was poured into a separatory funnel, washed 3 x 10.0 mL of saturated $NaHCO_3$(aq) and 15.0 mL brine. The organic layer was dried over Na_2SO_4, filtered and concentrated in vacuo. The crude product was purified by silica column chromatography (30%-50% Ethyl acetate in hexanes) to afford the product N-(3-(2-morpholinothiazol-4yl)phenyl)acetamide, **5** (62 mg, yield 82%, 96% purity), m/Z: 304.11139 $[M+H]^+$.

^1H NMR (400 MHz, CD_3OD) δ 8.01 (t, J = 1.9 Hz, 1H), 7.55 (dt, J = 7.9, 1.4 Hz, 1H), 7.48 (ddd, J = 8.1, 2.2, 1.1 Hz, 1H), 7.29 (t, J = 7.9 Hz, 1H), 6.99 (s, 1H), 3.84 – 3.77 (m, 4H), 3.52 – 3.46 (m, 4H), 2.12 (s, 3H).

^{13}C NMR (101 MHz, CD_3OD) δ 128.47, 121.41, 118.99, 117.48, 102.02, 65.80,
22.41.

Synthesis of 4-(4-(3-fluoro-4-(methylsulfonyl)phenyl)thiazol-2-yl)morpholine (6): To a 4 mL microwave vial was added 4-(4-bromothiazol-2-yl)morpholine (50.00 mg, 1.00 Eq, 200.7 µmol), 3-Fluoro-4-(methylsulfonyl)phenylboronic acid (56.88 mg, 1.30 Eq, 260.9 µmol) and tetrakis (11.60 mg, 0.05 Eq, 10.03 µmol) ($Pd(P(Ph)_3)_4$ (5 mol%)] were dissolved in a mixed solution of 1,4-dioxane (3.0 mL)/H_2O (0.3 mL) under argon. After degassing for 5 minutes, Cs_2CO_3 (98.09 mg, 1.50 Eq, 301.0 µmol) was added and the reaction mixture was heated in a microwave at 120 °C for 30 min.

The reaction mixture was allowed to cool to room temperature and reaction mixture was concentrated in vacuo. To the reaction mixture H2O was added and the solution was acidified to pH=2 using 2N HCl. The white solid precipitate was collected by filtration and dried to give the desired compound 4-(4-(3-fluoro-4-(methylsulfonyl)phenyl)thiazol-2-yl)morpholine, **6** (52.0 mg, yield 75.67%, purity >95%), m/Z: 343.05826 $[M+H]^+$.

^{1}H NMR (700 MHz, DMSO) δ 7.98 – 7.91 (m, 2H), 7.88 (t, J = 7.7 Hz, 1H), 7.72 (d, J = 1.3 Hz, 1H), 3.75 (t, J = 4.8 Hz, 4H), 3.48 (t, J = 4.8 Hz, 4H), 3.34 (s, 3H).

^{13}C NMR (214 MHz, CDCl3) δ 173.96, 163.06, 161.88, 151.23, 145.26, 145.22, 132.53, 129.30, 129.23, 124.41, 124.39, 117.24, 117.13, 108.33, 68.79, 68.68, 51.28, 50.61, 46.68, 46.66.

Synthesis of N-(4-(6-morpholinopyrimidin-4-yl)phenyl)cyclopropanecarboxamide (7):

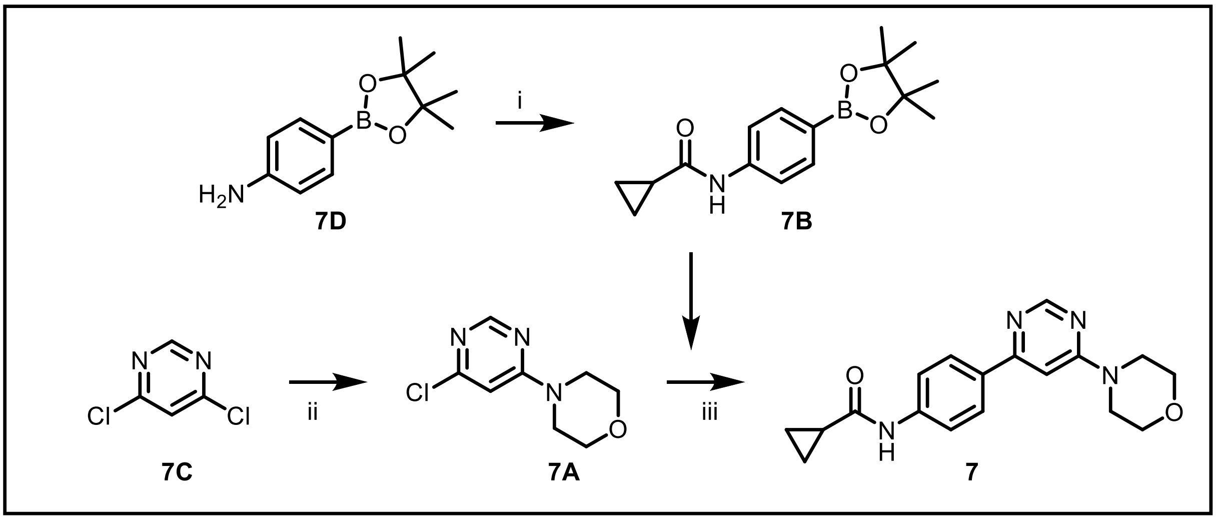

Supplementary Figure 13 - Synthesis of compound **7** : i. HATU, DIPEA, DMF, RT, 2 hr, 44%; ii. DIPEA, CAN, RT, 12 hr, 42%; iii. $PdCl_2(PPh_3)_2$, K_2CO_3, dioxane, water, 90 °C, 12 hr, 9%.

Synthesis of N-(4-(4,4,5,5-tetramethyl-1,3,2-dioxaborolan-2-yl)phenyl)cyclopropanecarboxamide (7B): To a stirred solution of cyclopropane carboxylic acid (0.3 g, 3.48 mmol, 1.0 eq.) in DMF (2.0 mL) was

added HATU (1.81 g, 5.23 mmol, 1.5 eq.) followed by addition of DIPEA (0.67 g, 5.23 mmol, 1.5 eq.) and stirred at room temperature for 10 min. To this reaction mixture was added 4-(4,4,5,5tetramethyl-1,3,2-dioxaborolan-2-yl)aniline (**7D**) (0.7 g, 3.48 mmol, 1.0 eq.) and stirred at room temperature for 2 h. Reaction progress was monitored by TLC, after completion, the reaction mixture was diluted with H_2O (20 mL) and extracted with EtOAc (3 x 30 mL). The organic layer was washed with brine solution, dried over Na_2SO_4, filtered and concentrated to give crude compound which was purified by flash column chromatography using a 5-10% EtOAc/n-hexane. The product fractions were collected and dried to yield N-(4-(4,4,5,5-tetramethyl-1,3,2-dioxaborolan-2-yl)phenyl)cyclopropanecarboxamide (**7B**) (0.4 g, 44%) as an off white solid. m/z: 288.19 $[M+H]^+$.

^1H NMR (400 MHz, DMSO-d6) δ 10.32 (s, 1H), 7.60 (s, 4H), 1.83 – 1.76 (m, 1H), 1.28 (s, 12H), 0.80 (d, J = 5.0 Hz, 4H).

Synthesis of 4-(6-chloropyrimidin-4-yl)morpholine (7A): To a stirred solution of 4, 6-dichloropyrimidine (**7C**) 0.5 g (3.37 mmol, 1.0 eq.) in MeCN (10 mL) was added morpholine 0.32 g (3.71 mmol, 1.1 eq.), followed by the addition of DIPEA (5.19 g, 5.05 mmol, 1.5 eq.). and stirred at room temperature for 12 h. Reaction progress was monitored by TLC, after completion, the mixture was diluted with H_2O (10 mL), solid formed was filtered and dried to give 4-(6-chloropyrimidin-4-yl) morpholine (**7A**) (0.28 g, 42%) as a white solid. m/z 200.03 $[M+H]^+$.

^1H NMR (400 MHz, DMSO-d6) δ 8.37 (s, 1H), 6.98 (s, 1H), 3.40-3.90 (m, 8H).

Synthesis of N-(4-(6-morpholinopyrimidin-4-yl)phenyl)cyclopropanecarboxamide (7): To a stirred solution of 4-(6-

chloropyrimidin-4-yl)morpholine (**7A**) (0.25 g, 1.25 mmol, 1 eq.) in 1,4-dioxane (5.0 mL) was added N-(4-(4,4,5,5tetramethyl-1,3,2-dioxaborolan-2-yl) phenyl)cyclopropanecarboxamide (**7B**) (0.43 g, 1.50 mmol, 1.2 eq.). To this mixture was added K_2CO_3 (0.69 g, 1.87 mmol, 1.5 eq.) in H_2O (2.5 mL). The reaction mixture was degassed with nitrogen for 20 min and $(PPh_3)_2PdCl_2$ (0.051 g, 0.06 mmol, 0.05 eq.) was added. The reaction mixture was heated at 90 ^{o}C for 12 h. Reaction progress was monitored by TLC and LCMS, after completion, the mixture was diluted with H_2O (10 mL) and extracted with EtOAc (3 x 20 mL). The organic layer was washed with brine solution, dried over Na_2SO_4, filtered and concentrated to give crude compound. The resulting crude was purified by prep. HPLC using 0.1% formic acid in H_2O/MeCN. The product fractions were collected and concentrated to give N-(4-(6-morpholinopyrimidin-4-yl)phenyl)cyclopropane carbox-amide, **7** (0.012 g, 9%) as a white solid. m/z $[M+H]^+$ 325.20.

^{1}H NMR (400 MHz, DMSO-d6) δ 10.41 (s, 1H), 8.56 (s, 1H), 8.13 (d, J = 8.6 Hz, 2H), 7.71 (d, J = 8.6 Hz, 2H), 7.28 (s, 1H), 3.59- 3.79 (m, 8H), 1.85 – 1.77 (m, 1H), 0.86 – 0.80 (m, 4H).

^{13}C NMR (176 MHz, DMSO) δ 172.37, 162.96, 161.73, 158.36, 141.71, 132.01, 127.91, 119.07, 97.95, 66.33, 44.39, 15.13, 7.85

Synthesis of N-(4-(5-methyl-2-morpholinopyrimidin-4-yl)phenyl)cyclopropanecarboxamide (8):

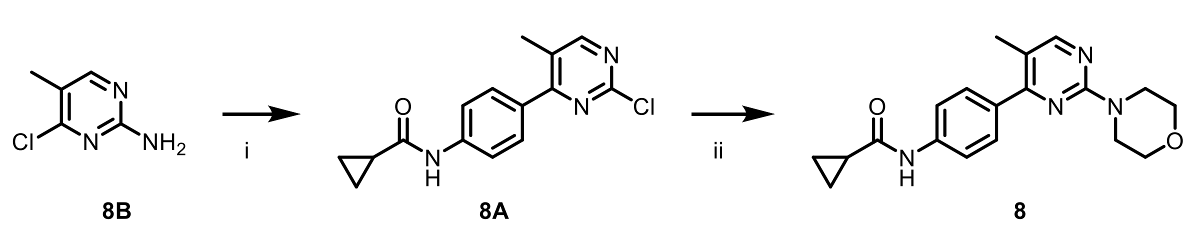

Supplementary Figure 14 - Synthesis of compound **8** : i. $PdCl_2(PPh_3)_2$, K_2CO_3, dioxane, water, 90 °C, 12 hr, 59%; ii. DIPEA, DMF, 80 °C, 5 hr, 17%.

Synthesis of N-(4-(2-chloro-5-methylpyrimidin-4-yl)phenyl)cyclopropane carboxamide (8A): To a stirred solution of 2,4-dichloro-5-methylpyrimidine (**8B**) (0.25 g, 1.53 mmol, 1.0 eq.) in 1,4-dioxane (4.0 mL) was added N-(4-(4,4,5,5-tetramethyl1,3,2-dioxaborolan-2-yl)phenyl) cyclo-propanecarboxamide (**7B**) (0.52 g, 1.84 mmol, 1.1 eq.) and K_2CO_3 (0.31 g, 2.29 mmol, 1.5 eq.) in H_2O (4.0 mL) was added. The reaction mixture was degassed with nitrogen for 20 min and $(PPh_3)_2PdCl_2$ (0.1 g, 0.016 mmol, 0.1 eq.) was added. The reaction mixture was then heated at 90 °C for 12 h. Reaction progress was monitored by TLC and LCMS. After completion, the mixture was diluted with H_2O (10 mL) and extracted using EtOAc (3 x 20 mL). The organic layer was washed with brine solution, dried over Na2SO4, filtered and concentrated to give crude compound. The resulting crude was purified by flash column chromatography using 30 % EtOAc/n-hexane. The product fractions were collected and concentrated under reduced pressure to give N-(4-(2-chloro-5methylpyrimidin-4-yl)phenyl)cyclopropane-carboxamide (**8A**) (0.25 g, 59%) as an off white solid. m/z: 288.16 $[M+H]^+$

Synthesis of N-(4-(5-methyl-2-morpholinopyrimidin-4-yl)phenyl)cyclopropane carboxamide (8): To a stirred solution of N-(4-(2-chloro-5-methylpyrimidin-4-yl)phenyl)cyclopropane-carboxamide (**8A**)

(0.25 g, 0.87 mmol, 1.0 eq.) in DMF (5.0 mL) was added morpholine (0.12 g, 1.30 mmol, 1.5 eq.), followed by the addition of DIPEA (0.16 g, 1.30 mmol, 1.5 eq.). The reaction mixture was heated at 80 °C for 5 h. Reaction progress was monitored by TLC, after completion, the mixture was diluted with H_2O (20 mL) and extracted using EtOAc (3 x 30 mL). The organic layer was washed with brine solution, dried over Na_2SO_4, filtered and concentrated to give crude compound. The resulting crude was purified by HPLC using 0.1% formic acid in H_2O/MeCN. Product fractions were collected and lyophilized to give N-(4-(5-methyl-2morpholinopyrimidin-4-yl)phenyl) cyclopropane carboxamide (**8**) (0.05 g, 17%) as a white solid. m/z: 339.22 $[M+H]^+$

^1H NMR (400 MHz, DMSO-d6) δ 10.40 (s, 1H), 8.29 (s, 1H), 7.74 – 7.60 (m, 4H), 3.67 (s, 8H), 2.20 (s, 3H), 1.85 – 1.77 (m, 1H), 0.83 (d, J = 4.3 Hz, 4H).

^{13}C NMR (176 MHz, DMSO) δ 172.34, 164.06, 160.90, 160.25, 140.63, 133.36, 129.90, 118.76, 116.24, 66.47, 44.59, 16.51, 15.09, 7.80.

Synthesis of tert-butyl (3-(4-(4-(cyclopropanecarboxamido)phenyl)-2-morpholinopyrimidine-5-carboxamido)propyl)carbamate (9):

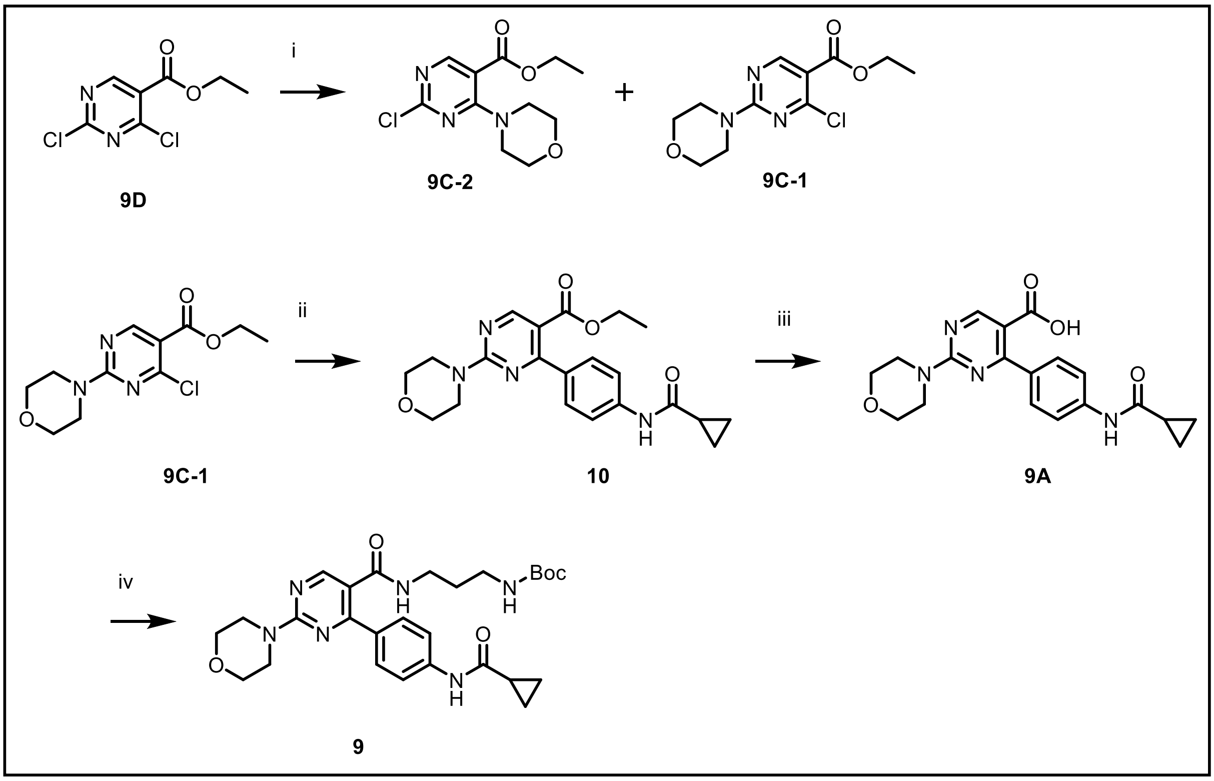

Supplementary Figure 15 -Synthesis of compound **9** : i. morpholine, DIPEA, NMP, RT, 3 hr, 12-19%; ii. $PdCl_2(PPh_3)_2$, K_2CO_3, dioxane, water, 90 °C, 12 hr, 8%; iii. NaOH, THF, MeOH, Water, RT, 12 hr, 45%; iv. EDC.HCl, HOBt, DIPEA, DMF, RT, 12 hr, 23%.

Synthesis of ethyl 2-chloro-4-morpholinopyrimidine-5-carboxylate (9C-2): To a stirred solution of ethyl 2,4-dichloropyrimidine-5-carboxylate (Int-1) (0.50 g, 2.26 mmol, 1.0 eq.) in NMP (5.0 mL) was added morpholine (0.18 g, 2.26 mmol, 1.2 eq.). followed by DIPEA (0.43 g, 3.39 mmol, 1.5 eq.). Reaction mixture was stirred at room temperature for 3 h. Reaction progress was monitored by TLC, after completion, the mixture was diluted with H_2O (10 mL) and extracted using EtOAc (2 x 30 mL). The organic layer was washed with brine solution, dried over Na_2SO_4, filtered and concentrated to give crude compound. The resulting crude was purified by flash column

chromatography eluting with 15 % EtOAc/n-hexane. Two fractions were isolated, characterised by ^1H NMR and compared with reported value. Ethyl 2-chloro-4-
morpholinopyrimidine-5-carboxylate, **9C-2** (0.07 g, yield 11%) was isolated as a white solid. m/z: 272.11 $[M+H]^+$

^1H NMR (400 MHz, Chloroform-d) δ 8.57 (s, 1H), 4.36 (q, J = 7.1 Hz, 2H), 3.83 – 3.75 (m, 4H), 3.68 – 3.61 (m, 4H), 1.39 (t, J = 7.1 Hz, 3H).

Ethyl 4-chloro-2-morpholinopyrimidine-5-carboxylate, 9C-1 (0.12 g yield 19%) was isolated as a white solid. m/z: 272.08 $[M+H]^+$

^1H NMR (400 MHz, Chloroform-d) δ 8.84 (s, 1H), 4.37 (q, J = 7.0 Hz, 2H), 3.96 – 3.89 (m, 4H), 3.78 (d, J = 4.7 Hz, 4H), 1.39 (t, J = 7.1 Hz, 3H).

Synthesis of ethyl 4-(4-(cyclopropanecarboxamido)phenyl)-2morpholino-pyrimidine-5-carboxylate (**10**): To a stirred solution of ethyl 4-chloro-2-morpholinopyrimidine-5carboxylate, **9C-1** (0.25 g, 0.92 mmol, 1.0 eq.) in 1,4-dioxane (5.0 mL) was added N-(4-(4,4,5,5-tetramethyl-1,3,2-dioxaborolan-2-yl)phenyl)cyclopropanecarboxamide (**7B**) (0.29 g, 1.01 mmol, 1.1 eq.) and K_2CO_3 (0.19 g, 1.38 mmol, 1.5 eq.) in H_2O (5.0 mL) was then added. The reaction mixture was degassed with nitrogen for 20 min and $(PPh_3)_2PdCl_2$ (0.003 g, 0.046 mmol, 0.05 eq.) was added to it. The reaction mixture was stirred at 90 °C for 12 h. Reaction progress was monitored by TLC and LCMS, after completion, the mixture was diluted with H_2O (10 mL) and extracted using EtOAc (3 x 20 mL). The organic layer was washed with brine solution, dried over Na2SO4, filtered and concentrated to give crude compound. The resulting crude was purified by flash column

chromatography eluting the product with 30 % EtOAc/n-hexane. The pure fraction was collected and concentrated under reduced pressure to give ethyl 4-(4-(cyclopropanecarboxamido) phenyl)-2-morpholinopyrimidine-5-carboxylate (**10**) (0.03 g, 8%) as an off white solid. m/z: 397.23 $[M+H]^+$

^{1}H NMR (400 MHz, DMSO-d6) δ 10.40 (s, 1H), 8.74 (s, 1H), 7.66 (d, J = 8.3 Hz, 2H), 7.50 (d, J = 8.4 Hz, 2H), 4.11 (q, J = 6.7 Hz, 2H), 3.85 (s, 4H), 3.68 (s, 4H), 1.81 (d, J = 6.2 Hz, 1H), 1.08 (t, J = 6.9 Hz, 3H), 0.83 (s, 4H).

^{13}C NMR (176 MHz, DMSO) δ 172.36, 166.71, 166.34, 161.12, 141.33, 132.96, 129.99, 118.39, 112.41, 66.39, 60.84, 44.37, 15.11, 14.24, 7.85.

Synthesis of 4-(4-(cyclopropanecarboxamido)phenyl)-2morpholinopyrimidine-5-carboxylic acid (9A): To a stirred solution of ethyl 4-(4-(cyclopropanecarboxamido)phenyl)-2morpholino pyrimidine-5-carboxylate (**9B**) (0.10 g, 0.25 mmol, 1.0 eq.) in THF (2.0 mL) and EtOH (1.0 mL) was added NaOH (0.015 g, 0.38 mmol, 1.5 eq) in H_2O (1.0 mL). The reaction mixture was stirred at room temperature for 12 h. Reaction progress was monitored by TLC and LCMS, after completion, the mixture was diluted with H_2O (10 mL) and extracted using EtOAc (1 x 10 mL). The aqueous layer was acidified using dilute hydrochloride, solid formed was filtered and dried to give 4-(4-(cyclopropane-carboxamido)phenyl)-2morpholinopyrimidine-5-carboxylic acid (**9A**) (0.042 g. 45%) as a white solid. m/z: 369.19 $[M+H]^+$

^{1}H NMR (400 MHz, DMSO-d6) δ 10.49 (s, 1H), 8.75 (s, 1H), 7.66 (d, J = 8.5 Hz, 2H), 7.52 (d, J = 8.4 Hz, 2H), 3.84 (s, 4H), 3.67 (s, 4H), 1.89 – 1.81 (m, 1H), 0.82 (s, 4H).

13C NMR (176 MHz, DMSO) δ 172.35, 167.56, 166.85, 161.24, 161.09, 141.21, 133.21, 130.07, 118.35, 113.05, 66.41, 44.35, 15.09, 7.82.

Synthesis of tert-butyl (3-(4-(4-(cyclopropane carboxamide) phenyl)2-morpholinopyrimidine-5-carboxamido) propyl) carbamate (9): To a stirred solution of 4-(4-(cyclopropanecarboxamido)phenyl)-2morpholinopyrimidine-5-carboxylic acid (**9A**) (0.15 g, 0.40 mmol, 1.0 eq.) in DMF (3.0 mL) was added EDC.HCl (0.15 g, 0.61 mmol, 1.5 eq), HOBt (0.027 g, 0.2 mmol, 0.5 eq) and DIPEA (0.08 g, 0.60 mmol, 1.5 eq) at 0 °C. After stirring for 15 mins at the same temperature tert-butyl (3-aminopropyl)carbamate (0.08 g, 0.44 mmol, 1.1 eq) was added to above mixture and the reaction was allowed to warm to room temperature and stir for an additional 12 h. Reaction progress was monitored by TLC and LCMS, after completion, the mixture was diluted with H_2O (10 mL) and extracted using EtOAc (3 x 10 mL). The organic layer was washed with brine solution, dried over Na_2SO_4, filtered and concentrated to give crude compound. The resulting crude was purified by flash column chromatography eluting the product with 40 % EtOAc/n-hexane. The desired fractions were collected and concentrated to give tert-butyl (3-(4-(4-(cyclopropanecarboxamide) phenyl)2-morpholinopyrimidine-5-carboxamido) propyl) carbamate (**9**) (0.05 g) as a white solid. (yield-23%). m/z: 525.4 $[M+H]^+$

^1H NMR (400 MHz, DMSO-d6) δ 10.39 (s, 1H), 8.37 (s, 1H), 8.23 (s, 1H), 7.64 (s, 4H), 6.77 (s, 1H), 3.80 (s, 4H), 3.34 (s, 4H), 3.15 – 3.08 (m, 2H), 2.89 (s, 2H), 1.80 (s, 1H), 1.52 (s, 2H), 1.37 (s, 9H), 0.82 (s, 4H).

^{13}C NMR (176 MHz, DMSO) δ 172.35, 167.42, 141.44, 129.71, 119.18, 118.73, 77.95, 66.47, 44.40, 40.49, 40.35, 40.23, 40.11, 40.00, 39.88,

39.76, 39.64, 38.03, 37.28, 29.68, 28.72, 15.08, 7.85.

Synthesis of N-(4-((2-fluorophenyl)(hydroxy)methyl)phenyl)acetamide (**11**):

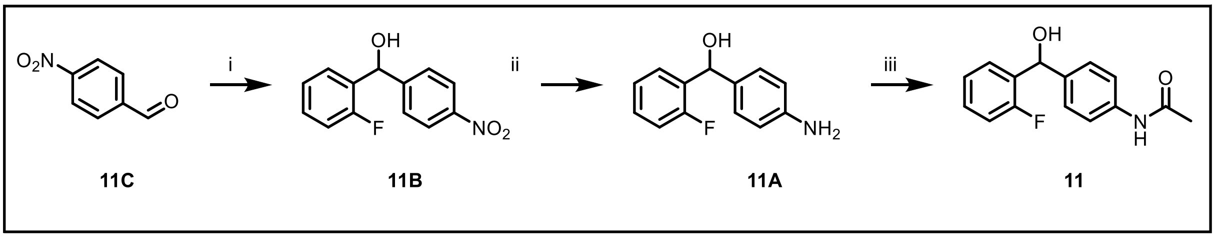

Supplementary Figure 16 – Synthesis of compound **11** : i. (2-fluorophenyl)boronic acid, tri-1-naphthyl phosphine, K_2CO_3, $Pd(OAc)_2$, [bmim][PF_6], water, 65 °C, 16 h, 42%; ii. Fe, NH_4Cl, EtOH, water, 70 °C, 4 hr, 56%; iii. Acetyl chloride, Na_2CO_3, dioxane, water, RT, 3 hr, 33.5%.

Synthesis of (4-aminophenyl)(2-fluorophenyl)methanol (11B): A mixture of 4-nitrobenzaldehyde (Int-1) (7.0 g, 19 mmol, 1 eq.) in THF (70 mL) and added (2-fluorophenyl)boronic acid (6.5 g, 26 mmol, 1.2 eq.) and K2CO3 (8.22 g, 59 mmol, 3 eq.). The reaction was degassed with nitrogen for 10 min, added Tri-1-naphthylphosphine (2.4 g, 10 mmol, 0.05 eq.) and palladium(II) chloride (1.16 g, 19 mmol, 0.05 eq.). It was stirred at 65 °C for 3 h. The reaction was monitored on TLC. After completion of the reaction, it was quenched with H2O (200 mL) and extracted with EtOAc (2 X 100 mL), dried over sodium sulfate and concentrated under reduced pressure to give the crude compound which was purified by flash column chromatography, eluting the desired product with 20% EtOAc: n-hexane to give (2-fluorophenyl)(4nitrophenyl) EtOH (**11B**) (7.0 g, 61.10%) as a cream solid. m/z: 246.0 $[M+H]^+$.

^1H NMR (400 MHz, DMSO-d_6) δ 8.20 (d, J = 8.6 Hz, 2H), 7.63 (d, J = 8.5 Hz, 2H), 7.55 (t, J = 7.0 Hz, 1H), 7.36 – 7.28 (m, 1H), 7.27 – 7.11 (m, 2H), 6.38 (d, J = 4.3 Hz, 1 OH), 6.08 (d, J = 4.0 Hz, 1H).

Synthesis of (4-aminophenyl)(2-fluorophenyl)methanol (11A): To a mixture of (2-fluorophenyl)(4-nitrophenyl) EtOH (Int-3) (0.14 g, 0.64 mmol, 1 eq.) in EtOH (1 mL) and H2O (1 mL), were added Fe powder (0.361 g, 6.4 mmol, 10.0 eq) and ammonium chloride (0.341 g, 6.4 mmol, 10.0 eq) and heated to reflux for 4 h. After completion of the reaction, the reaction was filtered

through celite-pad and concentrated under reduced pressure to give the desired product as (4-aminophenyl)(2-fluorophenyl)methanol (**11A**) (0.07 g, 56.9 %) as a solid. m/z: 218.0 $[M+H]^+$

^{1}H NMR (400 MHz, DMSO-d_6) δ 7.58 (t, J = 7.4 Hz, 1H), 7.21 (dt, J = 24.8, 7.2 Hz, 2H), 7.12 – 7.02 (m, 1H), 6.95 (d, J = 8.0 Hz, 2H), 6.47 (d, J = 8.1 Hz, 2H), 5.76 (d, J = 3.5 Hz, 1H), 5.65 (d, J = 4.1 Hz, 1H), 4.97 (s, 2H).

Synthesis of N-(4-((2-fluorophenyl)(hydroxy)methyl)phenyl)acetamide (11): To a mixture of (4-aminophenyl)(2-fluorophenyl) EtOH (Int-4) (0.1 g, 0.53 mmol, 1 eq.) in 1,4-dioxane (2 mL) and H2O (2 mL), was added sodium bicarbonate (0.085 g, 0.8 mmol, 1.5 eq.), cooled to 0 ^{o}C and acetyl chloride (0.05 g, 0.64 mmol, 1.2 eq.) was added to stir for 3 h at room temperature. After completion of the reaction, the reaction mixture quenched with H2O (20 mL) and extracted with EtOAc (2 X 20 mL), dried over sodium sulfate and concentrated under reduced pressure to give the crude compound which was purified with RP-HPLC purification using (i) 0.1% formic acid in H2O (ii) MeCN to give to N-(4-((2-fluorophenyl)(hydroxy)methyl)phenyl)acetamide (**11**) (0.04 g, 33.51 %) as a white solid. m/z: 260.0 $[M+H]^+$.

^{1}H NMR (400 MHz, DMSO-d_6) δ 9.90 (s, 1H), 7.57 (t, J = 1.6 Hz, 1H), 7.50 (t, J = 8.8 Hz, 2H), 7.25-7.20 (m, 5H), 5.94-5.89 (m, 2H), 2.02 (s, 3H).

^{13}C NMR (176 MHz, DMSO) δ 168.62, 160.14, 139.37, 138.64, 133.05, 129.16, 128.16, 127.05, 124.80, 119.26, 115.52, 68.08, 24.41.

Synthesis of N-((4-acetamidophenyl)(2-fluorophenyl)methyl)-2-chlorobenzamide (12):

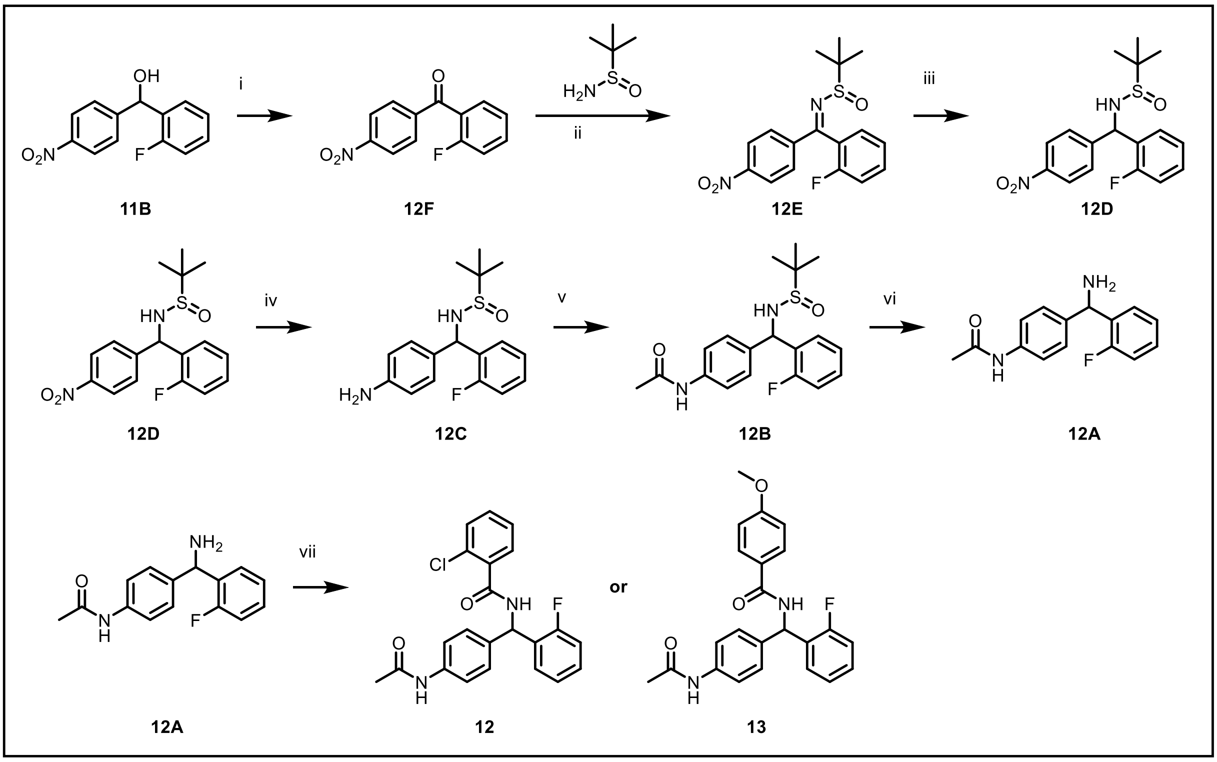

Supplementary Figure 17 - Synthesis of compound **12**: i. PCC, DCM, RT, 12 hr, 50%; ii. Titanium ethoxide, dioxane, 100 °C, 3 hr, 75%; iii. $NaBH_4$, MeOH, 0 °C, 30 min, 78%; iv. Pd/C, H_2, MeOH, RT, 5 hr, 81%; v. acetic anhydride, AcOH, RT, 2 hr, 55%; vi. HCl (4 M in dioxane), RT, 2 hr, 19%; vii. 2-chlorobenzoic acid or 4-methoxybenzoic acid, HATU, DIPEA, THF, RT, 2 hr, 7-35%.

Synthesis of (4-aminophenyl)(2-fluorophenyl)methanol (11B): A mixture of 4-nitrobenzaldehyde (**11C**) (7.0 g, 19 mmol, 1 eq.) in THF (70 mL) and added (2-fluorophenyl)boronic acid (6.5 g, 26 mmol, 1.2 eq.) and K2CO3 (8.22 g, 59 mmol, 3 eq.). The reaction was degassed with nitrogen for 10 min, added Tri-1-naphthylphosphine (2.4 g, 10 mmol, 0.05 eq.) and palladium(II) chloride (1.16 g, 19 mmol, 0.05 eq.). It was stirred at 65 °C for 3 h. The reaction was monitored on TLC. After completion of the reaction, it was quenched with H_2O (200 mL) and extracted with EtOAc (2 X 100 mL), dried over sodium sulfate and concentrated under reduced pressure to give the crude compound which was purified by flash column chromatography, eluting the desired product with 20% EtOAc: n-hexane to give (4-

aminophenyl)(2-fluorophenyl)methanol (**11B**) (7.0 g, 61.10%) as a cream solid. m/z: 246.0 [M-H]$^-$,

^{1}H NMR (400 MHz, DMSO-d_6) δ 8.20 (d, J = 8.6 Hz, 2H), 7.63 (d, J = 8.5 Hz, 2H), 7.55 (t, J = 7.0 Hz, 1H), 7.36 – 7.28 (m, 1H), 7.27 – 7.11 (m, 2H), 6.38 (d, J = 4.3 Hz, 1 OH), 6.08 (d, J = 4.0 Hz, 1H).

Synthesis of (2-fluorophenyl)(4-nitrophenyl)methanone (12F): To a mixture of (4-aminophenyl)(2-fluorophenyl)methanol (**11B**) (7.0 g, 27 mmol, 1 eq.) in dichloromethane (20 mL) at 0 °C, was added pyridinium chlorochromate (8.7 g, 40 mmol, 1.5 eq.) and stirred for 1h. Reaction was monitored on TLC. After completion of the reaction, the reaction mixture was quenched with H2O (200 mL) and extracted with EtOAc (2 X 100 mL), dried over sodium sulfate and concentrated under reduced pressure to give the crude compound **12F** which was forwarded for the next step without purification. m/z: 246.22 [M+1].

Synthesis of (Z)-N-((2-fluorophenyl)(4-nitrophenyl)methylene)-2-methyl propane-2-sulfinamide (12E): To a mixture of (2-fluorophenyl)(4-nitrophenyl)methanone (**12F**) (6.0 g, 24 mmol, 1 eq.) in 1,4-dioxane (60 mL), was added titanium(IV) ethoxide (16.75 g, 73 mmol, 3 eq.) and 2-methylpropane-2-sulfinamide (3.58 g, 29 mmol, 1.2 eq.) and stirred 100° C for 4 h. The reaction was monitored on TLC. After completion of the reaction, the reaction mixture was quenched with H2O (400 mL) and extracted with EtOAc (2 X 100 mL), dried over sodium sulfate and concentrated under reduced pressure to give the crude compound which was purified by flash column chromatography, eluting the desired product with 30% EtOAc: n-hexane to give (Z)-N-((2fluorophenyl)(4-nitrophenyl)methylene)-2-methyl propane-2-sulfinamide (**12E**) (5.9 g, 69.22%) as a solid. m/z: 349.1 [M+H]$^+$.

^{1}H NMR (400 MHz, DMSO-d_6) δ 8.35 (d, J = 8.6 Hz, 2H), 7.85 (d, J = 8.3 Hz, 2H), 7.63 (d, J = 6.2 Hz, 1H), 7.41 (dd, J = 18.8, 10.0 Hz, 3H), 1.27 (s, 9H).

Synthesis of N-((2-fluorophenyl)(4-nitrophenyl)methyl)-2methylpropane-2-sulfinamide (12D): To a mixture of (Z)-N-((2-fluorophenyl)(4-nitrophenyl)methylene)-2methylpropane-2-sulfinamide (**12E**) (5.9 g, 16 mmol, 1 eq.) in EtOH (60 mL), cooled at 0°C added sodium borohydride (0.576 g, 16 mmol, 1 eq.) then stirred reaction mass at 1h. Reaction was monitoring on TLC. After completion of the reaction, the reaction mixture quenched with H2O (200 mL) and extracted with EtOAc (2 X 100 mL), dried over sodium sulfate and concentrated under reduced pressure to give the crude compound which was purified by flash column chromatography, eluting the desired product with 40% EtOAc: n-hexane to give N-((2-fluorophenyl)(4-nitrophenyl)methyl)2-methylpropane-2-sulfinamide (**12D**) (5.8 g, 97.72%) as a cream solid. m/z: 351 $[M+H]^+$

Synthesis of N-((4-aminophenyl)(2-fluorophenyl)methyl)-2methylpropane-2-sulfinamide (12C): To a mixture of N-((2-fluorophenyl)(4-nitrophenyl)methyl)-2-methylpropane-2-sulfinamide (**12D**) (5.8 g, 17 mmol, 1 eq.) in EtOH (120 mL), degassed with nitrogen, added Pd/C (0.7% with 50% moisture) (0.7 g) and stirred under hydrogen balloon pressure for 1 h. The reaction mixture was filtered through celite-pad, washed with EtOH, the filtrate was concentrated under reduced pressure to give N-((4-aminophenyl)(2fluorophenyl)methyl)-2-methylpropane-2-sulfinamide (**12C**) (5.3 g, 98.22%) as a solid. m/z: 320.49 $[M+H]^+$
^{1}H NMR (400 MHz, DMSO-d_6) δ 7.76 (t, J = 7.7 Hz, 1H), 7.45 (d, J = 8.3 Hz, 2H), 7.22 (dd, J = 20.4, 7.8 Hz, 2H), 7.06 – 6.96 (m, 1H), 6.50-6.46 (m, 2H), 5.73 (s, 1H), 5.08 (m, 2H, NH_2), 1.14 – 1.05 (m, 9H). (Mixture of atropisomers)

Synthesis of N-(4-(((tert-butylsulfinyl)amino)(2-fluorophenyl)methyl)phenyl)acetamide (12B): To a mixture of N-((4-aminophenyl)(2-fluorophenyl)methyl)-2methylpropane-2-sulfinamide (**12C**) (5.3 g, 16 mmol 1 eq.) in AcOH (10 mL) at 0 °C, was added acetic anhydride (2.56 g, 25 mmol, 1.5 eq.) and stirred for 1h. The reaction was monitored on LC-MS. After completion of the reaction, the reaction mixture was quenched with H2O (200 mL) and extracted with EtOAc (2 X 100 mL), dried over sodium sulfate and concentrated under reduced pressure to give the crude compound which was purified by flash column chromatography, eluting the desired product with 40% EtOAc: n-hexane to give N-(4-(((tert-butylsulfinyl)amino)(2-fluorophenyl)methyl)phenyl)acetamide (**12B**) (5.9 g, 98.39%) as a solid. m/z: 363.0 $[M+H]^+$

^{1}H NMR (400 MHz, DMSO-d_6) δ 9.94 (d, J = 3.6 Hz, 1H), 7.66 – 7.58 (m, 3H), 7.54 – 7.42 (m, 2H), 7.22 (ddt, J = 32.5, 26.0, 9.1 Hz, 4H), 6.04 (dd, J = 21.4, 6.4 Hz, 1H), 5.79 – 5.66 (m, 1H), 2.00 (d, J = 8.0 Hz, 3H), 1.13 (s, 9H). (Mixture of atropisomers).

Synthesis of N-(4-(amino(2-fluorophenyl)methyl)phenyl)acetamide (12A): To a solution of N-(4-(((tert-butylsulfinyl)amino)(2-fluorophenyl)methyl)phenyl) acetamide (**12B**) (5.9 g, 16 mmol, 1 eq.) in 1,4-dioxane (30 mL), was added HCl in 1,4-dioxane (4.0 M, 30 mL) and stirred for 1h. The reaction mixture was concentrated to give crude which was purified with RP-HPLC using 0.1% formic acid in H_2O/ MeCN. Product fractions were collected and concentrated to give N-(4-(amino(2fluorophenyl)methyl)phenyl) acetamide (**12A**) (3.01 g, 73.39%) as a cream solid. m/z: 242.1 [M-16]

^{1}H NMR (400 MHz, DMSO-d_6) δ 10.00 (s, 1H), 7.66 (t, J = 7.2 Hz, 1H), 7.53 (d, J = 8.4 Hz, 2H), 7.31 (p, J = 9.9, 8.5 Hz, 4H), 7.27 – 7.12 (m, 1H), 5.49 (s, 1H), 2.02 (s, 3H).

^{13}C NMR (176 MHz, DMSO) δ 168.73, 163.70, 160.41, 159.02, 139.03, 129.68, 128.52, 127.80, 125.05, 119.43, 115.89, 51.86, 24.40.

Synthesis of N-((4-acetamidophenyl)(2-fluorophenyl)methyl)-2chloro benzamide (12): To a mixture of N-(4-(amino(2-fluorophenyl)methyl)phenyl)acetamide (Int-9) (0.13 g, 0.5 mmol, 1 eq.) in THF (2 mL), was added 2-chlorobenzoic acid (0.094 g, 0.6 mmol, 1.2 eq.), stirred for 10 min, added HATU (0.3 g, 0.75 mmol, 1.5 eq.) and DIPEA (0.2 g, 1.5 mmol, 3 eq.) and stirred for 3 h.

After completion of the reaction, the reaction mixture was quenched with H_2O (20 mL) and extracted with EtOAc (2 X 10 mL), dried over Na_2SO_4 and concentrated under reduced pressure to give the crude compound which was purified by flash column chromatography, eluting the desired product with 80% EtOAc/n-hexane and purified further with HPLC purification using 0.1% TFA in H_2O/MeCN. Product fractions were collected and dried to give to N-((4-acetamidophenyl)(2-fluorophenyl)methyl)-2-chloro benzamide (12) as a solid (0.06 g, 30.0%). m/z: 397.3 $[M+H]^+$

^1H NMR (400 MHz, DMSO-d_6) δ 9.96 (s, 1H), 9.45 (d, J = 8.3 Hz, 1H), 7.55-7.30 (m, 8H), 7.25-7.19 (m, 4H), 6.52 (d, J = 8.4 Hz, 1H), 2.02 (s, 2H).

^{13}C NMR (176 MHz, DMSO) δ 168.70, 166.10, 160.72, 159.33, 138.90, 137.17, 135.57, 131.23, 130.39, 129.95, 129.68, 129.56, 129.48, 129.38, 129.10, 128.08, 127.51, 124.96, 124.94, 119.48, 115.81, 115.69, 50.08, 50.06, 24.40.

Synthesis of N-((4-acetamidophenyl)(2-fluorophenyl)methyl)-4methoxy benzamide (13): To a mixture of N-(4-(amino(2-fluorophenyl)methyl)phenyl)acetamide (**12A**) (0.15 g, 0.4 mmol, 1 eq.) in THF (2 mL), was added 4methoxybenzoic acid (0.087 g, 0.4 mmol, 1.2 eq.), stirred for 10 min, added HATU (0.178 g, 0.75 mmol, 1.5 eq.) and DIPEA (0.26 g, 2.2 mmol, 3 eq.) and stirred for 3 h. After completion of the reaction, the reaction mixture was quenched with H_2O (20 mL) and extracted with EtOAc (2 X 10 mL), dried over Na_2SO_4 and concentrated under reduced pressure to give the crude compound which was purified by flash column chromatography, eluting the desired product with 80% EtOAc: n-hexane and further with RP-HPLC purification using 0.1% formic acid in H_2O/MeCN to give to N-((4acetamidophenyl)(2-fluorophenyl)methyl)-4-methoxybenzamide (**13**) (0.016 g, 7.04%) as a solid. m/z: 393.43[M+H]$^+$

^{1}H NMR (400 MHz, DMSO-d_6) δ 9.96 (s, 1H), 9.08 (d, J = 8.3 Hz, 1H), 7.92 (d, J = 8.7 Hz, 2H), 7.50 (dd, J = 21.2, 7.8 Hz, 3H), 7.35 (d, J = 6.9 Hz, 1H), 7.20 (q, J = 8.1 Hz, 4H), 7.00 (d, J = 8.7 Hz, 2H), 6.59 (d, J = 8.4 Hz, 1H), 3.81 (s, 3H), 2.02 (s, 2H).

^{13}C NMR (176 MHz, DMSO) δ 168.69, 165.70, 162.19, 138.85, 135.96, 130.00, 129.63, 129.36, 128.27, 126.76, 124.92, 119.48, 115.63, 113.89, 55.85, 24.41.

1H and 13C NMR of Compound **2**

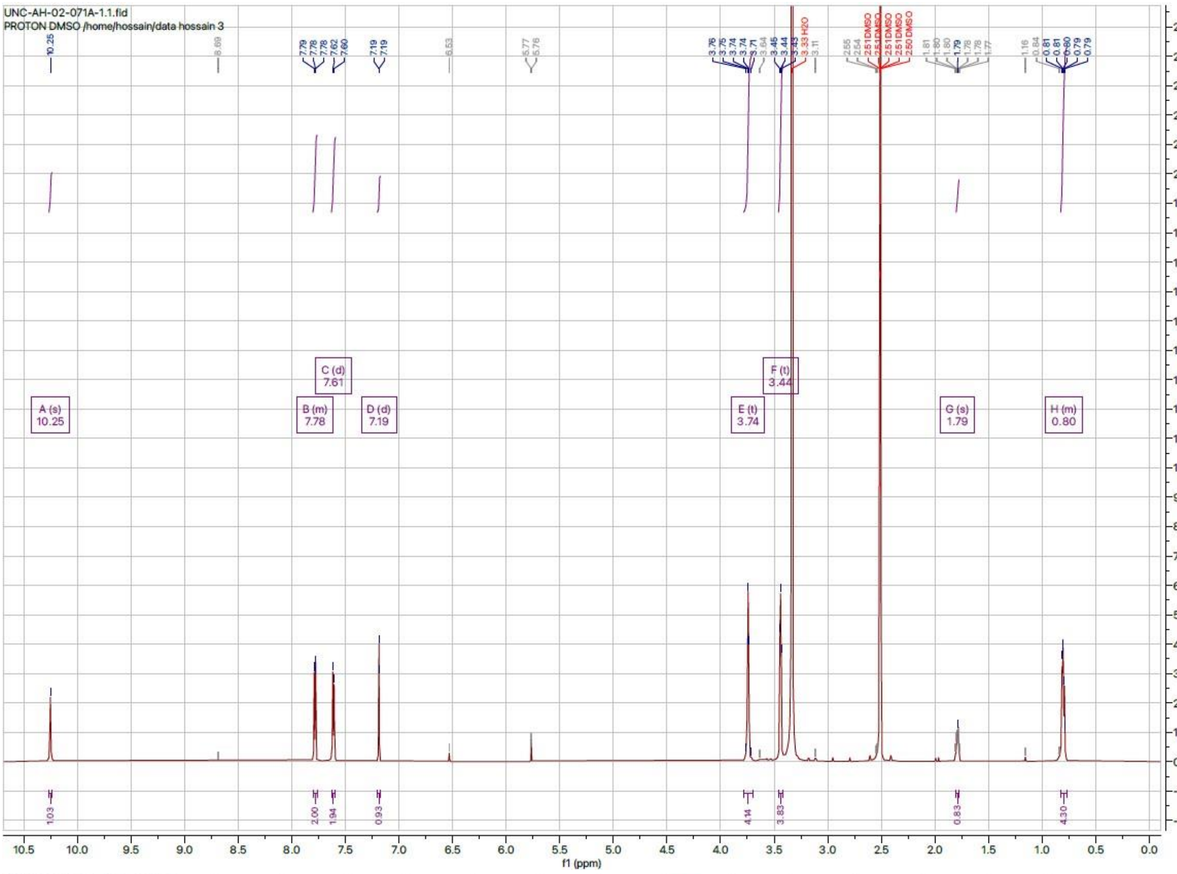

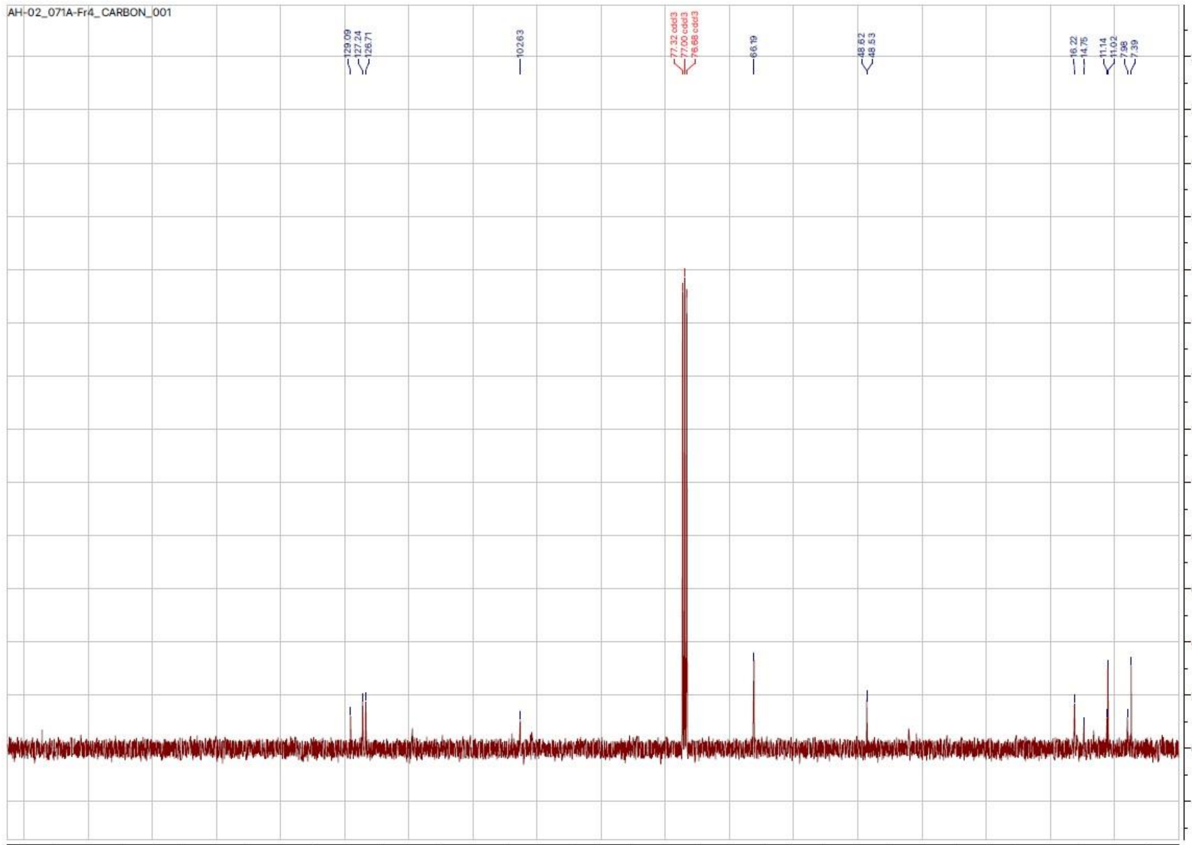

1H and 13C NMR of Compound **3**

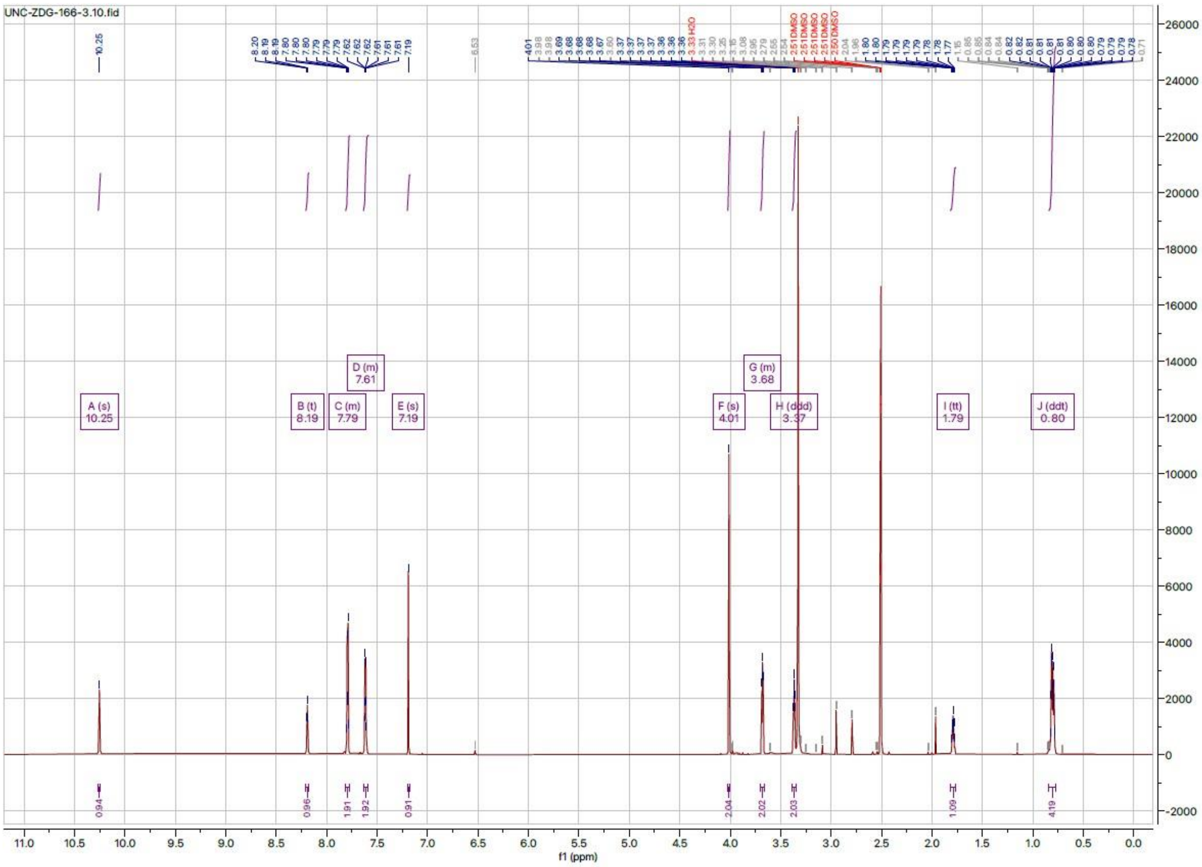

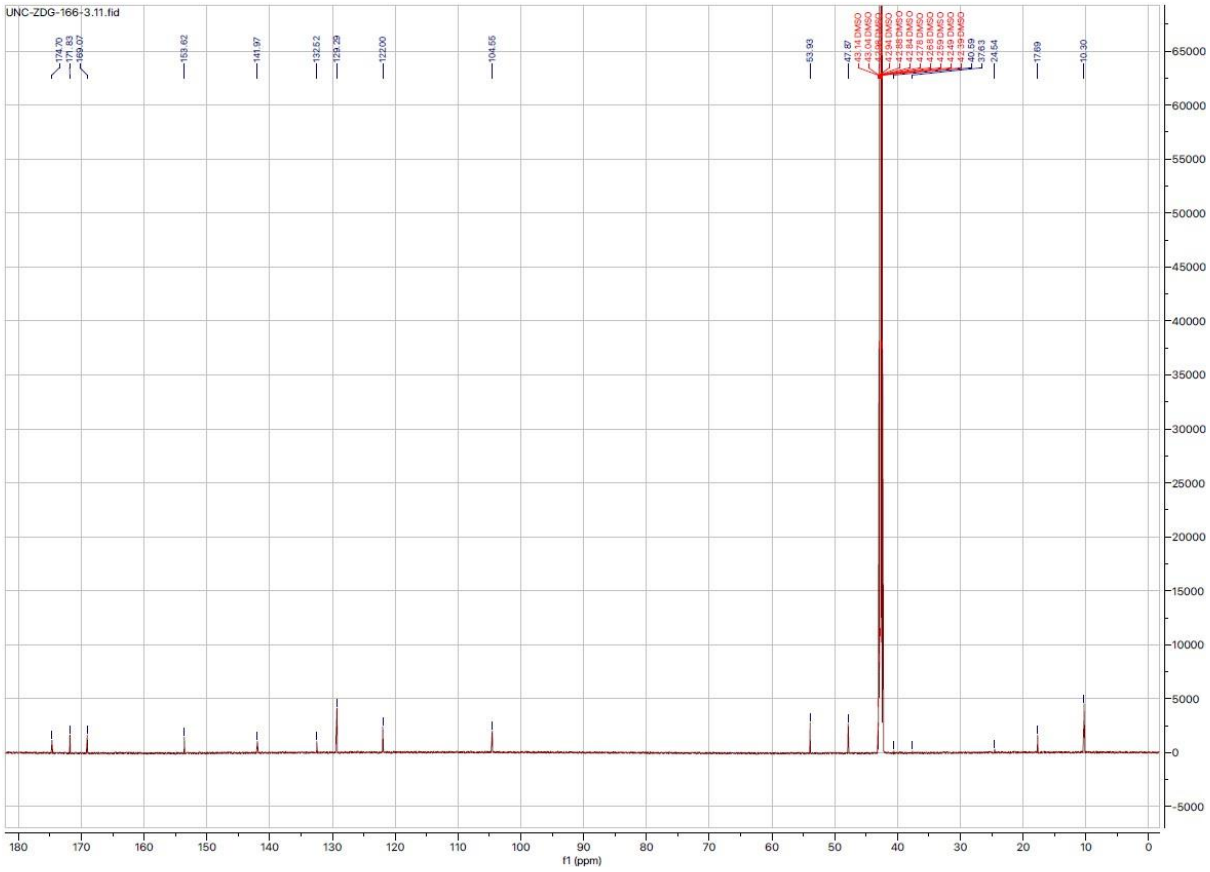

1H and 13C NMR of Compound **4**

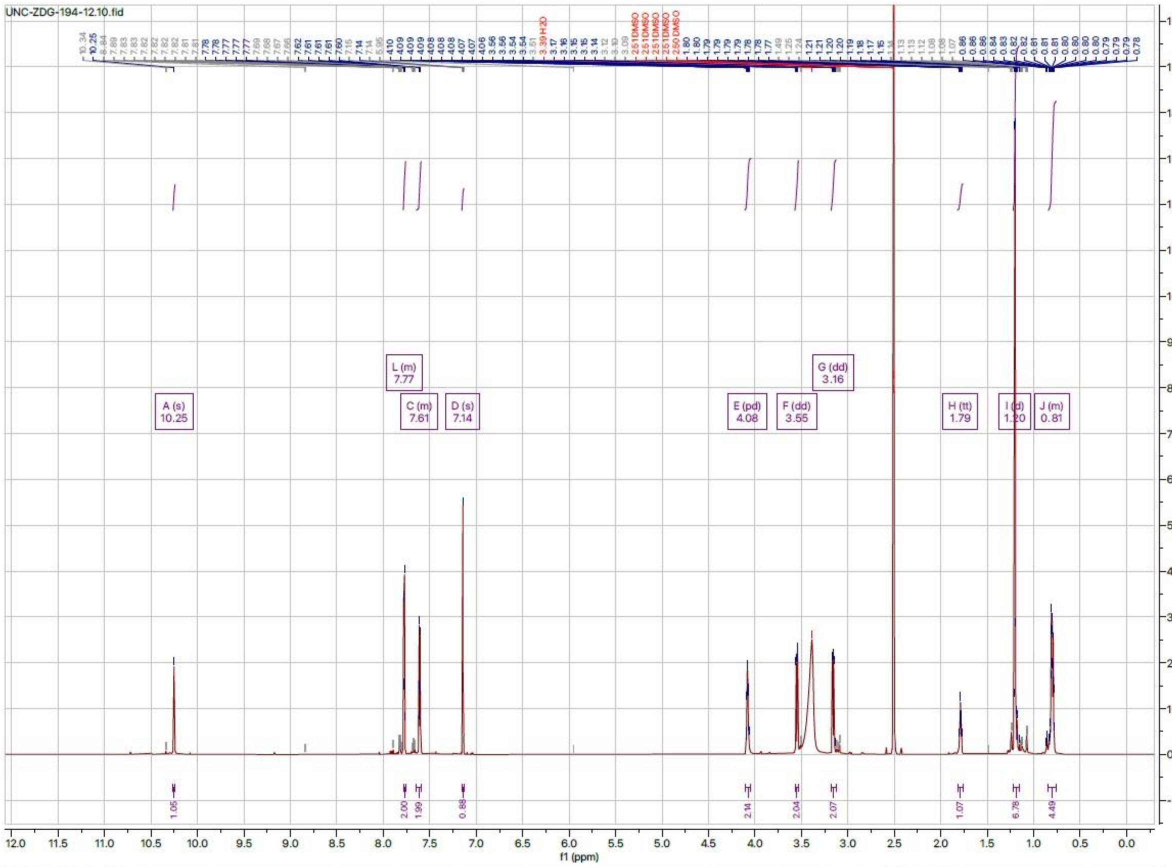

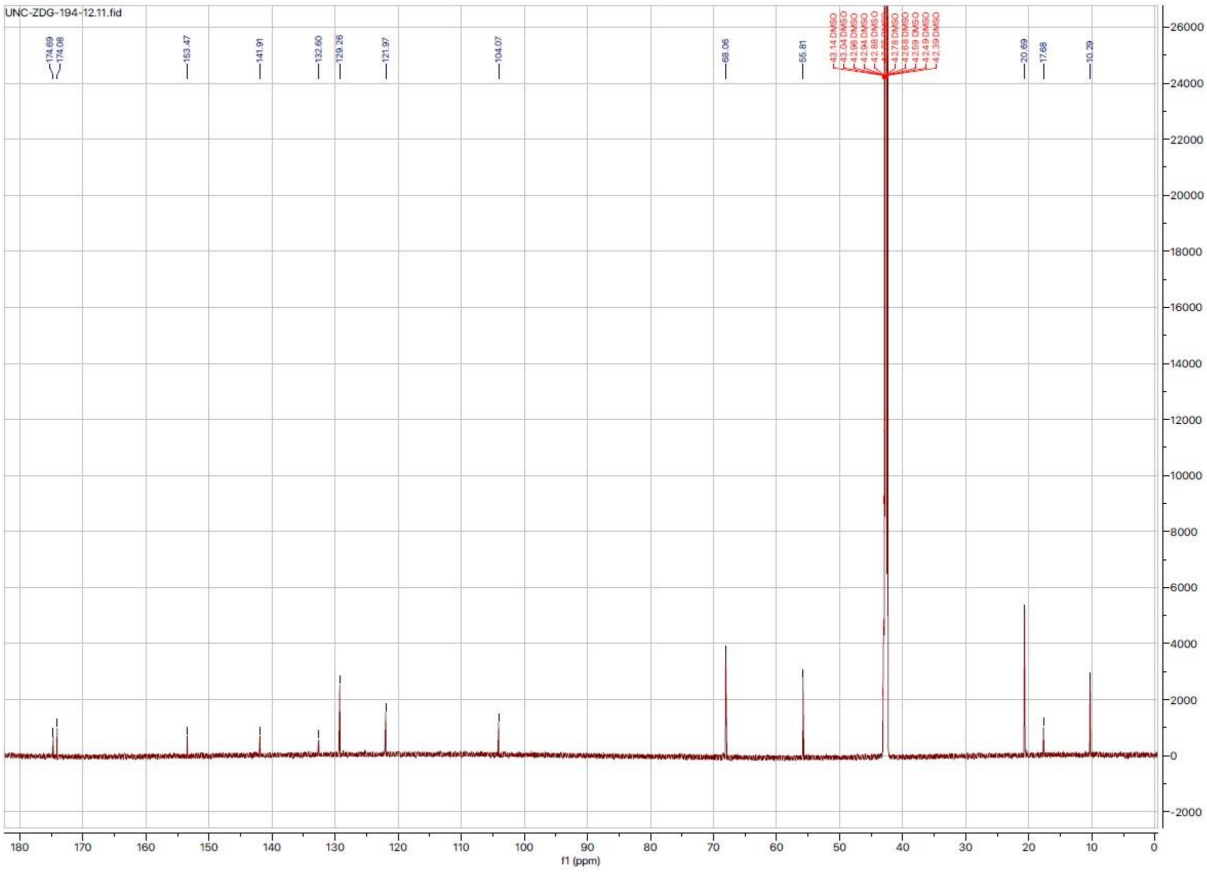

1H and 13C NMR of Compound 5

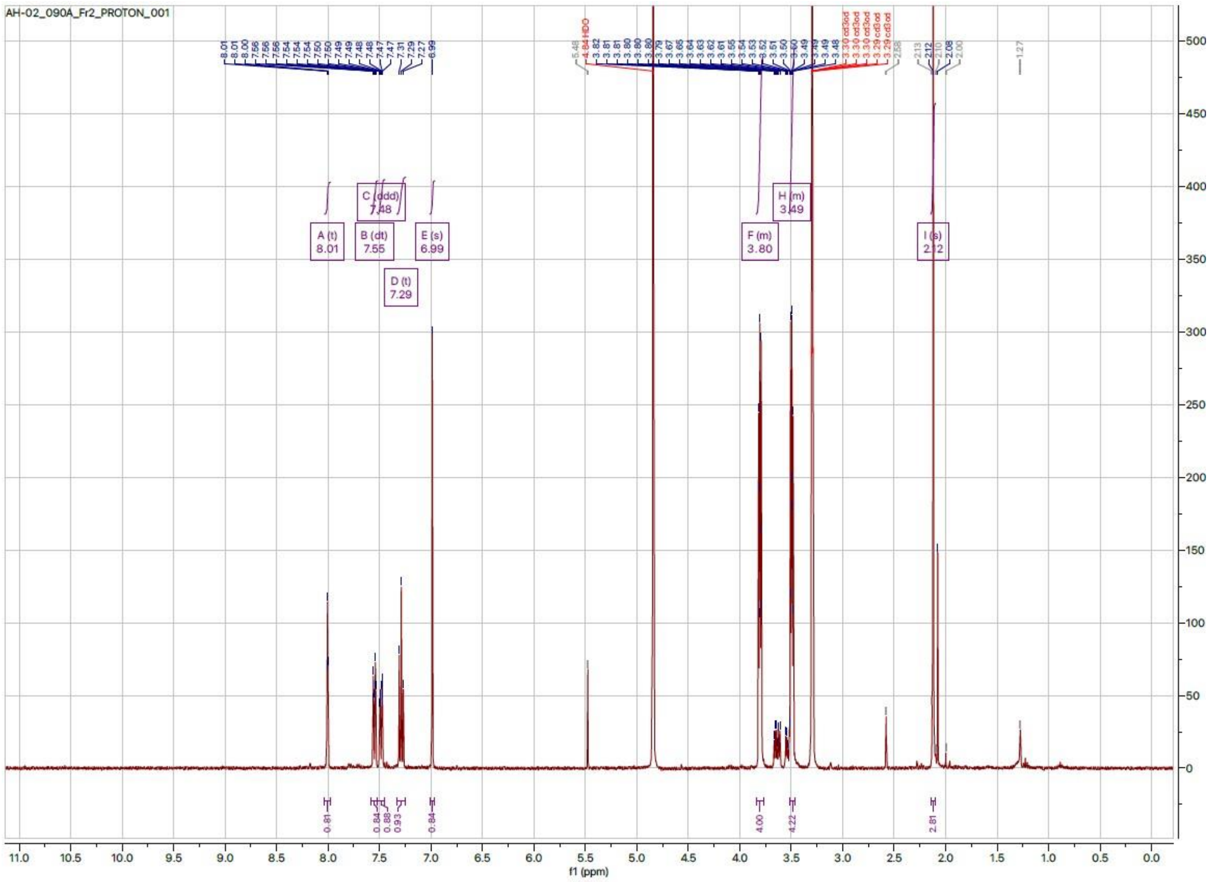

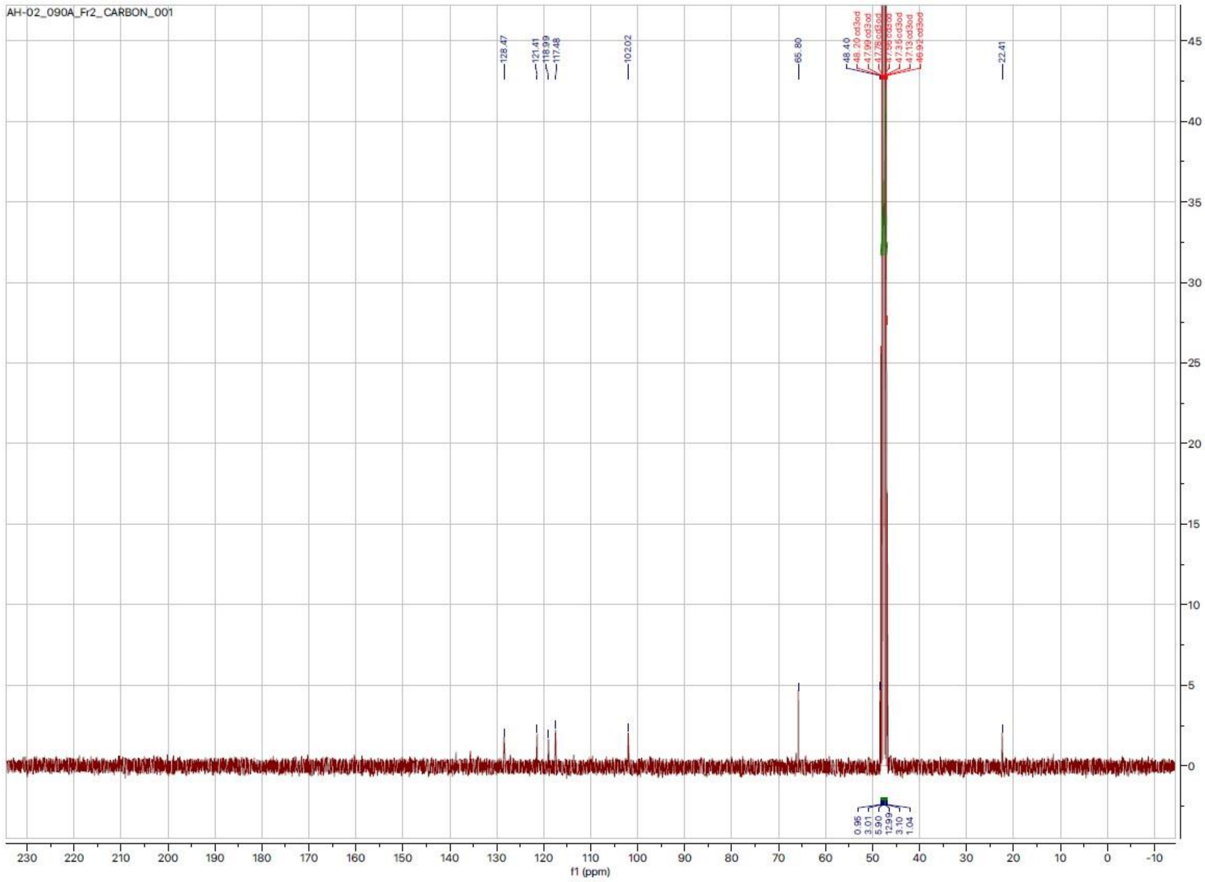

1H and 13C NMR of Compound **6**

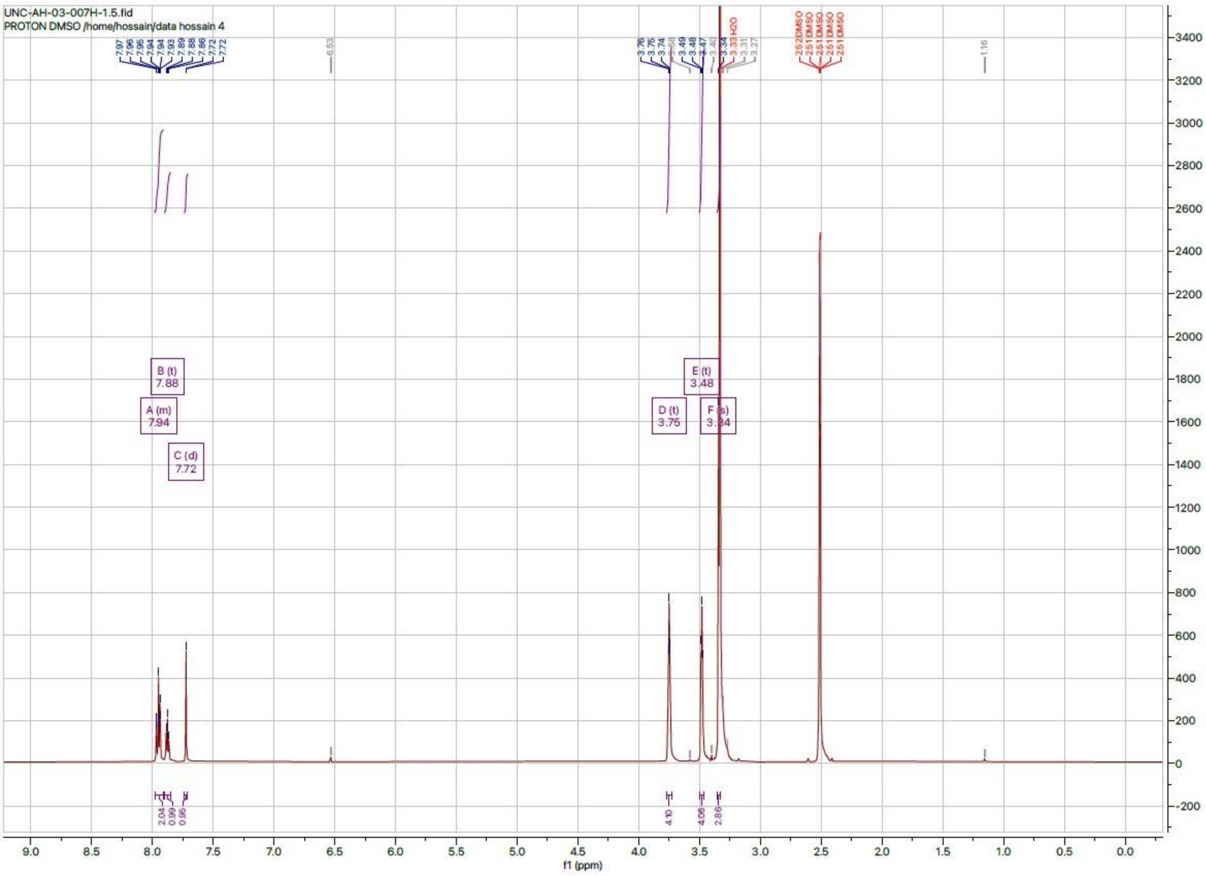

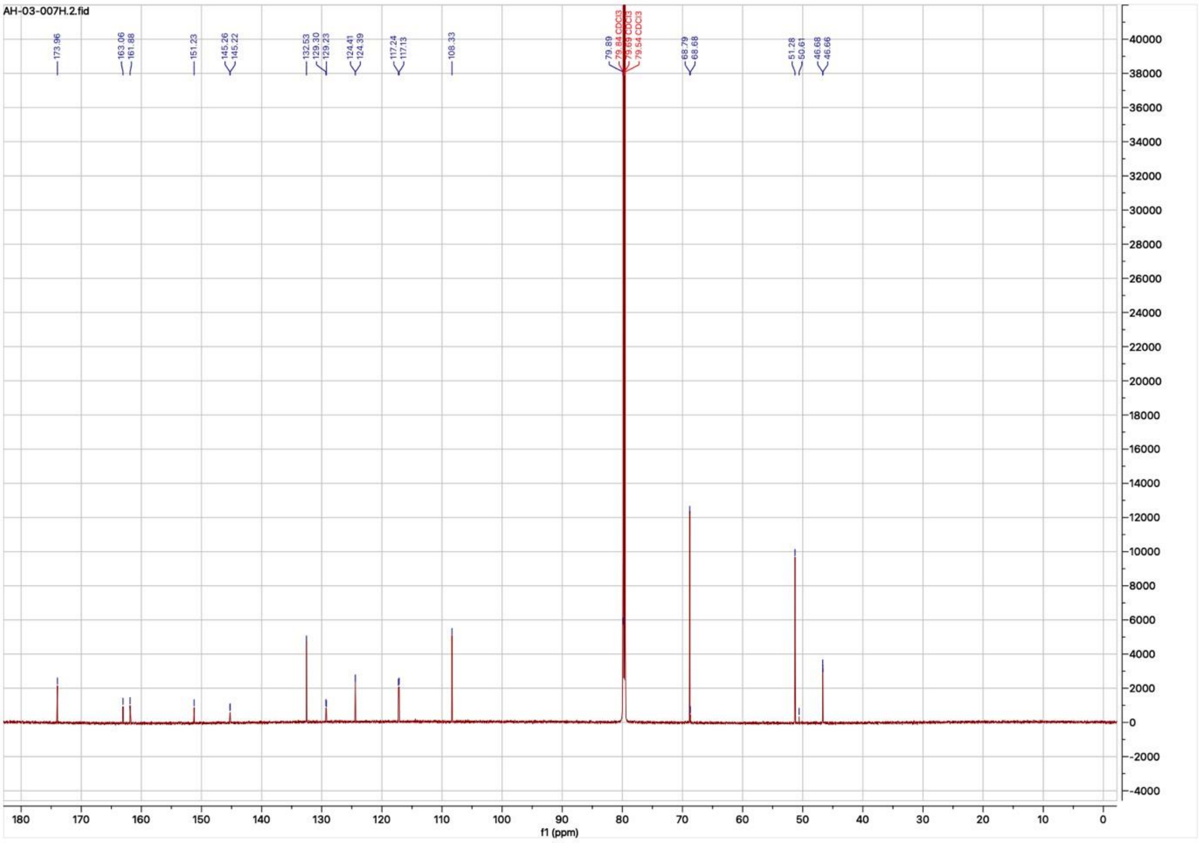

1H and 13C NR of Compound **7**

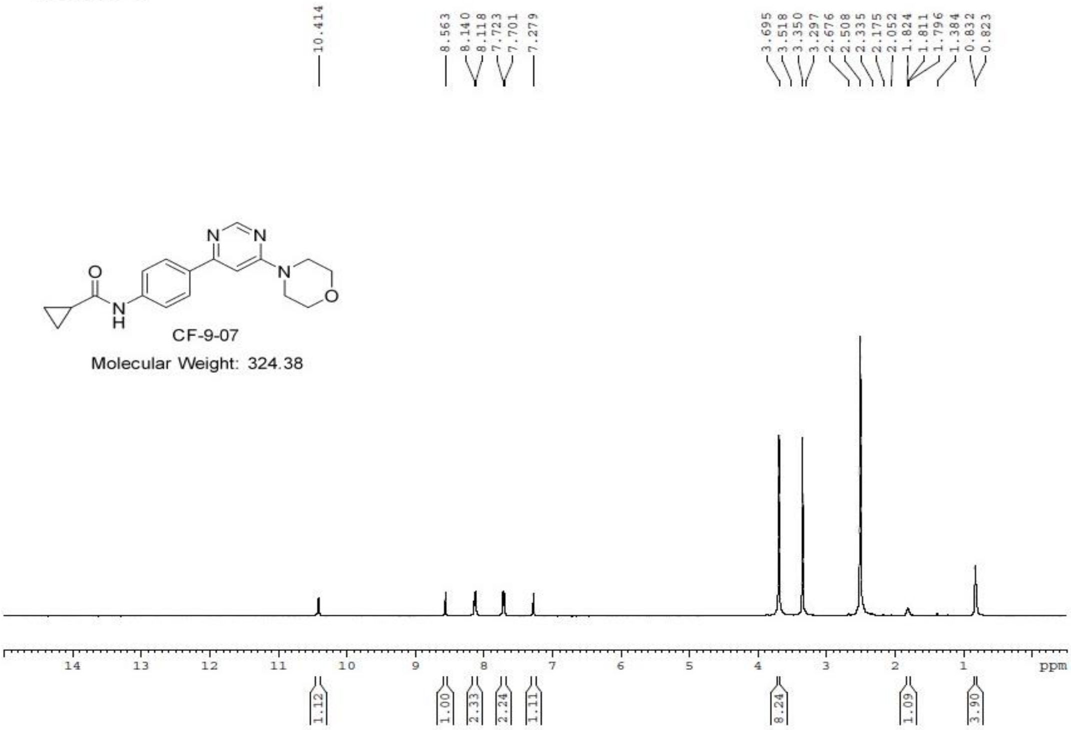

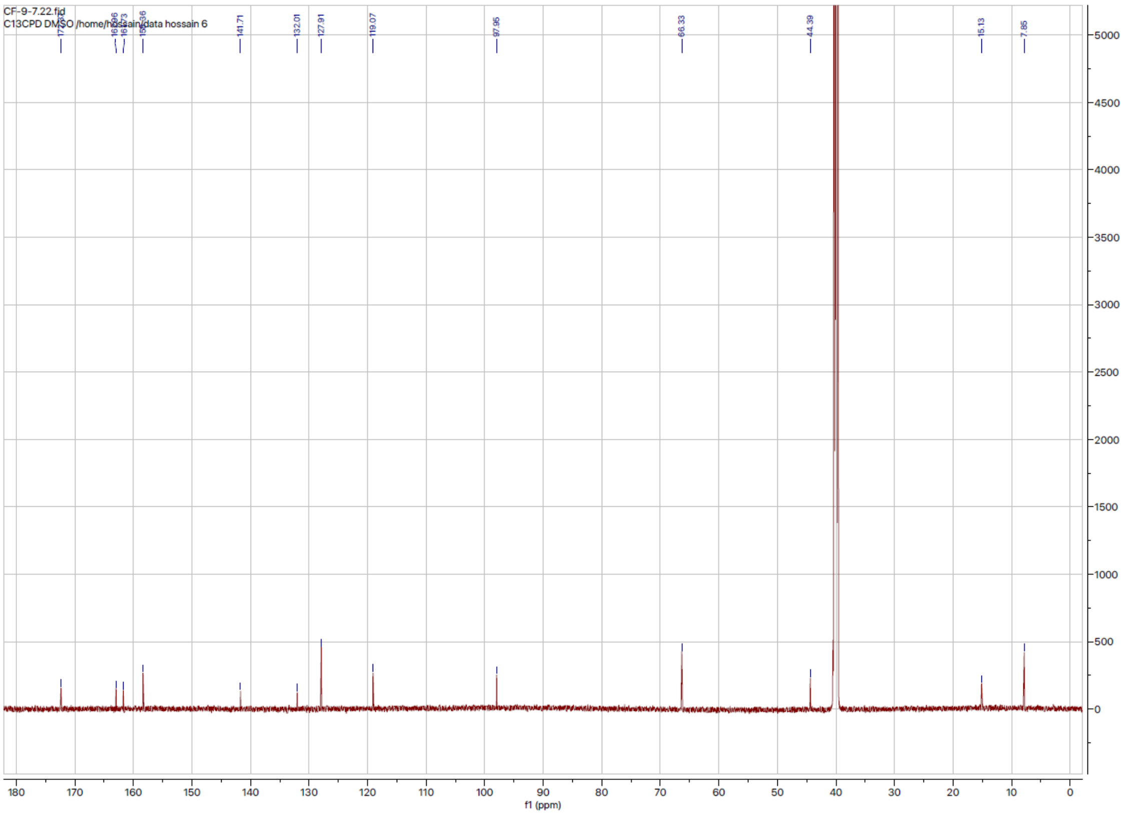

1H and 13C NMR of Compound **8**

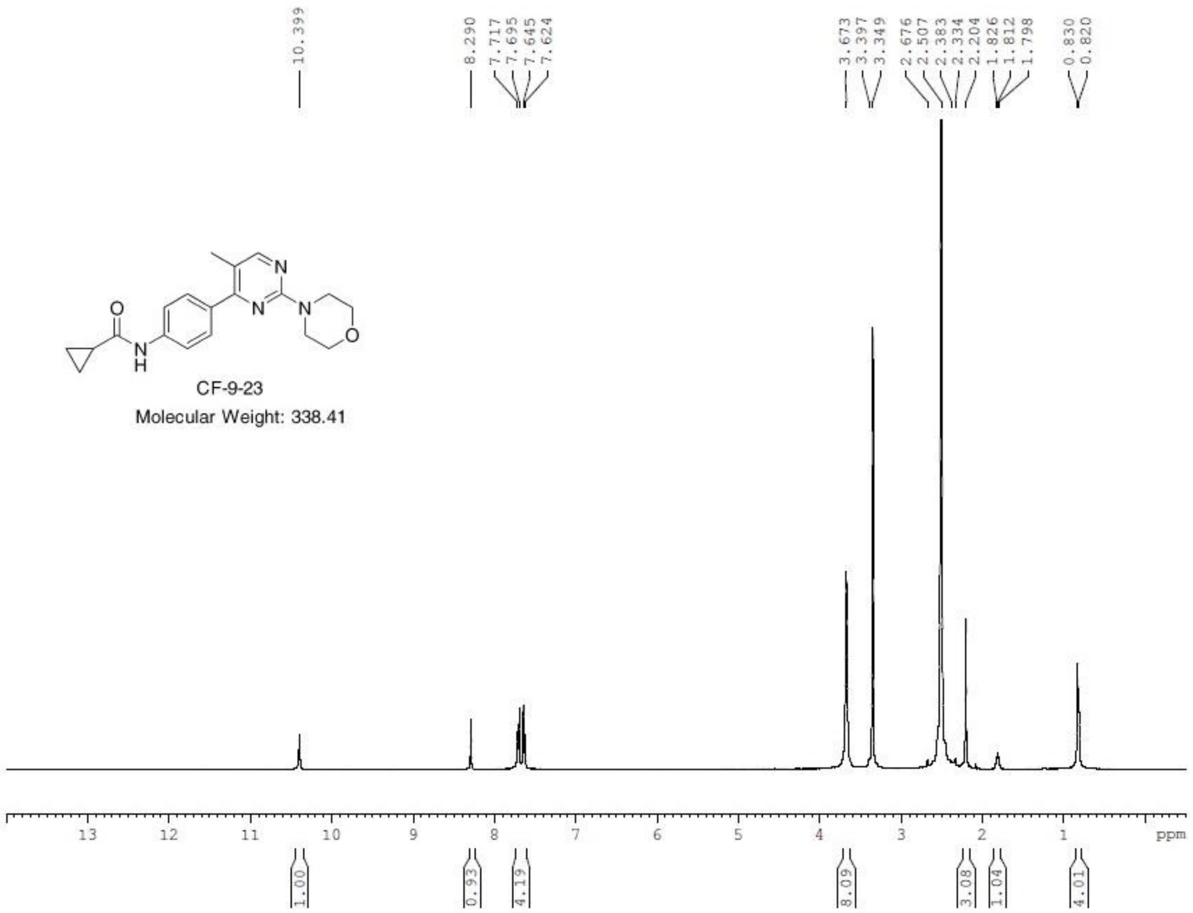
10.399
8.290
7.717
7.695
7.645
7.624
3.673
3.397
3.349
2.676
2.507
2.383
2.334
2.204
1.826
1.812
1.798
0.830
0.820
CF-9-23
Molecular Weight: 338.41
13 12 11 10 9 8 7 6 5 4 3 2 1 ppm
1.00
0.93
4.19
8.09
3.08
1.04
4.01

1H and 13C NMR of Compound 9

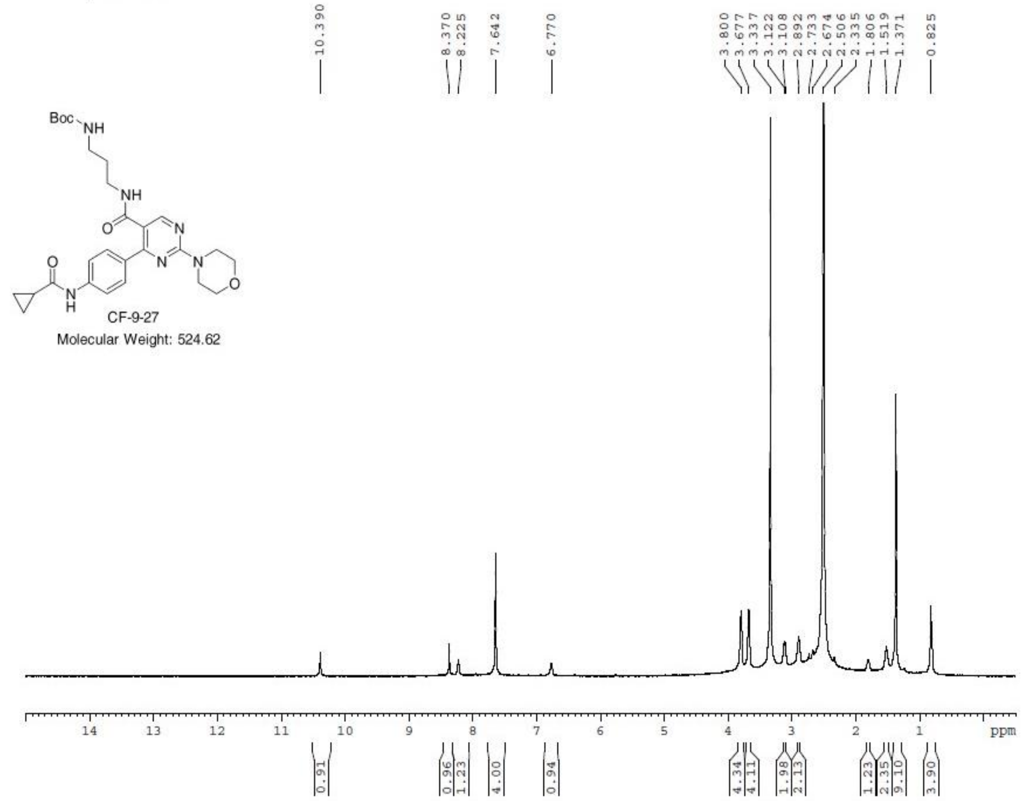

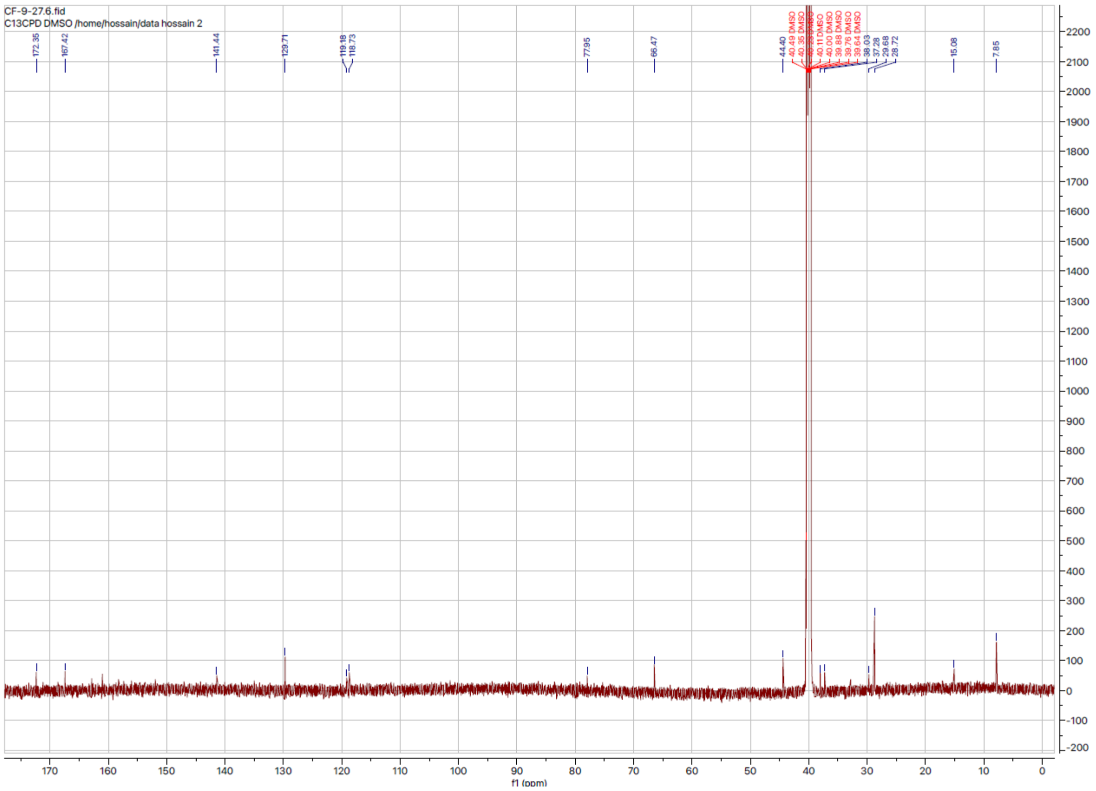

1H and 13C NMR of Compound 10

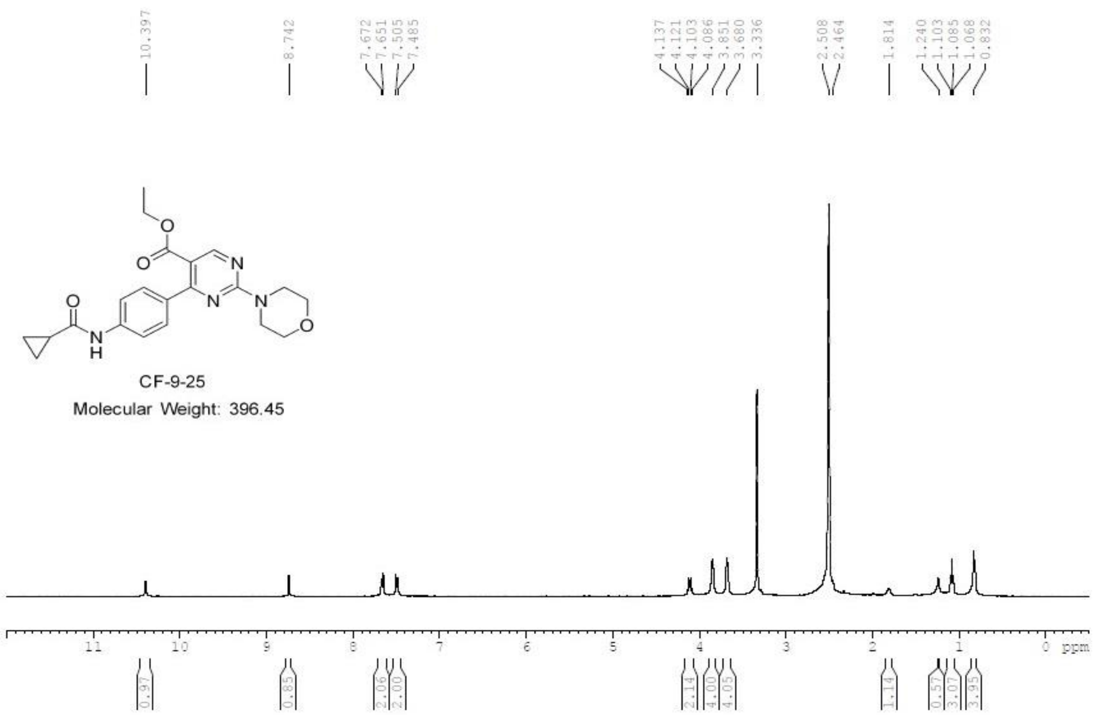

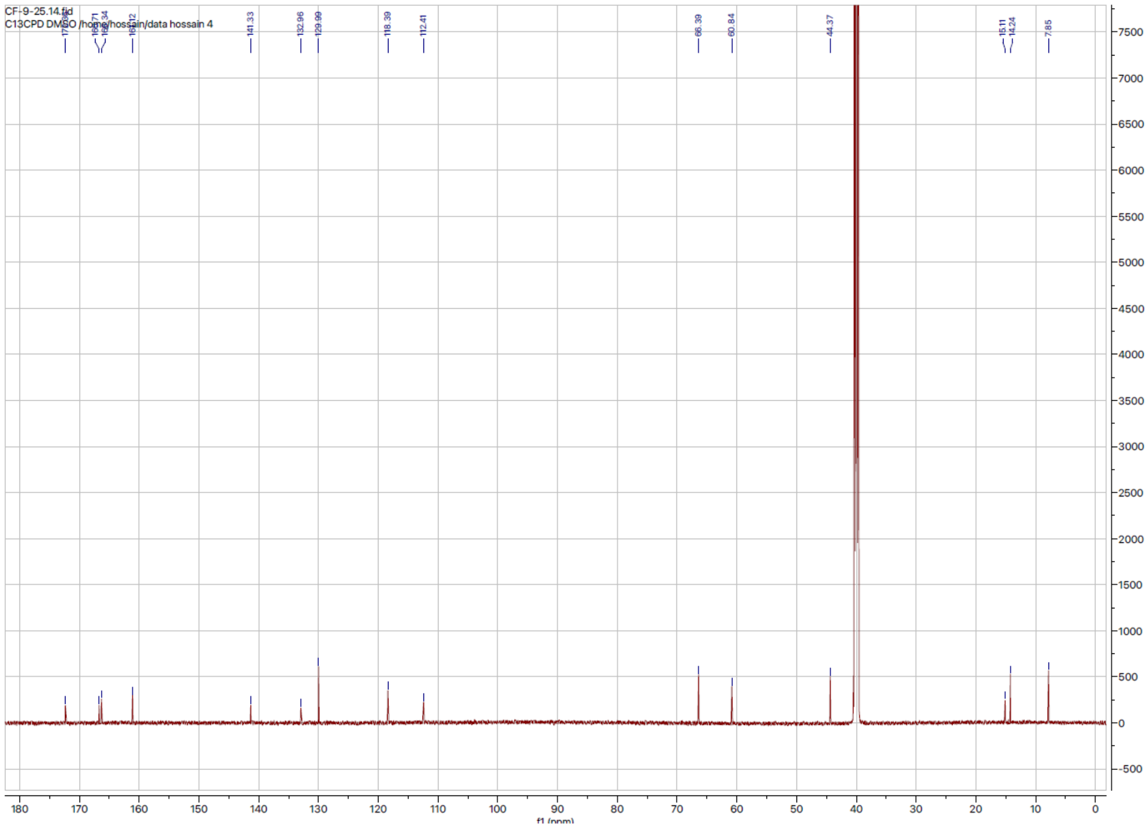

1H and 13C NMR of Compound 11

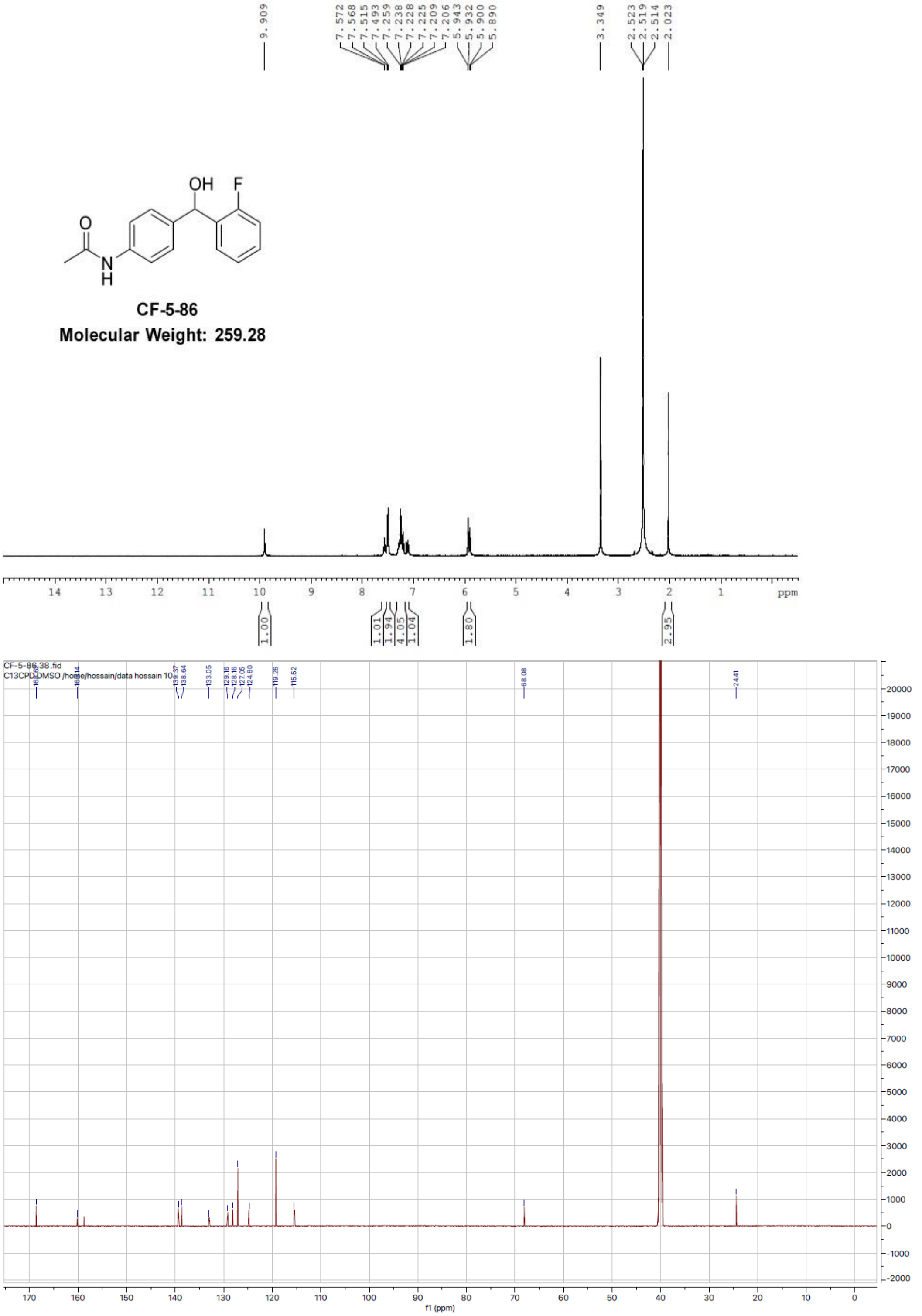

1H and 13C NMR of Compound **12**

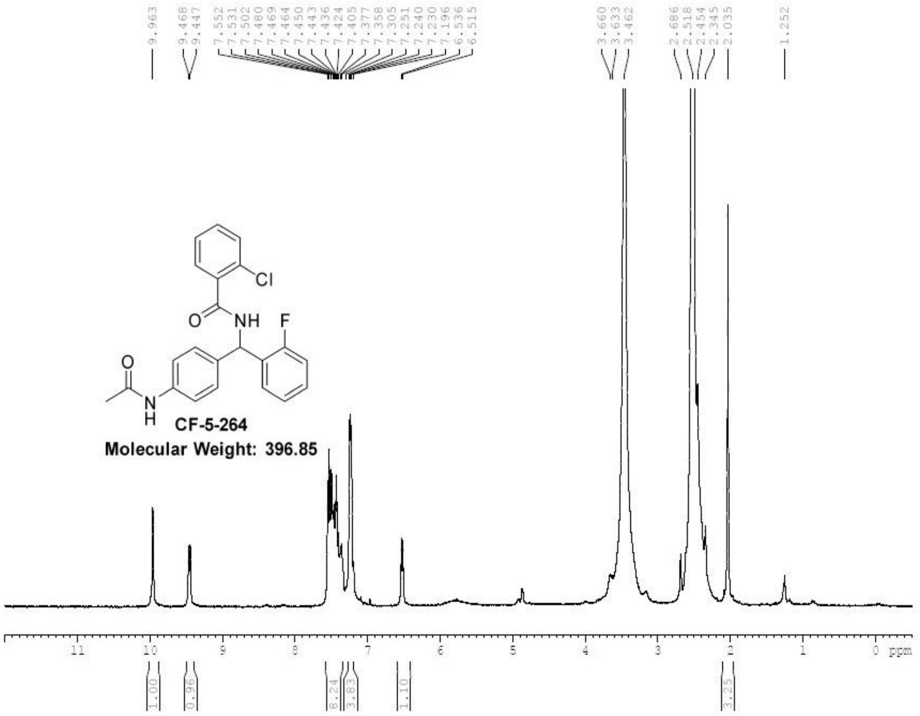
9.963
9.468
9.447
7.552
7.531
7.502
7.480
7.469
7.464
7.450
7.443
7.436
7.424
7.405
7.377
7.358
7.305
7.251
7.240
7.230
7.196
6.536
6.515
3.660
3.633
3.462
2.686
2.518
2.454
2.345
2.035
1.252
Cl
O
NH
F
O
N
H
CF-5-264
Molecular Weight: 396.85
11
10
9
8
7
6
5
4
3
2
1
0 ppm
1.00
0.96
8.24
3.83
1.10
3.25

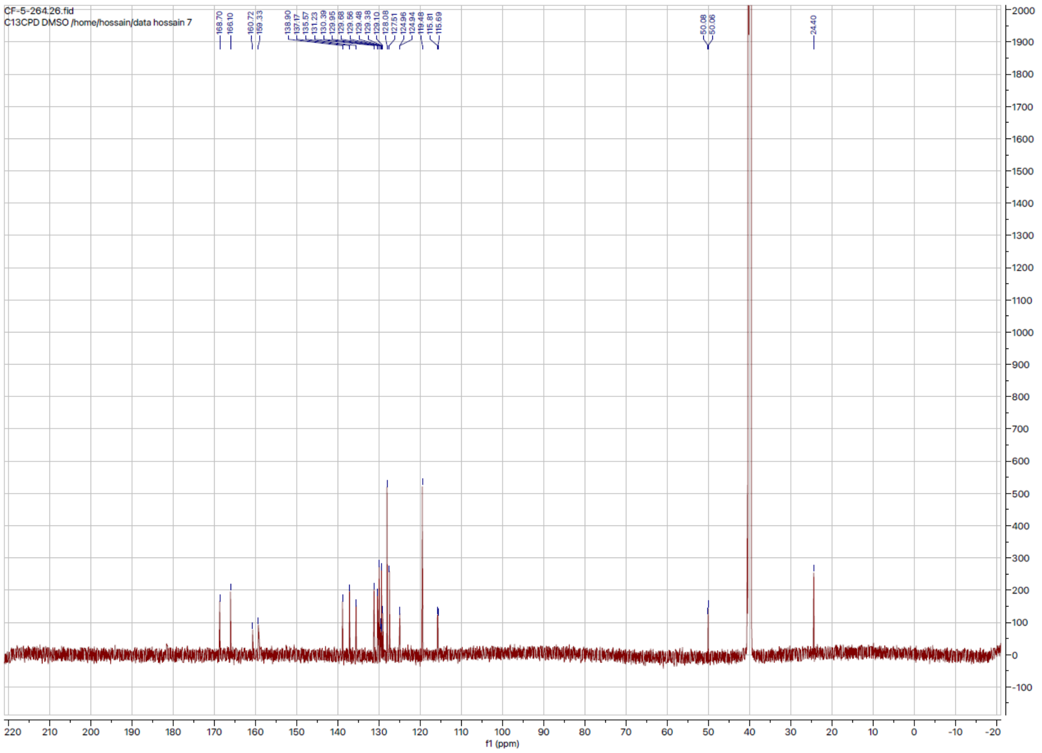
CF-5-264.26.fid
C13CPD DMSO /home/hossain/data hossain 7
168.70
166.10
160.72
159.33
138.90
137.17
135.57
131.23
130.39
129.95
129.68
129.56
129.48
129.38
128.10
128.09
127.51
124.96
124.84
119.48
115.81
115.69
50.08
50.06
24.40
f1 (ppm)

1H and 13C NMR of Compound 13

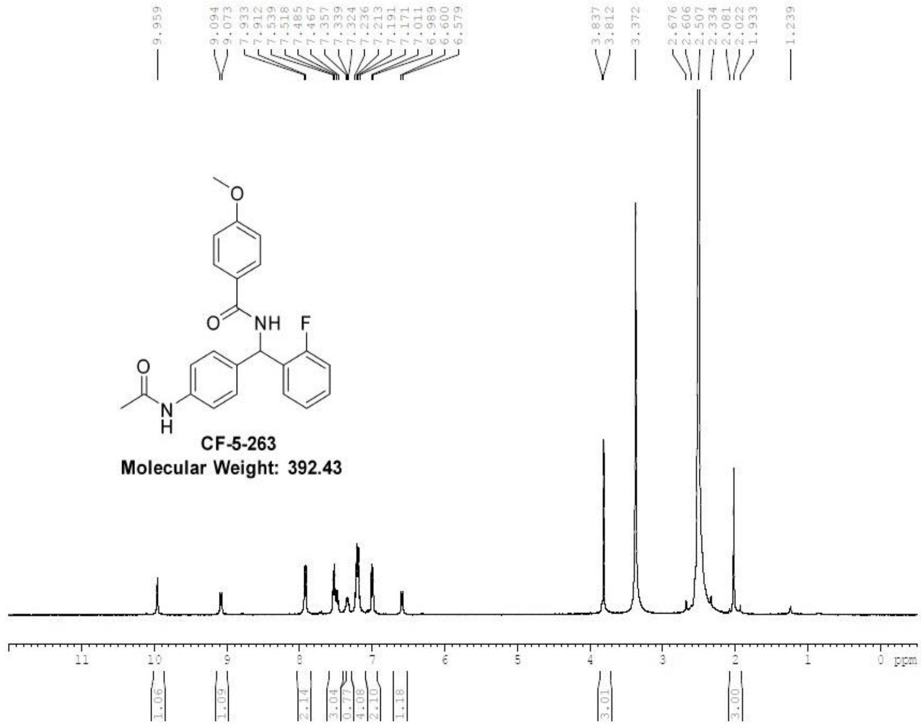

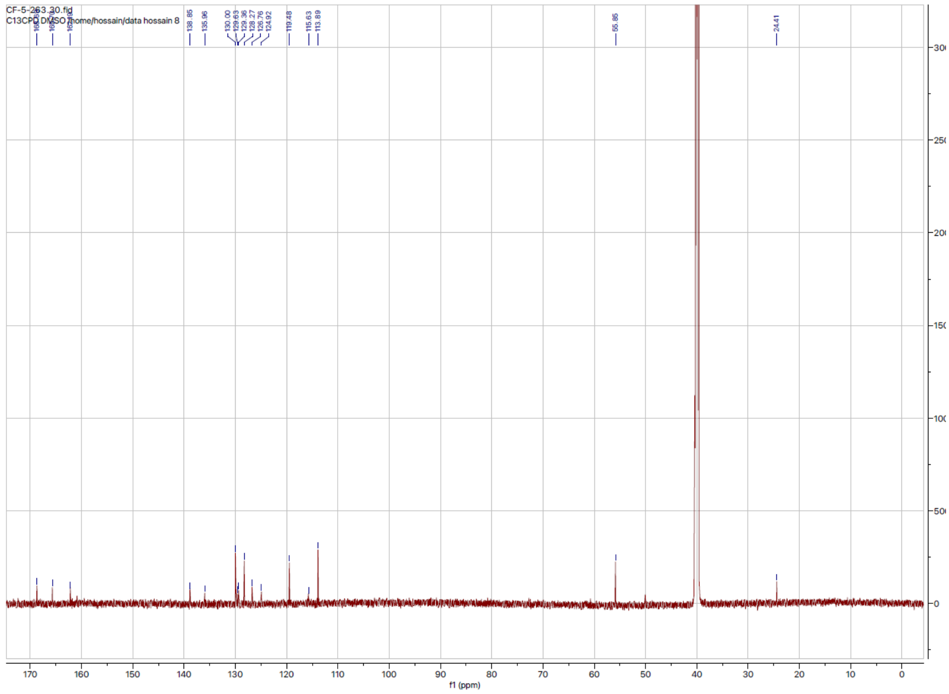

Supplement: Supplementary file 2 — Supplementary Information [file 41467_2025_56213_MOESM2_ESM.pdf]
