# Supplementary material for: Structural insights into human brachyury DNA recognition and discovery of progressible binders for cancer therapy
Source: Nat Commun. 2025 Feb 14;16:1596. doi: 10.1038/s41467-025-56213-1 (PMC11828899; doi:10.1038/s41467-025-56213-1)
Supplement: Supplementary file 3 — Description of Additional Supplementary Files [file 41467_2025_56213_MOESM3_ESM.pdf]

Description of Additional Supplementary Files

File Name: Supplementary Data 1

Description: Excel file showing the formulation of the DSI-poised library used to screen brachyury crystals. The second tab contains details of the compound soaks and outcomes.

File Name: Supplementary Data 2

Description: Excel file reporting crystallographic data collection and refinement statistics for all fragment bound structures in this study.
