# Supplementary material for: Structural insights into human brachyury DNA recognition and discovery of progressible binders for cancer therapy
Source: Nat Commun. 2025 Feb 14;16:1596. doi: 10.1038/s41467-025-56213-1 (PMC11828899; doi:10.1038/s41467-025-56213-1)
Supplement: Supplementary file 6 — Reporting Summary [file 41467_2025_56213_MOESM6_ESM.pdf]

nature portfolio

Corresponding author(s): Joseph Newman, Paul Workman & David Drewry

Last updated by author(s): Dec 20, 2024

Reporting Summary

Nature Portfolio wishes to improve the reproducibility of the work that we publish. This form provides structure for consistency and transparency in reporting. For further information on Nature Portfolio policies, see our Editorial Policies and the Editorial Policy Checklist.

Statistics

For all statistical analyses, confirm that the following items are present in the figure legend, table legend, main text, or Methods section.

n/a	Confirmed	
☐	☒	The exact sample size (*n*) for each experimental group/condition, given as a discrete number and unit of measurement
☒	☐	A statement on whether measurements were taken from distinct samples or whether the same sample was measured repeatedly
☒	☐	The statistical test(s) used AND whether they are one- or two-sided<br>*Only common tests should be described solely by name; describe more complex techniques in the Methods section.*
☒	☐	A description of all covariates tested
☒	☐	A description of any assumptions or corrections, such as tests of normality and adjustment for multiple comparisons
☐	☒	A full description of the statistical parameters including central tendency (e.g. means) or other basic estimates (e.g. regression coefficient) AND variation (e.g. standard deviation) or associated estimates of uncertainty (e.g. confidence intervals)
☒	☐	For null hypothesis testing, the test statistic (e.g. *F*, *t*, *r*) with confidence intervals, effect sizes, degrees of freedom and *P* value noted<br>*Give P values as exact values whenever suitable.*
☒	☐	For Bayesian analysis, information on the choice of priors and Markov chain Monte Carlo settings
☒	☐	For hierarchical and complex designs, identification of the appropriate level for tests and full reporting of outcomes
☒	☐	Estimates of effect sizes (e.g. Cohen's *d*, Pearson's *r*), indicating how they were calculated

Our web collection on statistics for biologists contains articles on many of the points above.

Software and code

Policy information about availability of computer code

Data collection	CCP4 crystallography suite, Phenix refine, XDS, DIALS, REFMAC, XChemexplorer, PanDDA, spectramax, Biorad imagelab, Biacore controll software
Data analysis	PanDDA, Pymol, Coot, Genialis, Trim Galore, HISAT2, samtools, Graphpad Prism, Biaevaluation

For manuscripts utilizing custom algorithms or software that are central to the research but not yet described in published literature, software must be made available to editors and reviewers. We strongly encourage code deposition in a community repository (e.g. GitHub). See the Nature Portfolio guidelines for submitting code & software for further information.

Data

Policy information about availability of data

All manuscripts must include a data availability statement. This statement should provide the following information, where applicable:

- Accession codes, unique identifiers, or web links for publicly available datasets
- A description of any restrictions on data availability
- For clinical datasets or third party data, please ensure that the statement adheres to our policy

The crystallographic coordinates and structure factor data generated in this study have been deposited in the Protein Data Bank with the following accession codes: 6F58 [https://doi.org/10.2210/pdb6F58/pdb], 6F59 [https://doi.org/10.2210/pdb6F59/pdb] , 8CDN [https://doi.org/10.2210/pdb8CDN/pdb], 5QS6 [https://doi.org/10.2210/pdb5QS6/pdb], 5QS7 [https://doi.org/10.2210/pdb5QS7/pdb], 5QS8 [https://doi.org/10.2210/pdb5QS8/pdb], 5QS9 [https://doi.org/10.2210/

pdb5QS9/pdb], 5QSA [https://doi.org/10.2210/pdb5QSA/pdb], 5QSB [https://doi.org/10.2210/pdb5QSB/pdb], 5QSC [https://doi.org/10.2210/pdb5QSC/pdb], 5QSD [https://doi.org/10.2210/pdb5QSD/pdb], 5QSE [https://doi.org/10.2210/pdb5QSE/pdb], 5QSF [https://doi.org/10.2210/pdb5QSF/pdb], 5QSG [https://doi.org/10.2210/pdb5QSG/pdb], 5QSH [https://doi.org/10.2210/pdb5QSH/pdb], 5QSI [https://doi.org/10.2210/pdb5QSI/pdb], 5QSJ [https://doi.org/10.2210/pdb5QSJ/pdb], 5QSK [https://doi.org/10.2210/pdb5QSK/pdb], 5QSL [https://doi.org/10.2210/pdb5QSL/pdb], 5QRF [https://doi.org/10.2210/pdb5QRF/pdb], 5QRG [https://doi.org/10.2210/pdb5QRg/pdb], 5QRH [https://doi.org/10.2210/pdb5QRH/pdb], 5QRI [https://doi.org/10.2210/pdb5QRI/pdb], 5QRJ [https://doi.org/10.2210/pdb5QRJ/pdb], 5QRK [https://doi.org/10.2210/pdb5QRK/pdb], 5QRL [https://doi.org/10.2210/pdb5QRL/pdb], 5QRM [https://doi.org/10.2210/pdb5QRM/pdb], 5QRN [https://doi.org/10.2210/pdb5QRN/pdb], 5QRO [https://doi.org/10.2210/pdb5QRO/pdb], 5QRP [https://doi.org/10.2210/pdb5QRP/pdb], 5QRQ [https://doi.org/10.2210/pdb5QRQ/pdb], 5QRR [https://doi.org/10.2210/pdb5QRR/pdb], 5QRS [https://doi.org/10.2210/pdb5QRS/pdb], 5QT0 [https://doi.org/10.2210/pdb5QT0/pdb], 5QRT [https://doi.org/10.2210/pdb5QRT/pdb], 5QRU [https://doi.org/10.2210/pdb5QRU/pdb], 5QRV [https://doi.org/10.2210/pdb5QRV/pdb], 5QRW [https://doi.org/10.2210/pdb5QRW/pdb], 5QRX [https://doi.org/10.2210/pdb5QRX/pdb], 5QRY [https://doi.org/10.2210/pdb5QRY/pdb], 5QRZ [https://doi.org/10.2210/pdb5QRZ/pdb], 5QS0 [https://doi.org/10.2210/pdb5QS0/pdb], 5QS1 [https://doi.org/10.2210/pdb5QS1/pdb], 5QS2 [https://doi.org/10.2210/pdb5QS2/pdb], 5QS3 [https://doi.org/10.2210/pdb5QS3/pdb], 5QS4 [https://doi.org/10.2210/pdb5QS4/pdb], 5QS5 [https://doi.org/10.2210/pdb5QS5/pdb], 7ZL2 [https://doi.org/10.2210/pdb7ZL2/pdb], 8A10 [https://doi.org/10.2210/pdb8A10/pdb], 8A7N [https://doi.org/10.2210/pdb8A7N/pdb], 7ZKF [https://doi.org/10.2210/pdb7ZKF/pdb]. Ground state datasets used for PanDDA analysis generated in this study are deposited in the Protein Data Bank under accession codes 7HI8 [https://doi.org/10.2210/pdb7HI8/pdb] and 7HI9 [https://doi.org/10.2210/pdb7HI9/pdb]. The SPR sensogram and data fits generated in this study have been made publicly available on Zenodo (https://doi.org/10.5281/zenodo.6394811). Source data are provided with this paper.

Research involving human participants, their data, or biological material

Policy information about studies with human participants or human data. See also policy information about sex, gender (identity/presentation), and sexual orientation and race, ethnicity and racism.

Reporting on sex and gender	Cell lines from chordoma foundation used in this study have been isolated from individual of mixed gender and race. Cell lines were processed identically regardless of this.
Reporting on race, ethnicity, or other socially relevant groupings	Cell lines from chordoma foundation used in this study have been isolated from individual of mixed gender and race. Cell lines were processed identically regardless of this.
Population characteristics	Not applicable
Recruitment	Not applicable
Ethics oversight	Not applicable

Note that full information on the approval of the study protocol must also be provided in the manuscript.

Field-specific reporting

Please select the one below that is the best fit for your research. If you are not sure, read the appropriate sections before making your selection.

☒ Life sciences ☐ Behavioural & social sciences ☐ Ecological, evolutionary & environmental sciences

For a reference copy of the document with all sections, see nature.com/documents/nr-reporting-summary-flat.pdf

Life sciences study design

All studies must disclose on these points even when the disclosure is negative.

Sample size	Biochemical measurements were performed on samples with at least 3 independent replicates
Data exclusions	SPR data and crystal structures are not replicated
Replication	Biochemical measurements were performed on samples with at least 3 independent replicates
Randomization	Not applicable
Blinding	not applicable

Reporting for specific materials, systems and methods

We require information from authors about some types of materials, experimental systems and methods used in many studies. Here, indicate whether each material, system or method listed is relevant to your study. If you are not sure if a list item applies to your research, read the appropriate section before selecting a response.

Materials & experimental systems

n/a	Involved in the study	
☒	☐	Antibodies
☐	☒	Eukaryotic cell lines
☒	☐	Palaeontology and archaeology
☒	☐	Animals and other organisms
☒	☐	Clinical data
☒	☐	Dual use research of concern
☒	☐	Plants

Methods

n/a	Involved in the study	
☐	☒	ChIP-seq
☒	☐	Flow cytometry
☒	☐	MRI-based neuroimaging

Eukaryotic cell lines

Policy information about cell lines and Sex and Gender in Research

Cell line source(s)	Chordoma foundation
Authentication	None of the cell lines were authenticated
Mycoplasma contamination	Not tested for Mycoplasma
Commonly misidentified lines (See ICLAC register)	NA

Plants

Seed stocks	not applicable
Novel plant genotypes	not applicable
Authentication	not applicable

ChIP-seq

Data deposition

☐ Confirm that both raw and final processed data have been deposited in a public database such as GEO.

☐ Confirm that you have deposited or provided access to graph files (e.g. BED files) for the called peaks.

Data access links *May remain private before publication.*	Chip seq data were analyzed from public databases not generated as part of this study
Files in database submission	not applicable
Genome browser session (e.g. UCSC)	not applicable

Methodology

Replicates	not applicable
Sequencing depth	not applicable
Antibodies	not applicable
Peak calling parameters	not applicable
Data quality	not applicable
Software	Genialis, Trim Galore, HISAT2, samtools
